# Structural and functional insights into extreme thermal stability and activity of two GH12 domains of a multidomain glycosidase from a hyperthermophilic euryarchaeon

**DOI:** 10.1111/febs.70095

**Published:** 2025-04-21

**Authors:** Kseniya S. Zayulina, Evgenii N. Frolov, Christina Stracke, Alexandra A. Klyukina, Anna N. Khusnutdinova, Peter Stogios, Tatiana Skarina, Alexander F. Yakunin, Peter N. Golyshin, Bettina Siebers, Tatiana E. Shugaeva, Ilya V. Kublanov

**Affiliations:** ^1^ Winogradsky Institute of Microbiology, Federal Research Center of Biotechnology Russian Academy of Sciences Moscow Russia; ^2^ Molecular Enzyme Technology and Biochemistry, Environmental Microbiology and Biotechnology (EMB), Centre for Water and Environmental Research (CWE), Faculty of Chemistry University of Duisburg‐Essen Germany; ^3^ Centre for Environmental Biotechnology, School of Environmental and Natural Sciences Bangor University UK; ^4^ Department of Chemical Engineering and Applied Chemistry University of Toronto Canada; ^5^ Department of Bioengineering and Bioinformatics Moscow State University Russia; ^6^ Science for Life Laboratory, Department of Applied Physics KTH Royal Institute of Technology Stockholm Sweden; ^7^ Institute of Environmental Sciences Hebrew University of Jerusalem Rehovot Israel

**Keywords:** cellulase, glycosidase, hyperthermophilic archaea, thermostability, thermozyme, xylanase

## Abstract

Bacteria and fungi are well known for efficient degradation of plant polysaccharides thanks to various enzymes involved in plant cell wall decomposition. However, little is known about the role of archaea in this process or the repertoire and features of their polysaccharide‐degrading enzymes. In our previous work, we discovered an archaeal multidomain glycosidase (MDG) composed of three catalytic domains (GH5 and two GH12) and two cellulose‐binding modules (CBM2). The recombinant MDG and individual GH5 catalytic domain were active against cellulose and a number of other polysaccharides at a wide range of temperatures, with optimum temperatures (*T*_opt_) of 60 °C and 80 °C, respectively. The present study was focused on the characterization of two GH12 domains of the MDG. Purified recombinant TMDG_GH12‐1 and TMDG_GH12‐2 proteins were active as individual enzymes but exhibited distinct catalytic properties. Both enzymes were thermostable and active at extremely high temperatures: TMDG_GH12‐1 was active at 40–130 °C (*T*_opt_ 100 °C), and its half‐life (*t*_½_) at 100 °C was 42 h, which makes it one of the most thermostable glycosidases known so far, whereas TMDG_GH12‐2 was active at 50–100 °C (*T*_opt_ 90 °C) with *t*_½_ at 100 °C being 30 min. Phylogenetic and structural analysis of both TMDG_GH12 proteins together with molecular docking and site‐directed mutagenesis suggested that the presence of two disulfide bridges and the W → Q mutation in the active site contribute to the exceptional thermostability of TMDG_GH12‐1. Further structural and mutational studies of the TMDG_GH12‐1 domain will help to gain a better understanding of the molecular mechanisms of its extraordinary thermostability and substrate specificity.

## Graphical abstract

The multidomain glycosidase MDG from the hyperthermophilic euryarchaeon *Thermococcus* sp. contains five protein domains: three catalytic domains (GH5, GH12‐1, and GH12‐2) and two cellulose‐binding modules (CBM2). In this study, the GH12‐1 and GH12‐2 domains were individually purified and biochemically characterized. Both proteins appeared to be endoglucanases with different substrate preferences and exhibited extreme thermostability. Structural and mutational analyses of GH12 domains provided insights into their active sites and thermostability mechanisms.

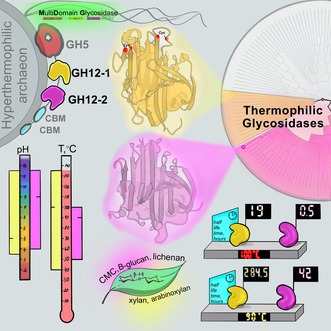

**Abbreviations**
CAPS: 3‐(cyclohexylamino)‐1‐propanesulfonic acid
CBM: carbohydrate‐binding modules
CMC: carboxymethyl cellulose
DEAE: diethylaminoethanol
DNA: deoxyribonucleic acid
DNSA: 3,5‐dinitrosalicylic acid
DTT: dithiothreitol
EDTA: ethylenediaminetetraacetic acid
FPLC: fast protein liquid chromatography
GF: gel filtration
GH: glycoside hydrolase/glycosidase
HEPES: 4‐(2‐hydroxyethyl)‐1‐piperazineethanesulfonic acid
HMM: hidden Markov model
kDa: kilodaltons
*K*_M_
: Michaelis constant
MDG: multidomain glycosidase
MES: 2‐(*N*‐morpholino)ethanesulfonic acid
MOPS: 3‐(*N*‐morpholino)propanesulfonic acid
MW: molecular weight
MWCO: molecular weight cut‐off
PASC: phosphoric acid swollen cellulose
PCR: polymerase chain reaction
PDB: protein data bank
PEG400: polyethylene glycol 400
PF: purification factor
pLDDT: predicted local distance difference test
RMSD: root mean square deviation
SDS: sodium dodecyl sulfate
TCS: Transitive Consistency Score
TLC: thin layer chromatography
Tris: tris(hydroxymethyl)aminomethane

## Introduction

Thermozymes, the enzymes from thermophiles, are promising biocatalysts due to their high activities and stabilities at elevated temperatures [1]. In addition to thermal stability, thermozymes are often capable of withstanding other harsh conditions, such as pressure [2], low and high pH [3], and high concentrations of detergents and denaturing agents [4, 5].

Glycosidases and cellulases are among the most commonly employed and demanded enzymes in industry [6, 7, 8]. Most known thermostable cellulose‐degrading enzymes were isolated from thermophilic bacteria of *Thermoanaerobacterium*, *Dictyoglomus*, *Thermotoga*, or *Caldicellulosiruptor* genera [9, 10, 11, 12, 13]. At the same time, Archaea dominated terrestrial and marine hydrothermal systems with temperatures around 100 °C. Some of them are known to be capable of growing on complex polysaccharides [14, 15, 16] making them a promising source of novel hyperthermostable glycosidases. However, the number of characterized archaeal polysaccharide‐degrading enzymes is still comparatively low [17] mainly due to difficulties in the cultivation of novel thermophilic archaea as well as the low availability of archaeal genetic tools [18].

Effective hydrolysis of cellulose requires coordinated action of a number of proteins including endoglucanases (EC 3.2.1.4), exoglucanases (EC 3.2.1.74) beta‐glucosidases (EC 3.2.1.21) as well as other glycosidases, carbohydrate phosphorylases, and carbohydrate‐binding modules (CBM) [19, 20, 21]. Endoglucanases, which randomly hydrolyze their internal glycosidic bonds, play a crucial role as their action leads to considerable depolymerization of cellulose, thereby tremendously increasing the amount of substrates available for enzymes that act at the termini of the polyglucan molecules [22].

To date, 13 endoglucanases from hyperthermophilic archaea are characterized [3, 14, 23, 24, 25, 26, 27, 28, 29, 30] (Table S1). One of them contains a catalytic domain GH5, which is a part of an archaeal multidomain glycosidase (MDG), but it was also active as an individual enzyme [30]. Albeit the complete MDG and its truncated versions, including the individual GH5 domain, were characterized, the activity of the other two catalytic domains of MDG (both are GH12s) as single proteins has not so far been investigated. MDG acts mainly as an endoglucanase and β‐(endo)‐mannosidase with various side activities, including xyloglucanase, xylanase, and beta‐1,3‐glucanase. Comparative analysis of the activities of complete MDG and its truncated versions allowed to propose GH5 domain as an endoglucanase with side activities, while the two GH12 domains were predicted to be an exoglucanase and an endoglucanase, providing additional multifunctionality to the MDG [30]. However, since no GH12 domains of MDG were obtained as a single active enzyme, their characteristics remained elusive. The purpose of this work was to close this gap. Both GH12 domains were isolated and purified as individual polypeptides designated as TMDG_GH12‐1 and TMDG_GH12‐2, and their structures were resolved. Both TMDG_GH12‐1 and TMDG_GH12‐2 exhibited high thermostabilities and high‐temperature optima of activity; however, their pH optima and substrate spectra were different: while both were capable of hydrolyzing barley β‐glucan, lichenan, and carboxymethyl cellulose (CMC) TMDG_GH12‐1 was also active with xylan and arabinoxylan. Phylogenetic and structural analyses of TMDG_GH12‐1 and TMDG_GH12‐2 and their homologs, as well as the investigation of enzymatic properties of the TMDG_GH12‐2 mutant variants, provided new insights into the evolution and distribution of these enzymes within the archaeal domain, as well as on the molecular mechanisms of their thermostability.

## Results

### *In silico* analysis of the MDG TMDG_GH12‐1 and TMDG_GH12‐2 domain sequences

**Fig. 1:**
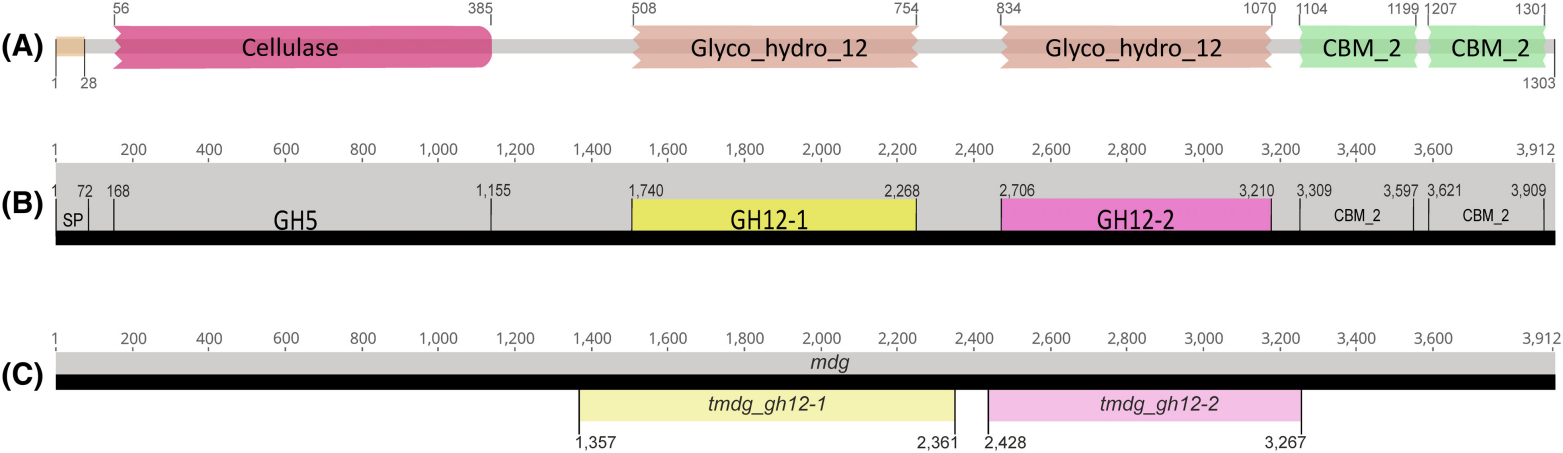
Domain organization of a multidomain glycosidase from *Thermococcus* sp. strain 2319x1. (A) Coordinates of catalytic domains within the MDG sequence; (B) Location of the coding sequences of GH12 catalytic domains within the *MDG* gene sequence. (C) Coordinates of *tmdg_gh12‐1* and *tmdg_gh12‐2* with flanked regions on the *mdg* gene nucleotide sequence.

According to HMMscan, the TMDG_GH12‐1 and TMDG_GH12‐2 coordinates on the *in silico* translated complete MDG protein were 508–754 (247 aa) and 834–1070 (237 aa), respectively, that corresponded to the following coordinates on the nucleotide sequence of MDG: 1740–2268 for TMDG_GH12‐1 and 2706–3210 for TMDG_GH12‐2 (Fig. 1A,B). The primers for cloning each individual GH12 enzyme were designed to capture also the flanked regions of the domains resulting in the products, corresponding to the following coordinates on the *mdg*: 1357–2361 for *tmdg_gh12‐1* and 2428–3267 for *tmdg_gh12‐2* (Fig. 1C).

The sequence lengths of *tmdg_gh12‐1* and *tmdg_gh12‐2* were 1005 and 840 base pairs, and the respective polypeptides comprise 335 and 280 aa. Accordingly, both TMDG_GH12 polypeptides in this work were slightly longer than predicted earlier [30]. Amino acid sequence similarity and identity between the TMDG_GH12‐1 and TMDG_GH12‐2 aligned together with other GH12 proteins were 28 ± 1% and 18 ± 1%, respectively, depending on the multiple alignment approach used for phylogenetic analysis (six approaches were used, see below). Calculated characteristics of GH12 proteins (Table S2) revealed that TMDG_GH12‐1 is slightly larger than TMDG_GH12‐2, slightly less soluble, and has a higher pI. Among the most notable differences in amino acid composition between the two polypeptides was the absence of cysteines in TMDG_GH12‐2, while four cysteine residues were present in TMDG_GH12‐1. These cysteines were located in pairs (CxxxxC) on the N and C termini of the protein.

**Fig. 2:**
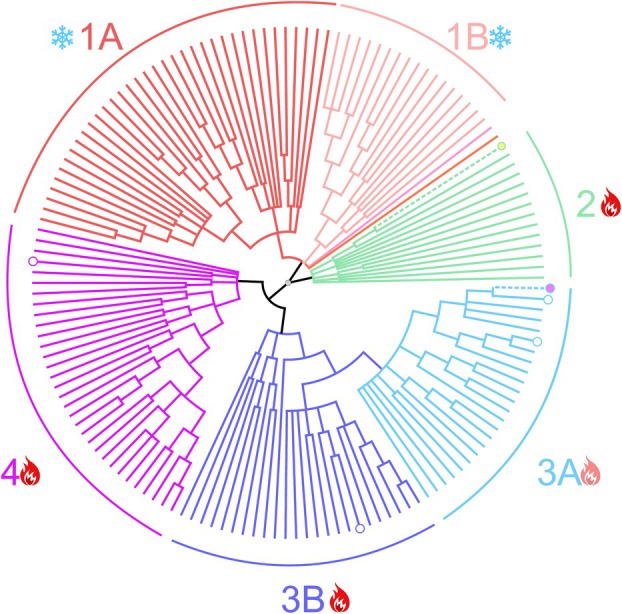
Maximum likelihood phylogenetic tree of GH12 family glycosidases. The tree is a consensus tree constructed from two phylogenetic trees inferred using mafft and clustal_omega multiple sequence alignments. Six deeply rooted clusters are numbered and colored. Bold yellow and purple circles are TMDG_GH12‐1 and TMDG_GH12‐2, respectively. White circles – biochemically characterized proteins (only that purified from hyperthermophilic archaea are shown). Signs near the clusters designations demonstrate temperature adaptation of the enzymes based on the temperature optima of the characterized enzymes and the optimal temperature of growth of the organisms from which these sequences were obtained: red fire (2, 3B and 4) for thermozymes and hyperthermophiles; slightly transparent fire (3A) for the cluster with both thermophiles and mesophiles; snowflake (1A, 1B) for mesophiles. Detailed information on the protein sequences is on Fig. S1 and Table S7.

The nearest relative of TMDG_GH12‐2 was a biochemically characterized endoglucanase EglA from *Pyrococcus furiosus* (Q9V2T0, 82% and 66% of identity when the sequences from pairwise or multiple sequence alignments were compared, respectively). TMDG_GH12‐1 amino acid sequence was mostly identical to the uncharacterized protein from *Ignisphaera aggregans* (A0A7J3YTE7, 61% and 34% of identity when the sequences from pairwise or multiple sequence alignments were compared, respectively) while the closest characterized protein was the same endoglucanase EglA from *P. furiosus* (Q9V2T0, 29% and 19% of identity when the sequences from pairwise or multiple sequence alignments were compared, respectively). Phylogenetic analysis (Fig. 2) of GH12 enzymes revealed an archaeal origin of both TMDG_GH12; however, they affiliated with two different clusters. The cluster with TMDG_GH12‐1 consists of hypothetical glycoside hydrolases from Crenarchaeota, while the TMDG_GH12‐2 cluster is formed by proteins from Euryarchaeota and Bacteria, some of which were characterized biochemically as endoglucanases (Fig. S1).

### Expression and purification of individual TMDG_GH12‐1 and TMDG_GH12‐2 domains

After heterologous expression of *tmdg_gh12‐1* and *tmdg_gh12‐2* in *Escherichia coli* BL21(DE3) and three steps of purification (Table 1), homogeneous enzyme preparations were obtained. The purification factor (PF) of TMDG_GH12‐1 glycosidase was 44.4, and the yield was 37.9%. The experimentally determined mass of the denatured protein was 42 kDa (Fig. S2A), which is consistent with the theoretical mass of this domain. For TMDG_GH12‐2 glycosidase, PF was 54.5, and the yield was 34.9%. Its experimentally determined mass of 37 kDa (Fig. S2B) also correlated well with the theoretical mass of this domain. Both domains were purified in their monomeric state, and they did not tend to oligomerize, which was confirmed by size‐exclusion chromatography (Fig. S3A,B). The determined native molecular masses of TMDG_GH12‐1 and TMDG_GH12‐2 were 43 kDa and 26 kDa, respectively (Table S3).

**Table 1:** Purification of TMDG_GH12‐1 and TMDG_GH12‐2 enzymes. Activity measurements were done with 1% AZO‐CMC at optimal assay conditions for the respective enzyme (for TMDG_GH12‐1 at 100 °C, pH 5.0, 50 mm MES; for TMDG_GH12‐2 at 90 °C, pH 9.0, 50 mm Tris/HCl).

Fraction of purification	Volume (mL)	Total protein (mg)	Total activity (U)	Specific activity (U·mg^−1^ protein × 1000)	Yield (%)	Purification factor (PF)
**TMDG_GH12‐1**						
Cell extract	6.85	272.63	7.26 ± 0.36	26.6 ± 1.3	100	1
Ni‐NTA	3.5	6.37	3.92 ± 0.2	615 ± 30.8	54	23.1
DEAE Sephadex	1.8	3.42	2.68 ± 0.13	784 ± 39.2	36.9	29.5
Sephadex G‐100	2.5	2.33	2.75 ± 0.14	1180 ± 59.0	37.9	44.4
**TMDG_GH12‐2**						
Cell extract	6	168	62.81 ± 3.14	373 ± 15.7	100	1
Ni‐NTA	6.5	7.36	45.25 ± 2.26	6148 ± 149	72.1	16.5
DEAE Sephadex	2	4.32	27.95 ± 1.4	6470 ± 286	44.4	17.3
Sephadex G‐100	3	1.08	21.94 ± 1.1	20 315 ± 984	34.9	54.5

### Enzymatic activity of purified TMDG_GH12‐1 and TMDG_GH12‐2 proteins

#### Substrate specificity

Among all tested carbohydrates, both TMDG_GH12s were capable of hydrolyzing barley β‐glucan, CMC, and lichenan. TMDG_GH12‐1, but not TMDG_GH12‐2, was also active with xylan and arabinoxylan (Table 2). The highest activity of both domains was detected with barley β‐glucan, which was further used to elucidate conditions of enzymatic activity and stability.

**Table 2:** Substrate specificity of TMDG_GH12‐1 and TMDG_GH12‐2. na, no activity.

Substrate	Specific activity U·mg^−1^ protein	
	**TMDG_GH12‐1**	**TMDG_GH12‐2**
Barley β‐glucan	156 ± 1.3	3766 ± 87
Lichenan	30.6 ± 4.1	1225 ± 96
CMC	8.9 ± 0.9	83 ± 19
AZO‐CMC	1.18 ± 0.06	20.3 ± 1.0
Arabinoxylan	13.5 ± 0.6	na
Xylan	2.4 ± 0.25	na
AZO‐xylan	1.62 ± 0.02	na

#### Influence of temperature and pH on activity of TMDG_GH12‐1 and TMDG_GH12‐2 domains

**Fig. 3:**
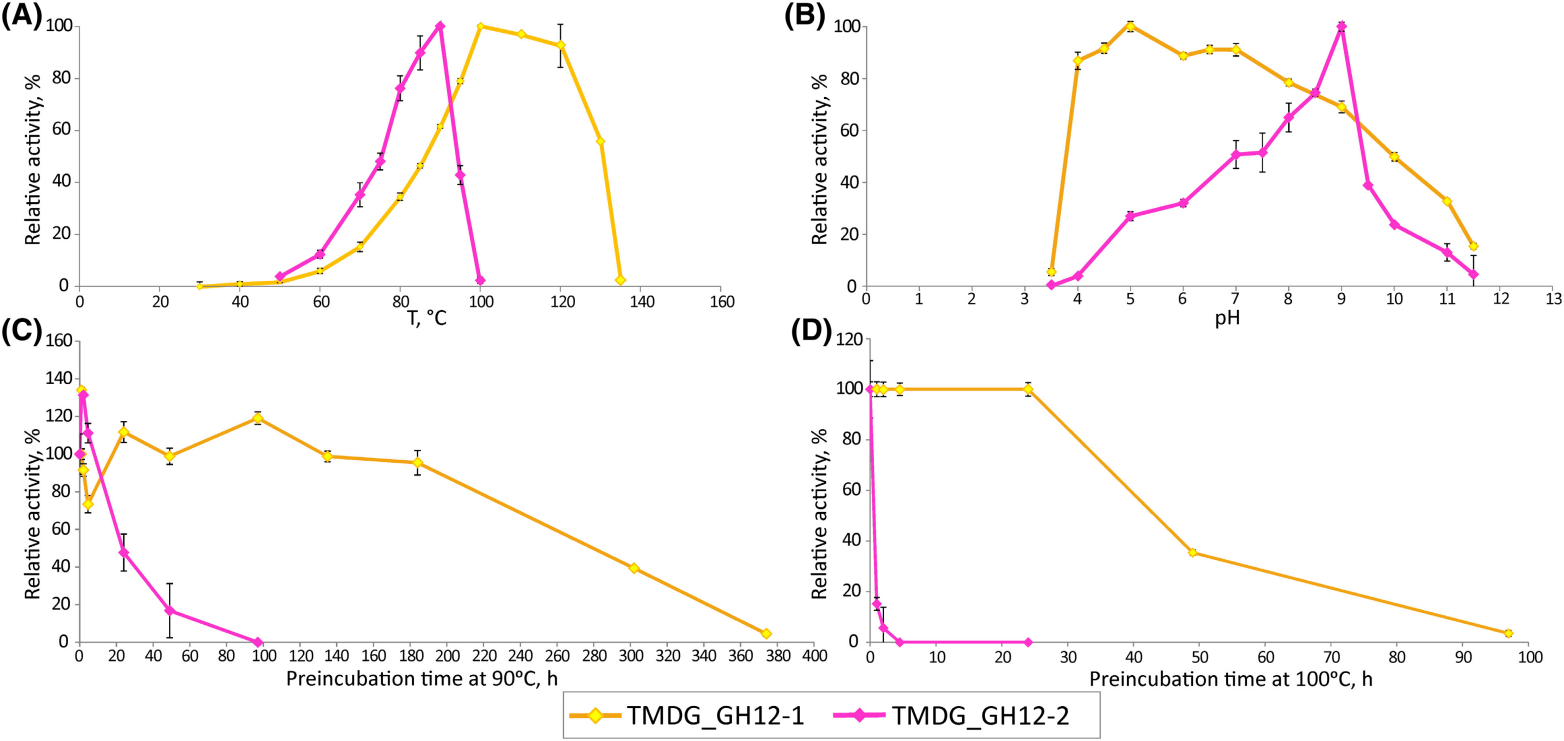
Characterization of recombinant TMDG_GH12‐1 and TMDG_GH12‐2. (A) Temperature dependence of activity; (B) pH dependence of activity; (C) Thermostability after preincubation at 90 °C; (D) Thermostability after preincubation at 100 °C. The error bars on all plots are standard deviations (SD). All measurements were made in triplicates.

At optimal pH (5.0) TMDG_GH12‐1 was active between 40 °C and 130 °C with maximal activity at 100 °C (Fig. 3A). No activity was detected at 35 °C or 135 °C. At the optimal temperature (100 °C) the enzyme showed a broad pH range of pH 3.5–11.5 with the highest activity at pH 5.0 (Fig. 3B). At the optimal pH (9.0) TMDG_GH12‐2 was active between 50 °C and 100 °C with the maximal activity at 90 °C (Fig. 3A). No activity was detected at 30 °C and 110 °C. At the optimal temperature (90 °C) TMDG_GH12‐2 showed a broad pH range of 4.0–11.5 with the highest activity at pH 9.0 (Fig. 3B).

#### Kinetic parameters and end products of hydrolysis

The *K*_M_ and *V*_max_ (Figs S4 and S5) of hydrolysis of barley β‐glucan were 1.60 mg·mL^−1^ and 160.2 (μmol·min^−1^)·mg^−1^ protein for TMDG_GH12‐1 and 1.93 mg·mL^−1^ and 2949.68 (μmol·min^−1^)·mg^−1^ protein for TMDG_GH12‐2, respectively. Both enzymes were capable of hydrolyzing lichenan and CMC albeit at lower levels of activity. TMDG_GH12‐1 but not TMDG_GH12‐2 also hydrolyzed arabinoxylan, xylan, and AZO‐xylan (Table 3). For both enzymes, the highest *k*_cat_/*K*_M_ values were observed with barley β‐glucan and lichenan. TMDG_GH12‐1 hydrolyzed arabinoxylan more efficiently than xylan and AZO‐xylan, but in all cases, the *k*_cat_/*K*_M_ values were rather low in comparison to polysaccharides with β‐1,4 – glucose backbone.

**Table 3:** Kinetic parameters of TMDG_GH12‐1 and TMDG_GH12‐2 glycosidases. Activities were determined at 100 °C, pH 5.0 for TMDG_GH12‐1, and at 90 °C, pH 9.0 for TMDG_GH12‐2. na, no activity.

Substrate	Type(s) of glycosidic linkage and main sugar monomers	*K*_M_ (mg·mL^−1^)		*V*_max_ ((μmol·min^−1^)·mg^−1^ protein)		Catalytic constant, *k*_cat_ (s^−1^)		Specificity constant, *k*_cat_/*K*_M_ (mL·(mg s)^−1^)	
		**TMDG_GH12‐1**	**TMDG_GH12‐2**	**TMDG_GH12‐1**	**TMDG_GH12‐2**	**TMDG_GH12‐1**	**TMDG_GH12‐2**	**TMDG_GH12‐1**	**TMDG_GH12‐2**
CMC	β‐1,4 – glucose backbone	5.49 ± 0.91	2.81 ± 0.04	17.55 ± 0.32	83.01 ± 19.55	12.29 ± 0.22	511.93 ± 7.31	2.24	182.37
AZO‐dyed CMC		4.23 ± 0.53	2.19 ± 0.04	1.31 ± 0.03	56.91 ± 2.17	0.92 ± 0.02	350.94 ± 6.38	0.22	160.36
Alpha‐cellulose		No activity							
PASC									
Barley β‐glucan	Mixed β‐1,3 and β‐1,4, glucose backbone	1.60 ± 0.05	1.93 ± 0.06	160.20 ± 3.18	2949.68 ±121.53	56.07 ± 1.11	1818.97 ± 56.40	35.08	944.40
Lichenan		1.11 ± 0.03	1.03 ± 0.04	53.41 ± 3.63	1149.61 ±147.91	37.39 ± 2.54	708.93 ± 27.53	33.61	688.22
Beechwood xylan	β‐1,4 xylose backbone, 4‐O‐methyl‐glucuronic acid sidechains	1.11 ± 0.17	na	3.14 ± 0.04	na	2.20 ± 0.03	na	1.97	na
AZO‐xylan (beechwood)		2.04 ± 0.27	na	2.36 ± 0.16	na	1.39 ± 0.09	na	6.86	na
Arabinoxylan (oat spelt)	β‐1,4 xylose backbone, arabinose and xylose sidechains	2.28 ± 0.40	na	28.02 ± 2.12	na	3.92 ± 0.30	na	1.72	na

Using thin layer chromatography, the following products of hydrolysis of barley β‐glucan, CMC, and lichenan by TMDG_GH12‐1 were detected: glucose (C1), cellobiose (C2), cellotriose (C3), cellotetraose (C4) and cellohexaose (C6). The products of hydrolysis of the same substrates by TMDG_GH12‐2 were C1 to C4 on β‐glucan and C1 to C3 on CMC and lichenan (Fig. S6). The products of hydrolysis of xylan by TMDG_GH12‐1 were xylose and a mixture of oligomers ranging from xylodiose to xylohexaose (X2–X6) as well as some undetermined higher molecular weight xylooligosaccharides.

#### Thermostability of TMDG_GH12‐1 and TMDG_GH12‐2 domains

Fifty percentage activity losses (i.e. half‐life time (*t*_½_) of activity) of TMDG_GH12‐1 and TMDG_GH12‐2 at 90 °C were recorded after enzyme incubation for 284.5 and 19 h, respectively (Fig. 3C,D). At 100 °C, a 50% loss in activity was observed after 42 h (TMDG_GH12‐1) and 0.5 h (TMDG_GH12‐2) of preincubation. TMDG_GH12‐1 was completely inactivated after 374 h at 90 °C and after 102 h at 100 °C. No activity of TMDG_GH12‐2 protein was observed after 135 h of incubation at 90 °C and after 4.5 h at 100 °C.

#### Influence of metal ions, detergents, reducing and denaturing agents on activity

Catalytic activity of TMDG_GH12‐1 was not influenced by Na^+^, Mg^2+^ and Zn^2+^ cations, while Ca^2+^, K,^+^ and Fe^2+^ partially reduced (74.8%, 58.2% and 57.7%, respectively) and Ag^+^, Cu^2+^, Fe^3+^ and Mn^2+^ significantly reduced or completely inhibited the activity of the enzyme (Table 4). EDTA and urea did not influence the activity of TMDG_GH12‐1, while β‐mercaptoethanol and DTT reduced it twofold. On the contrary, Twin80 and SDS increased the activity of the enzyme.

**Table 4:** Influence of metal ions, detergents, reducing, and denaturing agents on the activities of TMDG_GH12‐1 and TMDG_GH12‐2. na, no activity.

Concentration, mm	Relative activity (% ± SD)	
	**TMDG_GH12‐1**	**TMDG_GH12‐2**
NaCl, 5	91.8 ± 7	80.9 ± 5.3
KCl, 5	58.2 ± 3.5	83.7 ± 5.5
MnCl_2_, 5	4.68 ± 0.9	128 ± 6
CaCl_2_, 5	74.8 ± 3.4	117 ± 7.4
MgCl_2_, 5	90.0 ± 3.1	109 ± 7.9
AgNO_3_, 1	6.23 ± 0.8	88.4 ± 6.2
CuCl_2_, 1	3.51 ± 0.5	99.9 ± 7.2
ZnSO_4_, 1	85.5 ± 4.8	109 ± 5.4
FeCl_3_, 1	na	89 ± 7.8
FeCl_2_, 1	57.7 ± 2.7	107 ± 11
Urea, 5	96.6 ± 2.2	69.7 ± 5.1
EDTA, 5	99.1 ± 3.2	57.3 ± 5
Tween 80, 5	142 ± 1.1	83.5 ± 5.1
SDS, 5	137 ± 23.3	99.9 ± 10.7
β‐Mercaptoethanol, 5	46.5 ± 2	52.7 ± 4.4
DTT, 5	51.8 ± 4.4	67.9 ± 4.9

The activity of TMDG_GH12‐2 was not influenced by K^+^, Na^+^, Ag^+^, Cu^2+^ and Fe^3+^ ions, while divalent cations (Fe^2+^, Mg^2+^ and Zn^2+^, Mn^2+^ and Ca^2+^) slightly increased it. Addition of urea, DTT, EDTA, Tween 80, and β‐mercaptoethanol partially reduced activity, while SDS had no influence on the activity of TMDG_GH12‐2 (Table 4).

### Structural analysis of TMDG_GH12‐1 and TMDG_GH12‐2 domain proteins

**Fig. 4:**
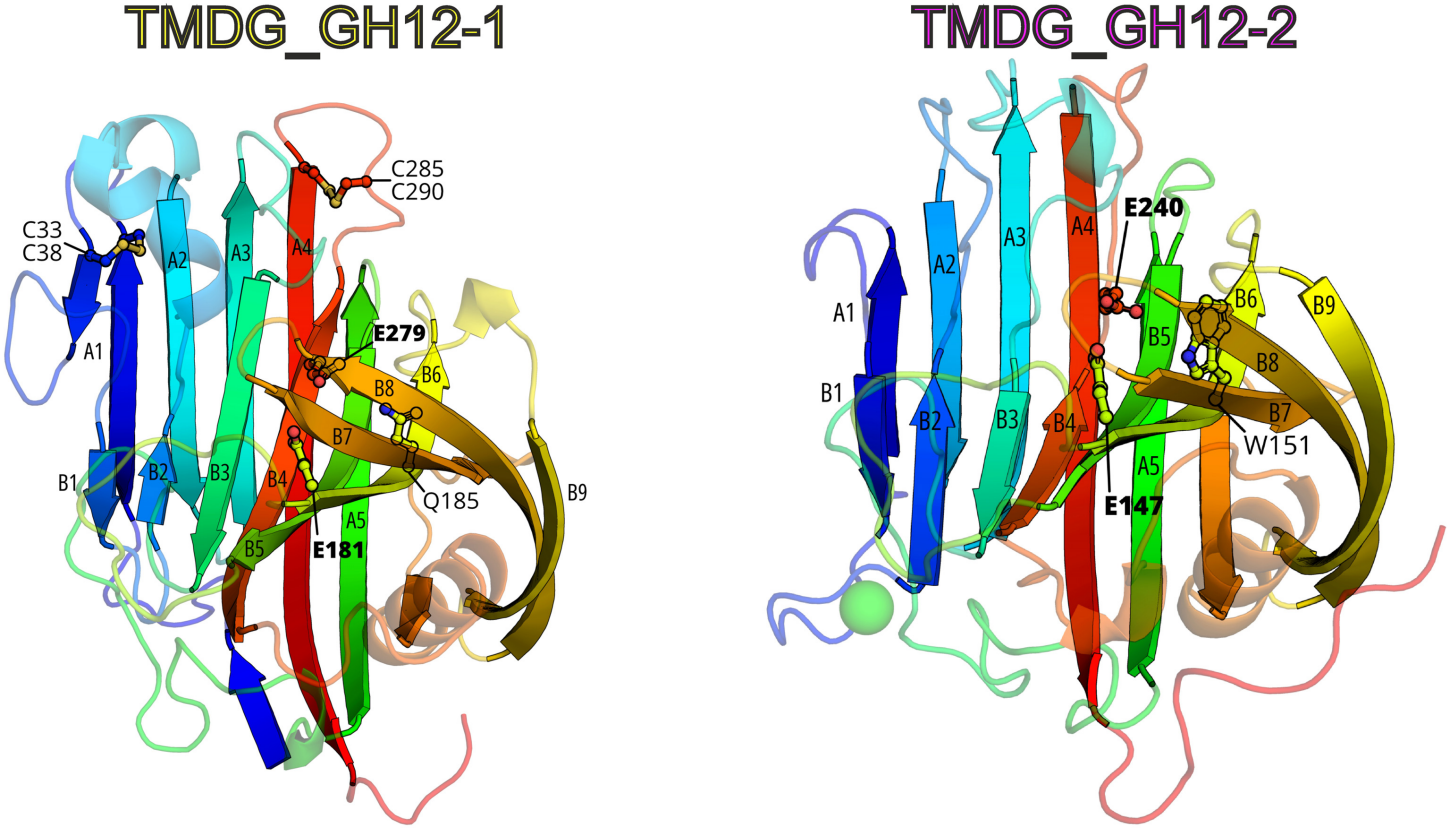
Structural models of TMDG_GH12 domains. Alpha Fold 2 model of the TMDG_GH12‐1 domain (left) and the model from the crystal structure of the TMDG_GH12‐2 domain (PDB ID, 7S8K) (right). Catalytic glutamates are labeled in bold. Cysteine residues and W → Q substituted residues, suggested to be crucial for the thermostability of TMDG_GH12‐1, are labeled in normal font. The green sphere represents the bound Ca^2+^ ion. This figure was generated using pymol 2.5.0 (https://pymol.org/).

Purified TMDG_GH12‐1 and TMDG_GH12‐2 were submitted to crystallization trials, and TMDG_GH12‐2 was found to produce diffracting crystals. The crystal structure of TMDG_GH12‐2 was determined using molecular replacement to resolution of 2.55 Å (Table S4). Since TMDG_GH12‐1 produced no diffracting crystals, a high‐quality structural model of this enzyme was generated using AlphaFold2 (AF2) [31]. The structure of TMDG_GH12‐2 and the structural model of TMDG_GH12‐1 (Fig. 4) both have beta‐jelly roll folds [32] known for all studied structures of the GH12 family enzymes [33].

Dali [34] search for structural homologs of TMDG_GH12‐2 revealed several GH12 structures. The top hits include *P. furiosus* EGPf (PDB code 3VGI, *Z* score 46.4, root mean square deviation (RMSD) 0.6 Å, 80% sequence identity), *Thermotoga maritima* 12A (3AMM, *Z* score 37.1, RMSD 1.3 Å, 35% sequence identity) and *Rhodothermus marinus* Cel12A (2BW8, *Z* score 26.2, RMSD 2.0 Å, 27% sequence identity).

### Active site of TMDG_GH12‐2

The glycosyl binding site forms a cavity along the entire protein. It is approximately 30 Å long, 9 Å wide, and 10 Å deep. The site is capable of binding up to six glucose residues: from −4 to +2 according to standard numbering relative to the cleavage site [35]. Most of the internal sidewall surface is formed by residues W12, W34, W71, V72, and I157 on one side and by W85, W130, W191, Y193, and I189 on the other side of the crevice (Fig. S7). Hydrophobic residues Y75, M149, A160, and W151 are also present at the bottom of the cavity. However, most of the deep‐located residues are polar: there are seven glutamate residues (two of which are catalytic: E240 and E147), three asparagines (N32, N86, N88), and one lysine (K83). Side chains of five residues near the catalytic site—D18, D20, D22, E26, and D92—are predicted to participate in Ca^2+^ ions binding.

### Active site of TMDG_GH12‐1

Since no diffracting crystals were obtained for TMDG_GH12‐1, a structural model was created using AlphaFold2 (the model was choosen with the highest pLDDT score). TMDG_GH12‐1 comprises also a beta‐jelly roll fold but had slightly longer loops between the beta‐strands (Fig. 4). It lacked the Ca^2+^ binding site but had two predicted disulfide bridges between the cysteines, located in pairs on the N‐ and C‐termini of the protein (C33–C38 and C285–C290). The upper side of the catalytic cavity is formed by E162, E181, W120, W164, W 230, F232, K118, and T58 and is similar to TMDG_GH12‐2 (Fig. S8), with the exception that one tryptophan (W151) in TMDG_GH12‐2 was replaced with glutamine Q185 in TMDG_GH12‐1. The internal ventral surface is formed by four glutamate residues E60, E279, E112, and E189, aromatic residues W43, W49, W64, W66, Y105, Y110, Y281, and V51; moreover, three tryptophan residues and tyrosine are shifted from the catalytic ones (E279, E181) towards the non‐reducing end in comparison to TMDG_GH12‐2 (Fig. S9). Also, the bottom edge of the crevice TMDG_GH12‐1 is complementary to TMDG_GH12‐2 but has an additional Trp residue W43, which extends the cavity towards the non‐reducing end.

### Molecular docking of oligosaccharides

Both the experimentally obtained structure of TMDG_GH12‐2 domain and the predicted AlphaFold2 structure of TMDG_GH12‐1 domain were used for molecular docking (performed with Rosetta GlycanDock package) to explore the binding of polysaccharides within the catalytic cavity. Cellohexaose and xylohexaose molecules were used as ligands for each of the two GH12 domains. Their monomers (glycosyl moieties) were numbered as −4, −3, −2, −1, +1, +2, according to their position relative to the catalytic site with bond cleavage occurring between the −1 and +1 sugar residues [35]. The distance between the catalytic oxygen atom of E147 in TMDG_GH12‐2 and the C1 atom of the −1 glycosyl residue is 3.9 Å, which is close to the corresponding distance of 3.6 Å in TmCel12A – a homologous cellulase 12A from *T. maritima* (PDB ID: 3AMM [33]).

**Fig. 5:**
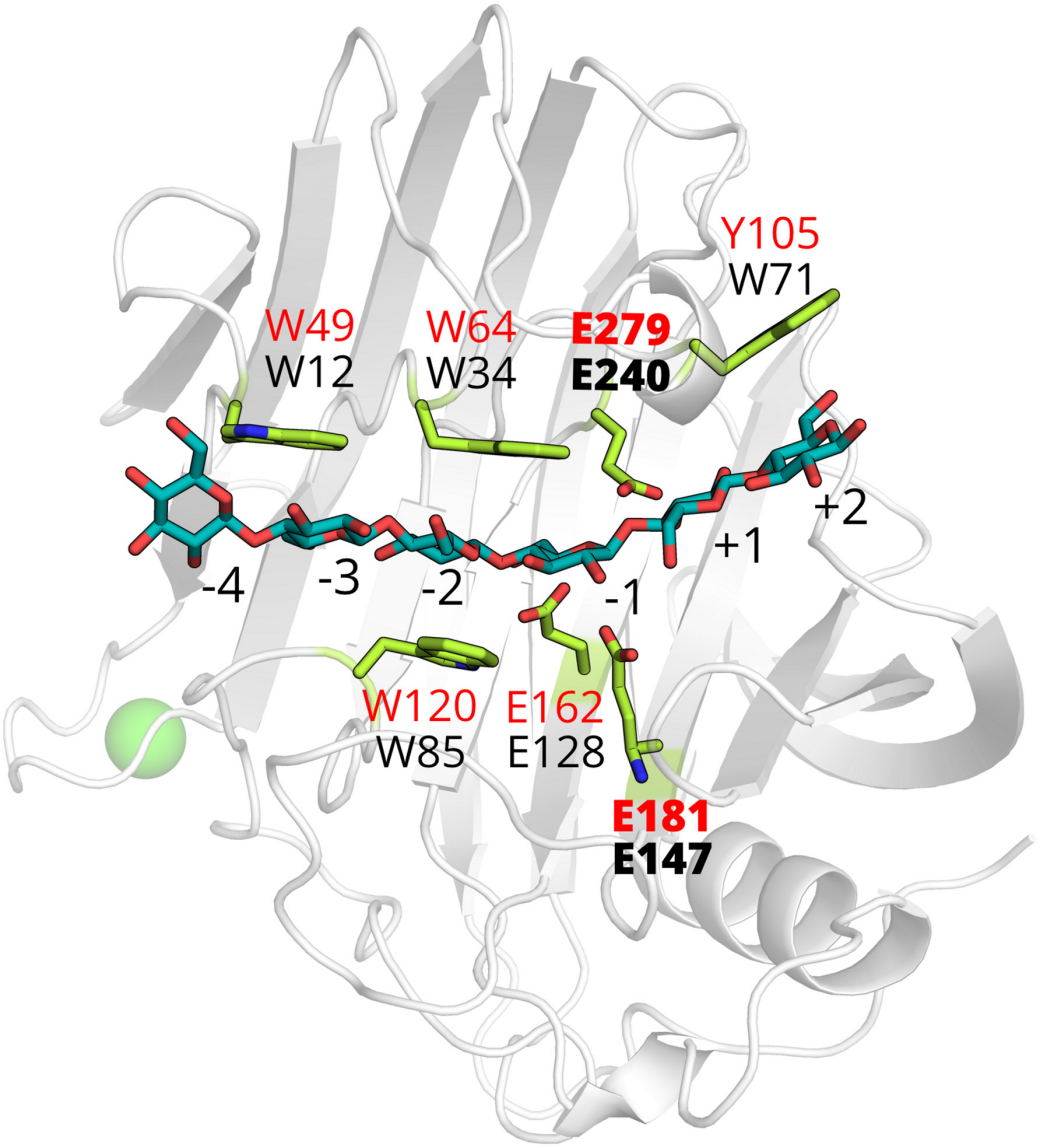
Cellohexaose docking into the active sites of TMDG_GH12‐2 domain. The protein ribbon diagram of TMDG_GH12‐2 is shown in gray with the key active site residues shown as sticks with green colored carbons and labeled black (respective residues of TMDG_GH12‐1 are labeled red). Cellohexaose molecule is shown as sticks with teal colored carbons, whereas the green sphere represents the bound Ca^2+^ ion. Catalytic glutamates are labeled in bold. This figure was generated using pymol 2.5.0 (https://pymol.org/).

The major part of the protein‐ligand interface in TMDG_GH12‐2 is formed by the catalytic residues E147 and E240 as well as aromatic (W12, W34, W71 and W85) and polar (N32, N86, K83, E30, E77, and E128) residues, which are necessary for ligand stabilization (Fig. 5, Table S5). The ligand‐binding interface of TMDG_GH12‐1 was similar (Fig. S10): the catalytic (E181, E279), aromatic (W43, W49, W64, W120) and polar and charged (K118, E60, E112, N122, E162) residues are involved in ligand stabilization.

Binding to the −2 and −3 glycosyl moieties of the substrate could be enabled by N32, W85, and K83 in TMDG_GH12‐2 (N62, N122 and K118 in TMDG_GH12‐1). The K83 position is further stabilized by E30 and E77 in TMDG_GH12‐2 and E60 and E275 in TMDG_GH12‐1. Stable conformation of the complex is also maintained by a hydrogen bond network between the amino acids in the catalytic crevice. E128 has a hydrogen bond with W130, E147 with W191, E240 with N124 (E162 and W164, E181 and W230, E279 and N158 in TMDG_GH12‐1 respectively) (Fig. S10).

Stabilizing the −3 glycosyl moiety in both TMDG_GH12 could be provided by tryptophans and polar amino acids (W12, W34 and N32 in TMDG_GH12‐2 (Fig. 5) and W49, W64 and N62 in TMDG_GH12‐1). The sandwich stacking of the −2 glycosyl moiety between W34 and W85 (W49, W64 and W120 in TMDG_GH12‐1, respectively) is preserved in all complexes except xylohexaose bound to TMDG_GH12‐1.

Hydrophobic stacking of W71 with the ligand is observed in TMDG_GH12‐2 complexes, but a similar interaction was not found in TMDG_GH12‐1. Despite the residue Y105 in TMDG_GH12‐1 being located in a similar position to W71 in TMDG_GH12‐2 position its side chain is oriented towards the outer periphery of the catalytic cavity, making it less likely to interact with this ligand.

The molecular docking of xylohexaose revealed the ligand binding conformations analogous to those observed for cellohexaose. Despite the absence of the hydroxymethyl group, which results in a lack of hydrogen bonding with the −1 and −3 glycosyl moieties in both TMDG_GH12‐1 and TMDG_GH12‐2, the overall shape and spatial orientation of the ligand in relation to the catalytic groove remain consistent. Docking TMDG_GH12‐1 with xylohexaose helps to reveal additional residues located less than 4 Å away from the ligand that could be involved in ligand binding: V51, E112, N122, and M183. However, in total, no major differences that would explain the substrate specificity of TMDG_GH12‐1 and TMDG_GH12‐2 were noticed. Probably, the interactions mentioned above with the glycosyl moieties at positions +2, −3, −4, and −5 may modulate activity, but the limitations of the current docking procedure do not allow showing reproducible bonding patterns at the peripheral regions of the catalytic crevice. Since short polysaccharide fragments were used for docking, this could lead to increased flexibility of the terminal sugar residues, providing less compatible docking results (see Discussion). Moreover, AlphaFold2 prediction of the TMDG_GH12‐1 structure demonstrates variations in the orientation of several side chains of conserved residues in the catalytic groove as compared to TMDG_GH12‐2 and other known GH12 family structures. These variations might impact the docking results for TMDG_GH12‐1, and it is unclear whether they are meaningful or are artifacts of the AlphaFold2 structure prediction. The refinement of the structure of TMDG_GH12‐1 would substantially enhance the ability to investigate its interaction with the ligand.

### Site‐directed mutagenesis of TMDG_GH12‐2

**Fig. 6:**
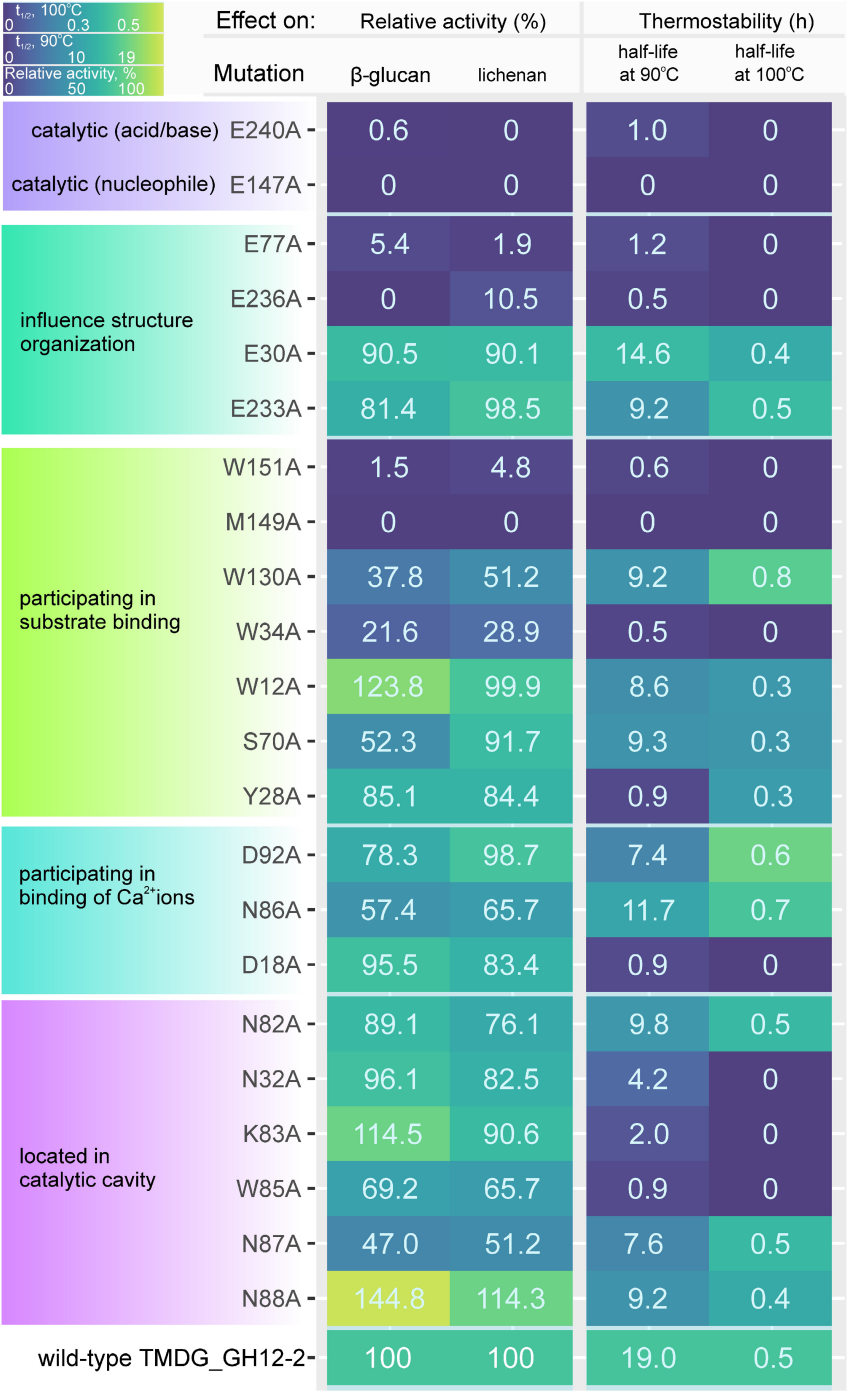
Activity and thermostability of the TMDG_GH12‐2 mutants obtained during alanine scanning. The activity is shown as a percentage of specific activity of the wild‐type TMDG_GH12‐2 after one‐step purification, which was 237 ± 6.1 and 162 ± 5.2 U·mg^−1^ protein with β‐glucan and lichenan, respectively. The half‐life time of the preparation of the wild‐type TMDG_GH12‐2 was 19 and 0.5 h at 90 °C and 100 °C, respectively. The putative roles of the residues subjected to mutations are shown in the left column. Mutants activity measurements were performed in triplicates, and the data with SD values are presented in Table S6.

To provide further insight into the role of the TMDG_GH12 active site residues in substrate binding and hydrolysis, 22 conserved and non‐conserved residues of TMDG_GH12‐2 were selected for alanine replacement, including the catalytic glutamates E147 and E240. TMDG_GH12‐2 was chosen for mutational analysis because the crystal structure of this domain was determined. Selected amino acids were binned into five groups (Fig. 6): (a) catalytic, (b) influenced the structural organization, (c) participated in substrate binding, (d) participated in Ca^2+^ binding, and (e) located in the catalytic cavity. The importance of most of them was approved by molecular docking with cellohexaose as the ligand. To confirm these predictions, we replaced each one of these 22 amino acid residues with alanine using site‐directed mutagenesis, followed by a comparative analysis of the activity and thermostability of the mutant proteins with the wild‐type TMDG_GH12‐2. Specific activities and thermostability of the mutant proteins were measured on barley β‐glucan and lichenan at the optimal for wild‐type TMDG_GH12‐2 conditions (*T* = 90 °C, pH = 9.0). Measurements of β‐glucan hydrolysis showed that substitutions of two catalytic glutamates (E147, E240) as well as E236 and M149 with alanine resulted in total inactivation of the enzyme. Almost no activity (5.4% and 1.5% of activity of wild TMDG_GH12‐2) was observed after mutations in sites E77 and W151, respectively. Alanine substitutions of N87, W34, and W130 caused the reduction of enzyme activity for up to 47.0%, 21.6%, and 37.8% of the wild‐type protein, respectively. Activity was also decreased from 69.2% to 52.3% in W85A, N86A, and S70A mutants. A slight negative effect (decrease to 78–89%) on activity was observed after mutagenesis of the following amino acid residues: D92, E233, Y28, and N82. No influence on activity (90.5–96.1%) was observed in the case when E30, D18, and N32 were substituted with alanine. Finally, a slight positive effect of mutations in K83, W12, and N88 (114.5%, 123.8% and 144.8% of activity, respectively) was observed (Table S6). The influence of alanine substitutions on lichenan hydrolysis by mutant proteins was similar to their influence on β‐glucan (Fig. 6; Table S6). A slight reduction of activity towards lichenan in comparison with β‐glucan hydrolysis was observed for N88, W12, K83, N32, D18, N82, and W85. Substitutions in E233, W130, S70, N87, D92, and N86 sites gave a small positive effect for lichenan hydrolysis compared with the same on β‐glucan. No stimulating effect on the activity of mutants was observed with the addition of Ca^2+^ (Table S6).

Thermostability of mutant proteins was tested in a similar manner as it was done for the wild‐type proteins with preincubation at 90 °C for 17 days and 100 °C for 4 days and showed that at 90 °C the half‐life time of the mutants D18A, Y28A, N32A, W34A, E77A, K83A, W85A, W151A and E236A was greatly reduced in the range from 0.47 to 4.23 h in comparison with the *t*_½_ = 19 h for the wild‐type TMDG_GH12‐2 (Fig. 6). The mutations in D92, N87, W12, E233, N88, S70, W130 and N82 decreased the half‐life‐time of the enzyme by a half (up 7.4 to 9.75 h), while substitutions in N86 and E30 reduced it slightly (11.7 and 14.6 h, respectively). Eleven of twenty‐two mutants (E147A, K83A, N32A, W151A, M149A, E77A, D18A, W85A, W34A, E236A and E240) were completely inactivated during preincubation at 100 °C. In turn, eight mutations (W12A, Y28A, E30A, S70A, N82A, N87A, N88A, and E233A) slightly lowered the half‐life time at 100 °C while three mutants (N86A, D92A and W130A) retained *t*_½_ higher (0.7, 0.6 and 0.8 h, respectively) than wild‐type enzyme (0.5 h).

**Fig. 7:**
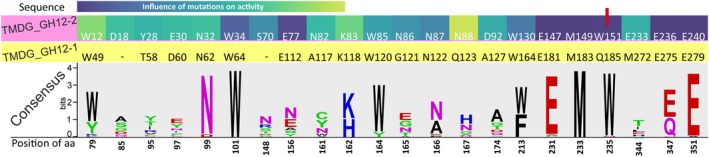
Distribution of conservation of TMDG_GH12‐2 mutated amino acid positions within the sequences of all characterized GH12 proteins including TMDG_GH12‐1 and TMDG_GH12‐2. The heatmap shows the effect of these mutations on the activity of TMDG_GH12‐2 (same to that shown in the Fig. 6). Yellow: homologous positions in TMDG_GH12‐1. Numbers below LOGO: positions on a consensus sequence. Red arrow points at W235 (consensus coordinates) position.

Among the mutated residues was a W235 (consensus coordinates), which is situated in the catalytic cavity near the acid/base glutamate residue and which is conserved in all GH12s (including TMDG_GH12‐2) except TMDG_GH12‐1 where it is replaced with glutamine (Q185). Substitution of tryptophan with alanine at this position (W151 → A151) resulted in complete inactivation of the TMDG_GH12‐2 enzyme (Figs 6 and 7). Cross‐mutagenesis was performed at this position in both studied GH12 proteins to verify whether this W → Q substitution contributes to thermostability: in TMDG_GH12‐1 Q185 was replaced with tryptophan while in TMDG_GH12‐2 an opposite mutation W151 → Q was performed.

**Fig. 8:**
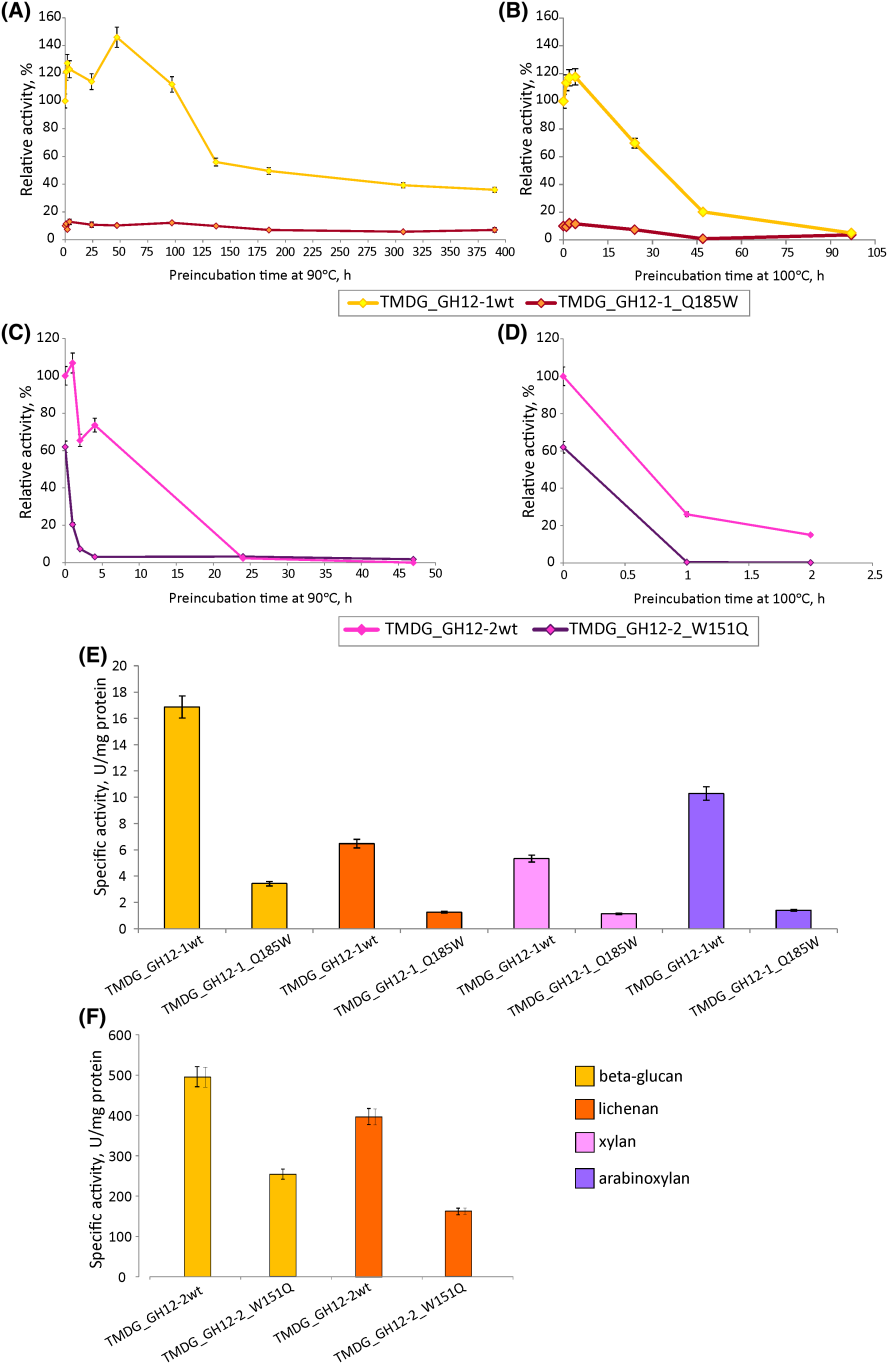
Comparison of thermostabilities and activities of the wild‐type enzymes (TMDG_GH12‐1wt, TMDG_GH12‐2wt) and the mutants (TMDG_GH12‐1_Q185W and TMDG_GH12‐2_W151Q). (A–D) Relative activity as a function of the preincubation time at 90 °C and 100 °C, for TMDG_GH12‐1 (A, B) and TMDG_GH12‐2 (C, D) respectively. (E, F) Difference in specific activity of the mutant enzymes in comparison to the corresponding activity of the wild‐type enzymes TMDG_GH12‐1 and TMDG_GH12‐2 after 1 step of purification (see the Materials and methods section for details). All the measurements were made in triplicates. The error bars indicate standard deviations (SD).

The substitution Q185 → W in TMDG_GH12‐1 reduced the activity of the enzyme from five (on beta‐glucan, lichenan or xylan) to seven times (on arabinoxylan, Fig. 8E). Moreover, it also negatively affected the TMDG_GH12‐1 glycosidase thermostability: its half‐life time at 90 °C decreased from 284.5 to 150 h and at 100 °C from 42 to 25 h (Fig. 8A,B).

The effect of the opposite W151Q substitution in TMDG_GH12‐2 was also negative. The activity of the mutant protein towards beta‐glucan and lichenan was down approximately by a half (Fig. 8F) and its half‐life at 90 °C decreased from 19 to 12 h, and at 100 °C from 30 to 20 min (Fig. 8C,D).

## Discussion

Endoglucanases (EC 3.2.1.4) are crucial enzymes for cellulose degradation but also participate in the hydrolysis of many other beta‐1,4‐linked glucans. In CAZy [36] this activity could be found in 14 of 171 known GH families: GH5‐10, GH12, GH44‐45, GH48, GH51, GH74, GH124, GH148. These families are unequal in both the abundance (i.e., the total number of known representatives) and the number of endoglucanases among other known or predicted activities. The GH5 and GH12 are the only two families with EC 3.2.1.4 as the most representative activity [37, 38]. In all families, archaea are represented in much less number than bacteria; moreover, in 8 of 14 families, there are no verified or predicted archaeal proteins.

**Fig. 9:**
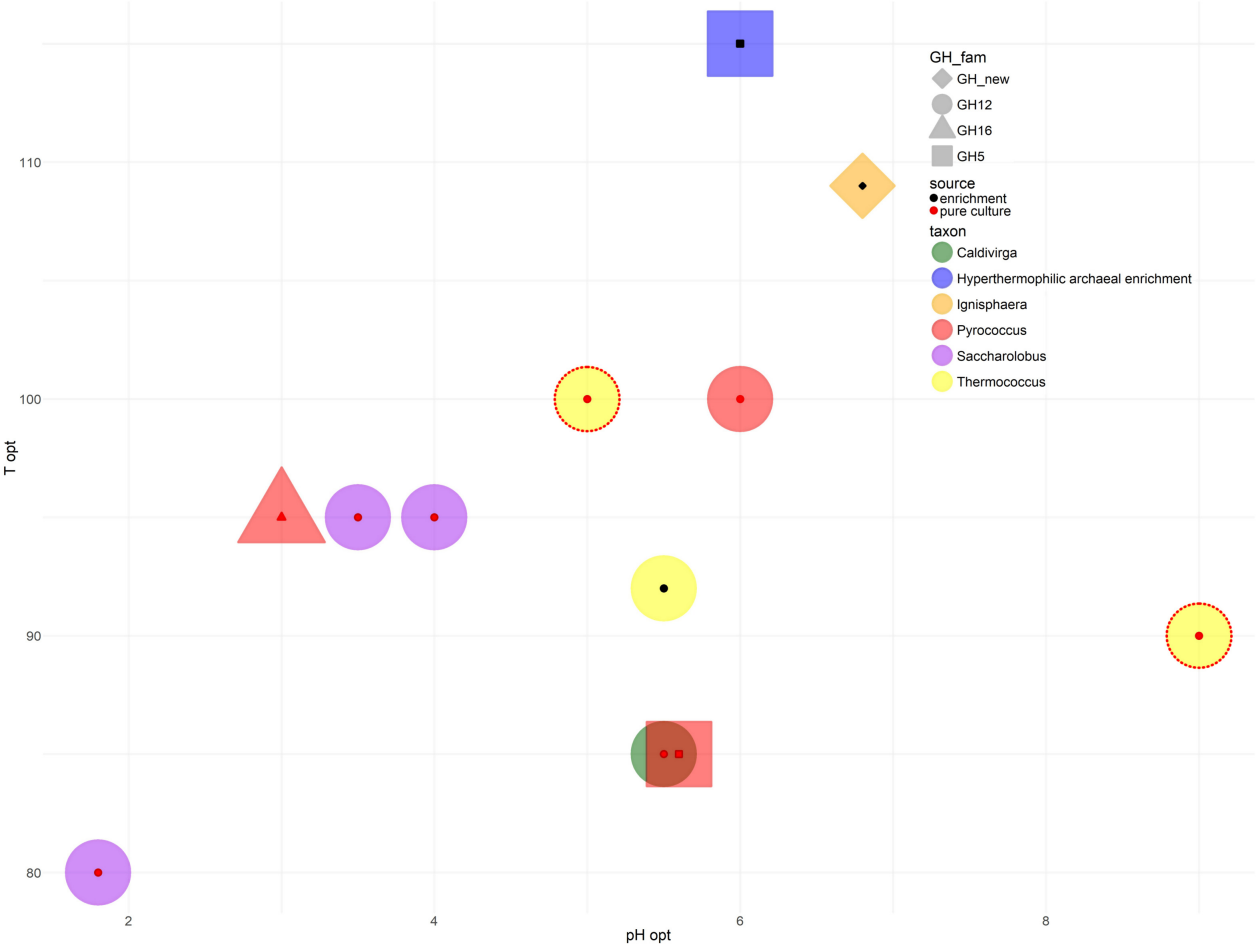
Characterized endoglucanases (EC 3.2.1.4) from hyperthermophilic archaea. The enzymes, isolated from pure or enrichment cultures, are indicated by red or blue symbols in the center of the larger shapes, respectively. Large shapes indicate the CAZy families that these endoglucanases represent: circle, GH12; triangle, GH16; square, GH5; rhombus, new family. The color of the large shape indicates the genus‐level affiliation of an archaeon from which the respective endoglucanase was isolated: *Caldivirga*, green; unknown genus, blue; *Ignisphaera*, orange; *Pyrococcus*, red; *Saccharolobus*, purple; *Thermococcus*, yellow. *Y* axis, endoglucanase *T*_opt_ of activity; *X* axis, endoglucanase pH_opt_ of activity. TMDG_GH12‐1 and TMDG_GH12‐2, yellow circles enclosed by dotted lines.

Only 13 biochemically characterized endoglucanases from hyperthermophilic archaea have been characterized so far (Fig. 9). They belong to GH12 (6 enzymes), GH5 (5), GH16 (1) and a putative novel family of glycosidases (1). In most cases, they were isolated from pure cultures of Euryarchaeota or Crenarchaeota representatives, and their temperature optima vary from 80 °C to 115 °C and the pH optima are in the moderately acidic to neutral range (Table S1).

Previously we isolated and characterized a multidomain glycosidase (MDG) containing three catalytic domains (GH5‐GH12‐GH12) and two binding modules (CBM2‐CBM2 [30]). GH12‐1 was proposed to be an endoglucanase and an endoxylanase while TMDG_GH12‐2 was suggested to be an exoglucanase with side beta‐glucosidase activity. However, as GH12 domains of the MDG were not obtained as individual active proteins, the questions about their substrate specificities and activities remain open. In the present work, these two GH12 enzymes were isolated as individual active enzymes and characterized. Despite being encoded by the same gene, the two TMDG_GH12 exhibited different temperature and pH optima: TMDG_GH12‐1 was optimally active at 100 °C and pH 5.0, while TMDG_GH12‐2 at 90 °C and pH 9.0 (Fig. 3). The latter is the first notion of a cellulase from a hyperthermophilic archaeon being optimally active at alkaline pH (Fig. 9). Whereas several cellulases that are active at high pH and temperatures > 50 °C are known (mostly representatives of GH5 family from mesophilic bacteria [39, 40, 41, 42, 43]), cellulases from thermophiles are mostly active at neutral‐acidic pH [44]. Among characterized GH12 enzymes with temperature activity optimum > 45 °C (Table S7) only LC‐CelA [45] has a pH optimum at alkalic conditions—at pH 8.0. Hence, TMDG_GH12‐2 is the second cellulose with alkaline pH optimum, but it has higher pH and a much higher temperature optimum than LC‐CelA.

The difference in temperature and pH optima of activity of the two studied TMDG_GH12 proteins correlates with the low similarity of their primary structures. Their nearest relatives were also different; however, the nearest biochemically characterized enzyme was the same: endoglucanase EglA from *P. furiosus* (Q9V2T0).

**Fig. 10:**
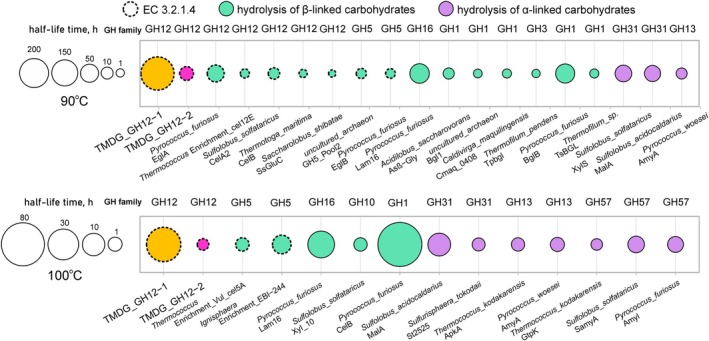
Thermostability of the most thermostable glycosidases from hyperthermophilic archaea and bacteria. Top panel, *t*_½_ at 90 °C; bottom panel, *t*_½_ at 100 °C. The size of the circles represents the half‐life time in hours. Glycosidases catalyzing the hydrolysis of beta‐ or alpha‐linked carbohydrates are colored green or purple, respectively. TMDG_GH12‐1 and TMDG_GH12‐2 are in yellow and pink, respectively. Circles with dashed lines point at endoglucanases (EC 3.2.1.4) while circles with normal line—all other glycosidases. Enzyme families are shown above the circles; microorganisms from which these enzymes were isolated are shown below the circles.

Phylogenetic analysis of all proteins assigned to the GH12 family available in UniProt revealed that they form four clusters (Fig. 2). Taking into account the divergence of the family and numerous horizontal transfers of its representatives, the relationship between the clusters cannot be established, but the affiliation of certain sequences to the clusters and intracluster evolution can be assessed. The first cluster (1A, B on Fig. 2) consists of bacterial and eukaryotic xyloglucan‐specific endoglucanases active at moderate temperatures; the second cluster including TMDG_GH12‐1 comprises endoglucanases from extremely hyperthermophilic Crenarchaea (TMDG_GH12‐1 is the only characterized and the only euryarchaeal representative in this cluster). The third cluster, including TMDG_GH12‐2, also originated from crenarchaeal enzymes which were later horizontally transferred to other archaea and bacteria (3A, B on Fig. 2), some of which are moderate thermophiles or even mesophiles (e.g., Spirochaetes or Lokiarchaeota). The fourth cluster represents only high‐temperature archaeal endoglucanases. These multiple horizontal gene transfers followed by adaptation of the respective proteins to the host habitats might be one of the reasons for reduced thermal adaptation of TMDG_GH12‐2 compared to TMDG_GH12‐1. Indeed, despite the nearest to TMDG_GH12‐2 characterized relatives – EglA from *P. furiosus* and Cel12E from a *Thermococcus* enrichment culture – being optimally active at 100 and 115 °C, respectively (i.e., their temperature optima are comparable with and even higher than of the TMDG_GH12‐1) their thermostability is much lower than that of TMDG_GH12‐1 (Fig. 10). This seems to be an indication that protein thermostability is a more evolutionarily inert property, compared to changes in temperature optima for catalysis. Indeed, it is reasonable to assume that thermostability in larger extend to be determined by features of the entire molecule rather than by specific substitutions influencing catalytic properties, which evolve much faster [46, 47] While our phylogenetic data do fit this concept, below we provide evidence supporting drastic changes in thermostability linked with certain amino acid substitutions.

Apart from their optimal activities at extremely high temperatures, the most remarkable characteristic of enzymes from hyperthermophilic Archaea is their exceptional thermostability. Both TMDG_GH12s are likewise highly thermostable proteins. This is especially true for TMDG_GH12‐1, which keeps 50% of its activity for about 11 days at 90 °C and 1.75 days at 100 °C, making it one of the most thermostable glycosidases and the most thermostable endoglucanase known so far, according to our knowledge (Fig. 10).

The structure of TMDG_GH12‐2 and the structural model of TMDG_GH12‐1 both exhibit beta‐jelly roll folds, a characteristic of GH12 family enzymes (https://www.cazy.org/GH12.html). Although the sequences of TMDG_GH12 enzymes are only 18% identical, their tertiary structures are quite similar. However, they differ in some structural details which might shed light on peculiarities in their optima of activity, thermostability, and substrate specificity. First, the TMDG_GH12‐1 structure had slightly longer loops between beta‐strands (A1–A4, B3, B4, B7, and B8) and one (A1) of these loops even forms an additional rudimentary beta‐strand (Fig. 4). At the same time, TMDG_GH12‐1 possesses slightly shorter B1‐B2 strands than TMDG_GH12‐2. It should be noted that both disulfide bridges of TMDG_GH12‐1, absent in TMDG_GH12‐2, are located on the most extended loops. Second, in TMDG_GH12‐1, only 5 glutamates are present in the catalytic site compared to 7 in TMDG_GH12‐2, with glutamates E14 and E30 in TMDG_GH12‐2 substituted with D60 and V51 in TMDG_GH12‐1. Third, the distribution of the three tryptophan residues in the catalytic cavity differs. In TMDG_GH12‐1, they are shifted from the catalytic residues towards the non‐reducing end of the substrate compared to TMDG_GH12‐2 (Figs S7–S9). The extension of the substrate‐binding cavity enables additional hydrogen bonds and hence a more stable substrate‐enzyme complex, which may also contribute to the higher temperature optimum of TMDG_GH12‐1. Finally, these changes in the substrate‐binding cavity might also contribute to differences in the substrate specificity.

Despite the lack of the Ca^2+^ binding site in TMDG_GH12‐1, it is the much more thermostable protein. On the other hand, the presence of cysteines moieties only in TMDG_GH12‐1 suggests that disulfide bridges might make a greater contribution to stability compared to one Ca ^2+^‐binding site in TMDG_GH12‐2. The bridges might stabilize flexible loops connecting beta‐strands of the TMDG_GH12‐1 jelly roll fold. This is in agreement with studies on the influence of metal ions, chelating agents, and reducing agents on the activity of both TMDG_GH12 proteins. Divalent ions had no effect on the activity of TMDG_GH12‐1 and activated TMDG_GH12‐2, while EDTA had no effect on the activity of TMDG_GH12‐1 and inhibited TMDG_GH12‐2. Finally, DTT and β‐mercaptoethanol, known to be capable of reducing disulfide bonds, inhibited the TMDG_GH12‐1 enzyme. Surprisingly, these reducing agents also inhibited TMDG_GH12‐2, but the inhibition was less pronounced (Table 4). Therefore, long loops and the disulfide bridges contributed to the overall stability of the TMDG_GH12‐1 protein, making it more compact. Disulfide bridges are well known to promote protein stability [48, 49].

Molecular docking of cellohexaose into TMDG_GH12‐2 domain highlighted the importance of aromatic residues (W12, W34, W71, and W85 (W43, W49, W64, W120 in the TMDG_GH12‐1)) as well as the N32, N86, K83, E30, and E77 for ligand stabilization (F232, Y105, Y110 and K118, E60, E112, N122 for TMDG_GH12‐1). The hydrogen bond network that orients catalytic E147 and E240 has also been shown (E181, E279 in TMDG_GH12‐1 Table S5). This long substrate‐binding groove situated along one side of the protein, which binds several consecutive sugar residues, is a remarkable feature of cellulases (endoglucanases). The presence of many residues capable of hydrogen bonding is common for groove composition, as well as a few aromatic residue side chains, which are often found as hydrophobic platforms that form discrete sugar‐binding subsites.

Comparison of xylohexaose docking results for TMDG_GH12‐1 and TMDG_GH12‐2 domains did not allow us to identify the residues, that determine the capability of TMDG_GH12‐1 to hydrolyze xylan and arabinoxylan. This may be due to several factors: first, the hexameric ligand is the largest possible oligosaccharide for our docking experiment; and second, we used a simplified model of the oligosaccharide that lacks branching and other variations found in nature. Notably, the TMDG_GH12‐1 and TMDG_GH12‐2 domain proteins have accumulated a significant amount of structural differences at the catalytic crevice periphery, which we believe could be crucial for modulating specific enzymatic activities. Molecular dynamics simulations using longer oligosaccharide chains could provide further insights to support this hypothesis. It was also noticed for the 3AMM structure (cellulase 12A from *T. maritima* [33], Table S7) that each of the 6‐hydroxyl groups in the −2, −1, +1, +2 glycosyl residue forms at least one direct hydrogen bond to the protein. Two of these interactions in 3AMM are provided by R60, which has no structural homologs in both TMDG_GH12‐2 and TMDG_GH12‐1 implying that the 6‐hydroxyl groups of −2 and +1 glycosyl residues in TMDG_GH12 proteins are not participating in protein‐ligand stabilization (Fig. 5).

Alanine scanning of TMDG_GH12‐2 showed that almost all of the 22 substitutions reduced the activity and thermostability of the enzyme. It is most probably because alanine interferes with the proper stacking of surrounding residues, which leads to lowering the stability of the molecule [50]. However, three mutations – W130A, N86A, and D92A – resulted in a slight increase in *t*_½_ at 100 °C compared to the wild‐type (Fig. 6). At the same time, these substitutions reduced the activity (Fig. 6).

In accordance with the assumption that all the substituted residues were predicted to be important for enzyme stability and activity, they were conserved in the multiple sequence alignment of biochemically characterized GH12s (Fig. 7). For TMDG_GH12‐1, the only exception was W235 (consensus sequence coordinates, Fig. 7) which was replaced with glutamine (Q185 in TMDG_GH12‐1 coordinates). Given the exceptional thermostability of this enzyme, we hypothesized that this substitution might play an important role here. Indeed, the substitution Q185 → W reduced both the activity and thermostability of the enzyme: its half‐life at 90 °C decreased from 284.5 to 150 h and at 100 °C from 42 to 25 h (Fig. 8A,B). However, the opposite substitution in TMDG_GH12‐2 (W151 → Q in TMDG_GH12‐2 coordinates) also led to reduced activity and stability: the half‐life at 90 °C decreased from 19 to 12 h and at 100 °C from 30 to 20 min (Fig. 8C,D). These results imply that the W → Q replacement in this position is either effective only in the specific amino acid environment of TMDG_GH12‐1, or the positive effect of this mutation in TMDG_GH12‐2 was overcome by the overall negative effect on its structural stability, or it has no effect on thermal stability at all. Supporting the first option is the fact that, according to phylogenetic analysis, this replacement occurred quite recently: Q at this position was found only in TMDG_GH12‐1 and its two most closely related enzymes (Fig. 2, Fig. S1) from the hyperthermophilic crenarchaeon *I. aggregans*, from which the gene encoding TMDG_GH12‐1 was most probably horizontally transferred to strain 2319x1 or its nearest ancestor before being fused into the *mdg* gene. The third nearest relative, also from *I. aggregans*, has a W → E substitution at this site while all other GH12 proteins have a tryptophan residue there.

**Fig. 11:**
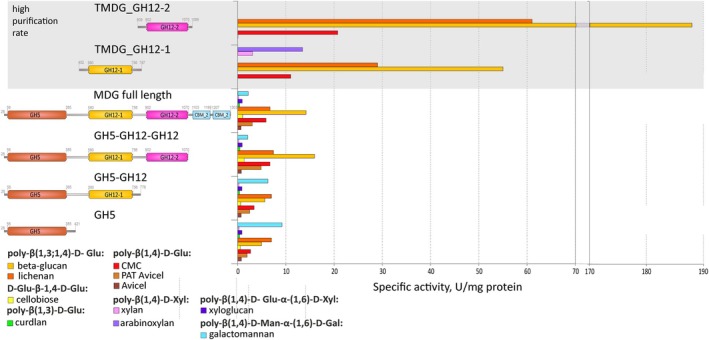
Specific activity of recombinant MDG, its truncated versions, and single domains. The results for individual GH12s were obtained during this work (all measurements were done in triplicates), the numbers for all other versions of the MDG were published by us earlier [30].

Both TMDG_GH12 domains were mostly active with barley β‐glucan. Their specific activity was 3–9 times higher than the complete MDG as well as its other truncated versions, including individual GH5 (Fig. 11). In turn, the variety of substrates these two TMDG_GH12 hydrolyze was lower. Lichenan and CMC were other substrates common to both GH12 enzymes, while TMDG_GH12‐1 was also active against arabinoxylan and xylan. The common feature between these substrates is the presence of beta‐1,4 glycosidic (glucosidic or xylosidic) linkages in its backbone, implying these bonds are the only targets of the studied GH12s. This fits well with the fact that both TMDG_GH12 did not hydrolyze curdlan and pachyman (beta‐1,3‐linked glucosides), polysaccharides with other beta‐1,4 bonds (glucomannan, mannan, galactan and chitosan) as well as alpha‐1,4‐glucosides (starch) and short tri‐ and disaccharides: raffinose, cellobiose, and trehalose. Their inability to hydrolyze xyloglucan, which is also a polysaccharide with a beta‐1,4 glucosidic backbone, is most probably due to the side chains interfering with the formation of the substrate‐enzyme complex. It should be noted that the MDG and its truncated versions were also barely active on this substrate. TMDG_GH12‐2 had a higher specific activity than TMDG_GH12‐1 on the common substrates; moreover, TMDG_GH12‐2 was one of the most active archaeal beta‐glucan and lichenan‐hydrolyzing enzymes (Fig. 11, Table S1 [28]). The specific activity of TMDG_GH12‐1 towards xylan (2.4 ± 0.25 U·mg^−1^) or arabinoxylan (13.5 ± 0.6 U·mg^−1^) is comparable to or higher than that of other archaeal GH12 glycosidases capable of hydrolyzing these polysaccharides: EglA from *P. furiosus* (0.045 and 0.048 U·mg^−1^ for xylans and arabinoxylans, respectively [23]), GH12 glycosidase from *Saccharolobus solfataricus* (4.6 U·mg^−1^ for beechwood xylan [25]) and Cel12E from *Thermococcus* enrichment culture (0.1–0.38 U·mg^−1^ [24]). Both TMDG_GH12 proteins had a higher affinity for mixed beta‐1,4, beta‐1,3 substrates (β‐glucan and lichenan) and lower affinity for CMC, which might be due to the origin (natural or artificial), molecular weight (lower or higher), viscosity (low or high) or primary structure (beta 1,4‐beta‐1,3 or beta‐1,4 only) of β‐glucan and lichenan or CMC, respectively. TMDG_GH12‐1 has a higher affinity for xylan (*K*_M_, 1.11 mg·mL^−1^) than for arabinoxylan (*K*_M_ of 2.28 mg·mL^−1^) implying better binding to the xylose‐consisting backbone without arabinose side chains.

During hydrolysis of all tested substrates, TMDG_GH12‐2 released low molecular weight oligosaccharides (C1–C4) while TMDG_GH12‐1 also released C5–C6 and higher molecular weight oligomers on beta‐glucan, lichenan, and xylan (Fig. S6). Formation of single glucose units as well as cellobiose (C2) is an indication that both enzymes possess beta‐glucosidase and/or cellodextrinase activities [20]. On the other hand, based on the facts that C1 and C2 were produced in low amounts despite rather high specific activities of both TMDG_GH12s, as well as that both enzymes hydrolyzed CMC—a specific substrate for endoglucanases—and both were unable to degrade C2‐C6 oligosaccharides (in contrast to MDG and its GH5 [30]), the assumption was made that both of the TMDG_GH12 enzymes are endoglucanases with minor exoglucanase activity. Accordingly, the earlier made predictions [30] were correct for TMDG_GH12‐1 but were not for TMDG_GH12‐2, for which the exoglucanase activity was proposed. A prediction of endoxylanase activity made earlier for TMDG_GH12‐1 was also confirmed; however, based on the analysis of the products of xylan hydrolysis, a broader xylanase activity could be proposed for this enzyme.

## Conclusions

Despite being encoded by a single *mdg* gene, the two GH12 domains described here are evolutionarily distant and exhibit distinct substrate specificities, temperature and pH optima, resistance to detergents, and thermostability. So far, as we know, the TMDG_GH12‐1 domain is one of the most thermostable glycosidases and the most thermostable cellulase (endoglucanase) known to date, whereas the TMDG_GH12‐2 is so far the only known glycoside hydrolase from hyperthermophilic archaea optimally active at high pH. Both glycosidases possessed remarkably high half‐life times at 90 °C and 100 °C, making them highly attractive for bioindustry. Exceptional thermostability of TMDG_GH12‐1 is not completely understood, but two disulphide bridges, the W → Q mutation in the active site, and the longer substrate‐binding cavity are likely to be involved. Obtaining crystals for TMDG_GH12‐1 followed by site‐directed mutagenesis studies of this enzyme might provide more insights into its thermal stability.

Multidomain glycosidase (MDG) from *Thermococcus* sp. contains three glycosidase domains—one GH5 and two GH12—all of which were characterized as catalytically active enzymes. All three are endoglucanases varying in side activities as well as in thermostability and kinetic properties. The presence of three catalytic domains with different evolutionary histories in one enzyme might contribute to the ecological plasticity of the *Thermococcus* representative inhabiting tidal zones with drastic changes in environmental conditions as temperature, pH, salinity, and amounts of available substrates.

## Materials and methods

### Phylogenetic analysis

The domain context of the proteins, containing GH12 domains as well as coordinates of GH12 domains, was predicted with HMMscan (https://www.ebi.ac.uk/Tools/hmmer/search/hmmscan, [51]) and the Pfam database.

For phylogenetic analysis, the sequences of GH12 domain‐containing polypeptides with protein existence at “evidence at protein level”, “evidence at transcript level” and “inferred from homology” were retrieved from the UniProtKB. The protein sequences with “evidence at protein level” were marked together with sequences of other biochemically characterized proteins for which this property is not indicated in the UniProtKB. Additionally we verified the sequences of all GH12 proteins, available in Pdb and all of them were already found using the indicated above pipeline with one exception – UniProt ID: A0A0T6ZV48 which is indicated in this database as “obsolete”. Further, one short truncated sequence (UniProt ID: P84196, 36 amino acids), was deleted from the database. Also MDG itself was replaced with two individual GH12 domain sequences from this work. Resulted database was checked on the presence of sequences with more than one GH12 domains and one such sequence (UniProt ID: A0A2T9WRP5) was split on three GH12‐containing sequences, according to the number of GH12 domains. Finally, the database was clustered using 70% sequence identity threshold using local CDhit [52] and 149 sequences representing unique clusters were used for phylogenetic analysis.

The sequences were aligned in psi‐coffee, m‐coffee, espresso (using pdb 4NPR, pdb 3O7O and pdb 3WQ7 structures), clustal_omega, muscle, and mafft (L‐INS‐i). All the alignments were then weighted and filtered using tcs [53]. The resulted tcs alignments appeared to be highly similar with scores from 531 (muscle) to 569 (psi‐coffee) and the lengths from 156 (muscle) to 237 (espresso).

All six tcs alignments were taken for phylogeny inferring performed in mega x [54] using the maximum likelihood method. Bootstrap of 100 replications was used to test the confidence of the phylogenies. A comparison of the trees using https://beta.phylo.io/viewer/# showed nearly identical topology. A consensus tree was constructed from mafft and clustal_omega trees using iphyloc [55].

### Construction and cloning of the individual TMDG_GH12‐1 and TMDG_GH12‐2 proteins

Cloning of ORFs encoding TMDG_GH12‐1 (1005 bp) and TMDG_GH12‐2 (840 bp) was performed using the aLICator Ligation Independent Cloning and Expression System kit (#K1251; Thermo Scientific, Vilnius, Lithuania). The genes were amplified according to Innis *et al*. [56] using the *de novo* designed primers (Table S8) and the full *mdg* gene cloned in the pET24a vector [30] as the DNA template.

All primers include vector‐specific sequences (underlined in the Table S8) complementary to linear vector pLATE 51, which contained an N‐terminal His_6_‐tag and an enterokinase cleavage site (DDDDK^).

PCR products were purified with the Cleanup Standard Kit (#BC022; Evrogen, Moscow, Russia). The ligation independent cloning (LIC) reaction was performed for 5 min at 25 °C, upon which the vector with the insert was directly transformed into *E. coli* BL21 (DE3) competent cells. The efficiency of the LIC reaction was tested using the same approach as outlined in [57].

### Gene expression and enzyme purification

For the expression of recombinant TMDG_GH12‐1 and TMDG_GH12‐2 domains, two strains of *E. coli* BL21 (DE3) pLATE51::*tmdg_gh12‐1* and *E. coli* BL21 (DE3) pLATE51::*tmdg_gh12‐2* were grown on LB medium with ampicillin, 100 μg·mL^−1^ (38 and 10 L respectively) at 37 °C for 5 h to an optical density (λ = 600 nm) of 0.5. Gene expression was induced by the addition of 0.5 mm isopropyl β‐d‐1‐thiogalactopyranoside (IPTG). After 16 h of incubation at 20 °C, cells were harvested by centrifugation (9000 ***g***, 4 °C, 20 min) and resuspended in 50 mm Tris/HCl buffer pH 8.0 with 150 mm NaCl and 25 mm imidazole with the addition of 0.1% (v/v) protease inhibitor cocktail (#P8849; Sigma‐Aldrich, Saint Louis, MO, USA). Cells were disintegrated by sonication using SoniPrep (MPBio, Santa Ana, CA, USA) at 4 °C. Disrupted cells were centrifuged (13 400 ***g***, 4 °C, 30 min). Each of the supernatants with TMDG_GH12 polypeptides was purified in three steps—(i) affinity, (ii) anion‐exchange, and (iii) size exclusion fast protein liquid chromatography (FPLC, Äkta Start, Cytiva, Marlborough, MA, USA). The first purification step of metal affinity chromatography was performed on HP 1 mL HisTrap column Ni‐NTA resin (Cytiva). The column was equilibrated with Ni‐A buffer (50 mm Tris/HCl pH 7.5, 0.5 M NaCl, 25 mm imidazole). The supernatant was adjusted and washed with 15 column volumes (CV) of a Ni‐A buffer. Protein elution from the column was performed by a linear gradient of imidazole from 25 to 500 mm using Ni‐B buffer (50 mm Tris/HCl pH 7.5, 0.5 m NaCl, 500 mm imidazole); the gradient volume was 20 CV. Then, fractions containing protein were combined together and transferred to 15‐mL Amicon centrifugal filtration units with a molecular weight cut‐off (MWCO) of 30 kDa, diluted with 50 mm Tris/HCl (pH 7.8) and centrifuged for 10–15 min at 3000 ***g***. Diethylaminoethanol (DEAE) Sephadex A25 resin and a 10 mL C10/10 column (Cytiva) were used at the ion exchange chromatography step. The column was equilibrated with Ion‐A buffer (50 mm Tris/HCl pH 7.5). The supernatant was adjusted and washed with 2 CV of a Ion‐A buffer. Protein elution from the column was performed by a linear gradient of NaCl from 0 mm to 1 m using Ion‐B buffer (50 mm Tris/HCl pH 7.5, 1 m NaCl); the gradient volume was 15 CV. Size exclusion chromatography (gel filtration) was employed as the final step of purification. In this regard, the sample was concentrated and desalted by 15‐mL Amicon centrifugal filtration units with MWCO of 30 kDa (15 min at 3000 ***g***), adjusted on a C16/100 column with Sephadex G‐100 per grade column (Cytiva) equilibrated with GF‐buffer (50 mm Tris/HCl pH 7.8, 0.1 m KCl) and isocratically eluted with 4 column volumes. The resulting sample was concentrated by Amicon ultra MWCO 3 kDa in 50 mm Tris/HCl (pH 7.8) buffer. Protein standards for gel filtration were Gel Filtration Markers Kit for Protein Molecular Weights 6500–66 000 Da, Merck KGaA, Darmstadt, Germany.

To purify each mutant protein, 0.6 L of recombinant *E. coli* culture was grown followed by the gene expression and cells lysis, all of which were done as described above. After centrifugation, the supernatants were purified in one step using only metal affinity chromatography (Gravity Flow HP 1 mL Ni‐Sepharose column, Cytiva, 50–500 mm imidazole step gradient), followed by desalting on Desalting Column PD 10, G25, Cytiva. For better comparison between mutant and wild‐type proteins, the respective wild‐type proteins were purified in parallel to the mutant counterparts and to the same extend—i.e., one step of metal affinity chromatography, followed by desalting with PD 10 columns.

The presence of the target proteins was confirmed by electrophoresis in a polyacrylamide gel [58]. As a protein standard Protein ladder #26630, Thermo Scientific, Vilnius, Lithuania, was used. Protein concentrations were measured using the Qubit Protein Assay Kit, Thermo Scientific, Rockford, IL, USA.

### Biochemical characterization of TMDG_GH12 domains

Glycoside hydrolase activities were measured using the DNSA assay [59]. Reactions were initiated upon adding 50 μL cell extract or purified enzyme preparations to preincubated 950 μL 50 mm MES buffer (pH 5.3) for TMDG_GH12‐1 or 50 mm Tris/HCl buffer (pH 9.0) for TMDG_GH12‐2, containing 0.1% substrate (see below) followed by incubation at various temperatures and various times (see the results) in a Thermo Shaker TS‐100C (Biosan, Riga, Latvia). The same reaction mixtures without protein were used as the control experiments. Aliquots of 300 μL of the reaction mixture were taken at the time points 0, 10, 20 min for TMDG_GH12‐1 and 0, 3, 5 min for TMDG_GH12‐2. In each aliquot, the fermentative reaction was stopped by adding the 3,5‐dinitrosalicylic acid (DNS) reagent (1 : 1, v : v) followed by cooling down to room temperature. Afterwards, the mixtures were incubated for 15 min at 98 °C, cooled down again to room temperature, and the absorbance at 575 nm was measured with a spectrophotometer SPECTROstar Nano (BMG Labtech, Ortenberg, Germany). All measurements were made in triplicate. Temperature dependence was measured at pH values optimal for each domain (pH 5.0 for TMDG_GH12‐1 and pH 9.0 for TMDG_GH12‐2) with barley beta‐glucan as a substrate, using a Thermo Shaker TS‐100C (Biosan) for the temperatures 30–100 °C or a silicone oil bath IB 20pro (IKA‐Werke, Staufen im Breisgau, Germany) at the temperatures 100–130 °C. In the latter case, the measurements were carried out in 15‐mL Hungate tubes with butyl rubber stoppers and screw caps to prevent evaporation of the reaction mixture. Each tube contained a 7 mL of the reaction mixture. The headspace was filled with 100% air. Prior to the injection of 350 μL enzyme solution into each tube with a 1 mL syringe, the reaction mixture was preincubated at the respective temperature for 20 min. Reactions were stopped by the addition of DNS and cooling, followed by the measurement of reducing sugars, as described above.

The influence of pH on the activity of the TMDG_GH12 glycosidases was analyzed in the pH range 3.0–11.5 in 100 mm acetate buffer (pH 3.0–5.0), 50 mm MES buffer (pH 5–6), 50 mm MOPS (pH 6.0–7.0), 50 mm Tris/HCl (pH 7.0–9.5) and 50 mm CAPS (pH 9.5–11.5) at 100 °C for TMDG_GH12‐1 or 90 °C for TMDG_GH12‐2.

The following polysaccharides were tested as the substrates: barley β‐glucan, pachyman, lichenan, glucomannan (konjac), mannan (from Ivory nut), xyloglucan (from tamarind), xylan (from beechwood), arabinoxylan (oat spelt), galactan (from potato) (all from Megazyme, Wicklow, Ireland), chitin, starch (Merck, Darmstadt, Germany), carboxymethyl cellulose (CMC), Avicel (both from Fluka, Merck, Darmstadt, Germany), alpha‐cellulose (Sigma), PASC (phosphoric acid swollen cellulose, prepared according to [60]), raffinose (DiaM, Moscow, Russia), cellobiose (Acros Organics, Geel, Belgium), and trehalose (DiaM) (each 1% w/v). The substrate specificity measurements were carried out under optimal conditions for each domain. In addition, AZO‐dyed substrates like AZO‐avicel, AZO‐galactomannan, AZO‐galactan, AZO‐xylan, and AZO‐xyloglucan were tested, and their hydrolysis was measured according to the recommendations of the manufacturer (Megazyme). Specific activity is determined by reference to the standard curve to convert absorbance to milliUnits of activity per assay according to the protocol for each AZO‐dyed substrate.

One unit of enzymatic activity (U) is the amount of substrate hydrolyzed per minute of the reaction which was calculated as the amount of released sugar (xylose or glucose in μmols) per minute. Specific activity is an enzymatic activity divided by total protein concentration (U·mg^−1^ of protein) which is, in the case of homogeniuos enzyme preparations, equal to the amount of the respective enzyme in the reaction mixture (*C*_prot_).

Determining the GH12 domains kinetic parameters (*K*_M_ and *V*_max_) was performed with different concentrations 0.25, 0.5, 1, 1.5, 2, 2.5, 3, 4, 5, 6, 7, 9, 10, 15 mg·mL^−1^ of barley β‐glucan, CMC, lichenan, xylan, arabinoxylan, AZO‐CMC, and AZO‐xylan. To calculate *K*_M_ (mg·mL^−1^ of substrate) and *V*_max_ (μmol of releasing sugars per minute, divided by a milligram of protein) values (Figs S4 and S5), the data was plotted using the Lineweaver–Burk or Hanes‐Woolf coordinates. Catalytic specificity constant (*k*_cat_, 1/s) was calculated using the equation: *k*_cat_ = *V*_max_/([Et] × 60), were [Et] – total enzyme concentration ([Et] = [*C*_prot_]/MW), *C*_prot_ – amount of enzyme, present in the reaction mixture in μg, MW – molecular weight of the protein in Da. The multiplication by 60 was made as a conversion from minutes to seconds since the initial *V*_max_ values were in μmol of releasing sugars per minute.

The influence of metal ions, detergents, chelating, denaturing and reducing agents was evaluated by incubation the reaction mixtures, described above contained 5 mm of each of the following agents: AgNO_3_, CaCl_2_, CuCl_2_, FeCl_2_, FeCl_3_, KCl, MgCl_2_, MnCl_2_, NaCl, ZnCl_2_, ethylenediaminetetraacetic acid (EDTA), dithiothreitol (DTT), Tween 80, β‐mercaptoethanol, urea, and sodium dodecyl sulfate (SDS). The enzymatic activities were measured using DNSA assay as it described above.

Thermostability of the TMDG_GH12 glycosidases expressed as the enzymes' half‐life time (*t*_½_) was revealed by preincubation of both enzymes at optimal pH at 100 °C (for 4 days) and 90 °C (for 17 days) followed by activity measurements at the optimal for each enzyme conditions at different preincubation time points: 0, 1, 2, 4.5, 24, 49, 97, 135, 184, 302, 374 h.

Hydrolysis products of barley β‐glucan, lichenan, CMC, and xylan degradation were determined by thin layer chromatography (TLC) the same way as it was described by Zayulina *et al*. [57].

For activity and thermostability determination of the mutant and wild‐type enzymes, the standard activity assay was performed with 0.3% substrate in optimal conditions: 50 mm MES buffer (pH 5.3) and 100 °C for TMDG_GH12‐1, or 50 mm Tris/HCl buffer (pH 9.0) and 90 °C for TMDG_GH12‐2. Since both mutant and wild‐type enzymes passed one‐step purification, in this case, the catalytic parameters of the latter were slightly different from the enzyme preparations purified to homogeneity using three‐step purification procedure.

### Protein crystallization, data collection, structure determination, and refinement

TMDG_GH12‐2 was crystallized at room temperature using the sitting‐drop vapor diffusion method (protein : precipitant ratio 1 : 1; protein concentration 10 mg·mL^−1^; reservoir solution 2 m ammonium sulfate, 2% (v/v) PEG400 and 0.1 m HEPES‐K, pH 7.5) The crystal was cryoprotected by transferring it into paratone oil, then flash cooled in liquid nitrogen. Diffraction data for the TMDG_GH12‐2 crystal was collected at 100 K at a Rigaku home source Micromax‐007 with R‐AXIS IV++ detector. Diffraction data was processed using hkl3000 [61]. The structure was solved by molecular replacement using phenix.phaser [62] and the structure of the GH12 endocellulase from *P. furiosus* as a model (PDB code  3VGI [63]). Model building and refinement were performed using phenix.refine and coot [64]. TLS parameterization was utilized for refinement, and B‐factors were refined as isotropic. Structure geometry and validation were performed using the Phenix Molprobity tools. Data collection and refinement statistics for this structure are summarized in Table S4. The crystal structure of TMDG_GH12‐2 is available from PDB under accession number 7S8K.

### Molecular docking

TMDG_GH12‐1 domain structure was predicted with AlphaFold2 using the ColabFold platform [31, 65]. The online version of AlphaFold2.ipynb v1.2.0 was running on the TMDG_GH12‐1 amino acid sequence with template_mode turned off. Among the five resulting models, the model with the highest pLDDT score was chosen for further analysis.

Docking was performed using the Rosetta GlycanDock package [66] which requires an initial protein‐ligand complex as an input. The initial structure was gained similar to Godoy *et al*. [67]. Binding site residues of the TMDG_GH12‐1 and TMDG_GH12‐2 structures were aligned to the cellotetraose‐containing cellulase 12A from *T. maritima* (PDB ID: 3AMM), then cellotetraose coordinates from the *T. maritima* complex were copied into the structures of TMDG_GH12‐1 and TMDG_GH12‐2 domains, and two additional glucosyl residues were added to the −3 and −4 subsites. For xylohexaose‐containing complexes, glycosyl residues were manually modified. Resulting complexes were prepared using CHARMM‐GUI Glycan Reader&Modeller [68] and then prepacked using the following command: ./GlycanDock.linuxgccrelease –include_sugars –alternate_3_letter_codes pdb_sugar –auto_detect_glycan_connections –in:file:s rosetta_input.pdb –nstruct 1 –ex1 –ex2 –ex3 –ex4 –ex1aro –ex2aro –carbohydrates:glycan_dock:prepack_only true –docking:partners A_B –out:pdb_gz.

Docking procedure was performed with the following flags: GlycanDock.linuxgccrelease –include_sugars –maintain_links –in:file:s rosetta_input_0001.pdb.gz –nstruct 50 –n_cycles 10 –ex1 –ex2 –docking:partners A_B –out:pdb_gz.

### Site‐directed mutagenesis and cross‐mutagenesis

Site‐directed mutagenesis was performed using the QuikChange II Site‐directed Mutagenesis kit (Agilent, Santa Clara, CA, USA) according to the manufacturer's instructions. Twenty‐two conserved and semi‐conserved residues of the TMDG_GH12‐2 domain located in the putative functional positions (W12, D18, Y28, E30, N32, W34, S70, E77, N82, K83, W85, N86, N87, N88, D92, W130, E147, M149, W151, E233, E236, and E240) were mutated to alanine. For the determination of the functions of the non‐conservative residue in the TMDG_GH12‐1 domain, the Q185 was changed to tryptophan (W); alternatively, in the TMDG_GH12‐2 domain, the W151 was changed to glutamine (Q). The TMDG_GH12‐2 open reading frame cloned on the pLATE51 expression vector was used as a template. Primers carrying specific mutations were designed using the QuikChange Primer Design Program www.agilent.com/genomics/qcpd. The primer sequences are listed in Table S9. The standard PCR mixture contained 50–100 ng of template DNA and 150–250 ng of each primer. The methylated plasmid was digested with *Dpn*I. Two microliter of each reaction mixture were used to transform competent *E. coli* XL1‐Blue Supercompetent Cells (Agilent). Ampicillin‐resistant colonies were selected from LB agar plates, and the specific alanine changes were verified by DNA sequencing. The recombinant plasmids with confirmed mutations were transformed into the *E. coli* BL21 (DE3) strain for overexpression.

## Conflict of interest

The authors declare no conflict of interest.

## Author contributions

IVK designed research; KSZ, AAK, PS, AFY, PNG, BS, TES, IVK planned experiments; KSZ, ENF, CS, AAK, ANK, PS, TS, TES performed experiments; KSZ, IVK, TES wrote the manuscript; KSZ, ENF, AFY, PNG, BS, TES, IVK discussed the results and contributed to the final version of the manuscript.

## Peer review

The peer review history for this article is available at https://www.webofscience.com/api/gateway/wos/peer‐review/10.1111/febs.70095.

## Supporting information


**Fig. S1.** Detailed maximum likelihood phylogenetic tree of GH12 glycosidases.
**Fig. S2.** SDS/PAGE of TMDG_GH12 domains.
**Fig. S3.** Size exclusion chromatography of GH12 domains.
**Fig. S4.** Substrate specificity of TMDG_GH12‐1 protein.
**Fig. S5.** Substrate specificity of TMDG_GH12‐2 protein.
**Fig. S6.** TLC of sugars, released during polysaccharide hydrolysis by TMDG_GH12 proteins.
**Fig. S7.** Active site of the TMDG_GH12‐2 domain: close‐up view.
**Fig. S8.** Active site of the TMDG_GH12‐1 domain: close‐up view.
**Fig. S9.** Different location of tryptophan moities in the reaction cavities of TMDG_GH12‐1 and TMDG_GH12‐2.
**Fig. S10.** Cellohexaose docking into the active sites of TMDG_GH12‐1 and TMDG_GH12‐2 domains.
**Table S1.** Characteristics of published archaeal cellulases.
**Table S2.** Calculated characteristics of GH12 proteins.
**Table S3.** Determination of the mass of GH12 domains after gel filtration on Sephadex G‐100.
**Table S4.** X‐ray crystallographic statistics for the structure of the MDG TMDG_GH12‐2 domain.
**Table S5.** Molecular docking.
**Table S6.** Effect of mutations on glycosidase activity of purified TMDG_GH12‐2.
**Table S7.** Characteristics of representatives of GH12 family with optimal activity higher than 45 °C.
**Table S8.** Primers designed for *tmdg_gh12‐1* and *tmdg_gh12‐2* gene fragments.
**Table S9.** Primer sequences (5′ to 3′) for alanine scanning and cross‐mutagenesis.
**Table S10.** TMDG_GH12 sequences.

## Data Availability

The data that support the findings of this study are available in the main text and Supporting Information. TMDG_GH12 sequences and TMDG_GH12‐2 mutant sequences can be found in Table S10 and the GenBank (accession numbers PV357969 ‐ PV357994). The crystal structure of TMDG_GH12‐2 is available from PDB under accession number 7S8K (https://www.rcsb.org/structure/7S8K). TMDG_GH12 docking poses are available in Zenodo (https://doi.org/10.5281/zenodo.13509639).
