# Supplementary material for: Structural and functional insights into extreme thermal stability and activity of two GH12 domains of a multidomain glycosidase from a hyperthermophilic euryarchaeon
Source: FEBS J. 2025 Apr 21;292(14):3771–94. doi: 10.1111/febs.70095 (PMC12265866; doi:10.1111/febs.70095)
Supplement: Supplementary file 1 — Fig. S1. Detailed maximum likelihood phylogenetic tree of GH12 glycosidases. Fig. S2. SDS/PAGE of TMDG_GH12 domains. Fig. S3. Size exclusion chromatography of GH12 domains. Fig. S4. Substrate specificity of TMDG_GH12‐1 protein. Fig. S5. Substrate specificity of TMDG_GH12‐2 protein. Fig. S6. TLC of sugars, released during polysaccharide hydrolysis by TMDG_GH12 proteins. Fig. S7. Active site of the TMDG_GH12‐2 domain: close‐up view. Fig. S8. Active site of the TMDG_GH12‐1 domain: close‐up view. Fig. S9. Different location of tryptophan moities in the reaction cavities of TMDG_GH12‐1 and TMDG_GH12‐2. Fig. S10. Cellohexaose docking into the active sites of TMDG_GH12‐1 and TMDG_GH12‐2 domains. Table S1. Characteristics of published archaeal cellulases. Table S2. Calculated characteristics of GH12 proteins. Table S3. Determination of the mass of GH12 domains after gel filtration on Sephadex G‐100. Table S4. X‐ray crystallographic statistics for the structure of the MDG TMDG_GH12‐2 domain. Table S5. Molecular docking. Table S6. Effect of mutations on glycosidase activity of purified TMDG_GH12‐2. Table S7. Characteristics of representatives of GH12 family with optimal activity higher than 45 °C. Table S8. Primers designed for tmdg_gh12‐1 and tmdg_gh12‐2 gene fragments. Table S9. Primer sequences (5′ to 3′) for alanine scanning and cross‐mutagenesis. Table S10. TMDG_GH12 sequences. [file FEBS-292-3771-s001.pdf]

Supplementary materials

Structural and functional insights into extreme thermal stability and activity of two GH12 domains of a multidomain glycosidase from a hyperthermophilic euryarchaeon.

Kseniya S. Zayulina *[1], Evgenii N. Frolov [1], Christina Stracke[2], Alexandra A. Klyukina[1], Anna N. Khusnutdinova [3], Peter Stogios [4], Tatiana Skarina[4], Alexander F. Yakunin [3,4], Peter N. Golyshin [3], Bettina Siebers [2], Tatiana E. Shugaeva [5,6#], Ilya V. Kublanov *[1,7#]

[1] Winogradsky Institute of Microbiology, Federal Research Center of Biotechnology, Russian Academy of Sciences, Moscow, Russia

[2]Molecular Enzyme Technology and Biochemistry, Environmental Microbiology and Biotechnology (EMB), Centre for Water and Environmental Research (CWE), Faculty of Chemistry, University of Duisburg-Essen, Universitätsstr. 5, 45117 Essen, Germany.

[3] Centre for Environmental Biotechnology, School of Environmental and Natural Sciences, Bangor University, Bangor LL57 2UW, UK

[4] Department of Chemical Engineering and Applied Chemistry, University of Toronto, Toronto, Canada

[5] Department of Bioengineering and Bioinformatics, Moscow State University, Moscow, Russia

[6#] Science for Life Laboratory, Department of Applied Physics, KTH Royal Institute of Technology, 12121 Solna, Stockholm, Stockholm County 114 28, Sweden

[7#] Institute of Environmental Sciences, Hebrew University of Jerusalem, Rehovot 7610001, Israel

*Corresponding: k.zayulina@fbras.ru, kublanov.ilya@gmail.com

[#] Current affiliation

Table S1. Characteristics of published archaeal cellulases.

Protein id	Sequence id	Uniprot id	GH family	taxon	Type of enzyme	3D structure	T opt °C	pH opt	Thermostability, half-life	Substrate spec	Reference
EglA	NP_578583.1	Q9V2T0	GH12	*Pyrococcus furiosus*	recombinant	**3VGI[A]**	100	6	40 h, 95 °C	CMC, beta-glucan, lichenan, xylan	[1]
cel12E	A0A0F7YYA5		GH12	*Thermococcus enrich*	recombinant		92	5.5	2 h at 92 °C	CMC, beta-glucan, lichenan, PASC, HEC, xyloglucan, arabinoxylan, glucomannan	[2]
CelA2	SSO1949	Q97X08	GH12	*Saccharolobus solfataricus P2*	recombinant		80	1.8	8 h	CMC, cellooligosaccharides	[3]
CelA1 (CelB)	SSO1354	Q97YG7	GH12	*Saccharolobus solfataricus P3*	native		95	3.5	53 min	CMC, Beechwood xylan, Oat spelt xylan, Birchwood xylan, Arabinan, Debranched arabinan	[4]
SsGluC	LT221867	A0A8F5BPU9	GH12	*Saccharolobus shibatae*	recombinant		95-100	3.0-5.0	30 min - 30% at 90 °C, 2 h - 80% at 85 °C	CMC, beta-glucan, lichenan, xylan	[5]
TCel6	ABW02444.1	A8M9X3	GH12	*Caldivirga maquilingensis IC-167*	recombinant		85	5.5	2 h retained 26% at 60 °C	CMC, AMC, filter paper, MCC (cotton linters)	[6]
Vul_cel5A, cel5A	AZP54316.1	A0A3Q9CZQ1	GH5	*Hyperthermophilic archaeal enrichment*	recombinant		115	6	46 min, 100 °C	CMC, beta-glucan, lichenan, laminarin, glucomannan	[7]
EGPh;EglB; TCel3;PH1171	AAQ31833.1	O58925	GH5	*Pyrococcus horikoshii OT3*	recombinant	**2ZUM[A]**	85	5.6	80%, 3 h 90.7 °C	CMC, beta-glucan, MCC	[8]
GH5_Pool2	QFQ13828.1	A0A5P8EA52	GH5	*From hot spring*	recombinant		85	5.5	4 h at 90 °C	mannopentaose, 4NP-β-Man, and 4NP-β-Glc	[9]
EBI-244	AEB53062.1	F6M085	GH5	*Desulfurococcaceae archaeon EBI-244*	recombinant		109	5.5	4.5 h, 100 °C	CMC, AMC, filter paper, MCC	[10]
Lam16	AAC25554.2	E7FHE7	GH16	*Pyrococcus furiosus DSM 3638*	recombinant	**2VY0 [A,B]**	95	3	nd	laminarin	[11]
EBI-244	jf509452	F6M085	new*	*uncultured Ignisphaera*	recombinant		109	6.8	nd	CMC, beta-glucan, lichenan, Avicel	[10]
MDG	ALV63957.1	A0A0U3SGP7_9EURY	Multidomain / GH5**	*Thermococcus* sp.2319x1	recombinant		60 / 90	8.5 / 5.5	nd	CMC, amorphous cellulose, CE-cellulose, β-glucan, lichenan, xyloglucan, Avicel, xylan (beech, birch), locust bean gum, cellooligosaccharides (cellotriose to cellohexaose), cellobiose	[12]

* Belongs to GH A-Clan

** MDG is a multidomain enzyme consisting of GH5-GH12-GH12-CBM-CBM domains. The entire MDG and its GH5 domain were characterized

Table S2. Calculated characteristics of GH12 proteins.

	T1/2 (100)	T1/2 (90)	domains	size, kDa	aa*	IVYWREL aa content	Cysteins	Solubility* *	pI	Aliphatic aa: G,A,V,L,I (SeqManipSuite)	Aromatic aas: F,W,Y	Sulphur-containing aas: C,M	Basic aa: K,R,H	Acidic aa: B,D,E,N,Q,Z (Z=Glu/Gln)	Aliphati c hydroxy aa: l S,T
TMDG_GH12-1	45	288	GH12	37.1	335	0.36	4	0.732	4.55	34.33	11.94	3.88	6.87	20.3	16.42
TMDG_GH12-2	0,6	19	GH12	31.3	280	0.42	0	0.815	4.43	36.43	13.57	0.71	6.79	21.79	12.5
GH1 b-gal (celB) P.fur Q51723	85	nd	GH1-GH1-GH1***	54.6	472	0.43	1	0.902	5.42	35.17	14.62	2.97	14.83	20.34	6.99
GH12 (eglA) P.fur Q9V2T0	nd	40 (95)	GH12	36	319	0.42	0	0.89	4.9	35.11	12.23	0.94	8.78	19.44	16.61

* aa, - aminoacids

** Calculated in Soluprot (https://loschmidt.chemi.muni.cz/soluprot/)

*** It is uncertain if the whole protein was characterised

Table S3. Determination of the mass of GH12 domains after gel filtration on Sephadex G-100.

**Standard Protein**	**Mr, kDa**	**Ve, ml**	**Ve/V0**	**Calculated Mr, kDa (*in silico* predicted Mr)**
Aprotinin	6.5	170	2.50	
Cytochrome c	12.4	150.5	2.21	
Carbonic Anhydrase	29	132.5	1.95	
Albumine	66	97	1.43	
**TMDG_GH12-1**		120	1.76	**43.28 (34.8)**
**TMDG_GH12-2**		140	2.06	**26.36 (31.3)**

Ve- elution volume, V0 – void volume of the column (68 ml).

Table S4. X-ray crystallographic statistics for the structure of the MDG TMDG_GH12-2 domain.

**PDB code**	7S8K
**Data collection**	
**Space group**	C2
**Unit cell**	
**a, b, c (Å)**	137.35, 55.98, 91.91
**α, β, γ, (°)**	90, 126.56, 90
**Resolution, Å**	30.00 – 2.55
**Rmergea**	0.101 (0.509)*
**Rpim**	0.064 (0.382)
**CC1/2**	0.991 (0.651)
**I / σ(I)**	8.7 (1.1)
**Completeness, %**	99.5 (93.1)
**Redundancy**	3.4 (2.3)
**Refinement**	
**Resolution, Å**	28.74 – 2.55
**No. unique reflections:**	
**working, test**	18341, 918
**R-factor/free R-factorb**	18.1/22.1 (29.2/32.2)
**No. refined atoms, molecules**	
**Protein**	4405, 2
**Solvent**	72
**Water**	187
**B-factors**	
**Protein**	42.3
**Solvent**	55.8
**Water**	43.5
**r.m.s.d.**	
**Bond lengths, Å**	0.005
**Bond angles, °**	0.825

*values in brackets refer to highest resolution shells.

[a]$R_{merge} = \Sigma_{hkl}\Sigma_j|I_{hkl,j} - \langle I_{hkl}\rangle|/\Sigma_{hkl}\Sigma_j I_{hk,j}$, where $I_{hkl,j}$ and $\langle I_{hkl}\rangle$ are the jth and mean measurement of the intensity of reflection j.

[b]$R_{pim} = \Sigma_{hkl}\sqrt{(n/n-1)}\ \Sigma^{n}_{j=1}|I_{hkl,j} - \langle I_{hkl}\rangle|/\Sigma_{hkl}\Sigma_j I_{hk,j}$

[c] value refers to highest resolution shell.

[d]$R = \Sigma|F_p^{obs} - F_p^{calc}|/\Sigma F_p^{obs}$, where F_p^{obs} and F_p^{calc} are the observed and calculated structure factor amplitudes, respectively.

ND = not determined.

Table S5. Molecular docking.

Residues containing at least one atom located less than 4Å away from cellohexaose and xylohexaose for TMDG_GH12-1 and TMDG_GH12-2. Distances are measured for the pose TMDG_GH12-1-rosetta-prep_0001_0029 and TMDG_GH12-2-rosetta-prep_0001_0046 that has the lowest energy according to docking results.

TMDG_GH12-1		*TMDG_GH12-2*	
**Cellohexaose**	**Xylohexaose**	**Cellohexaose**	**Xylohexaose**
49TRP	43TRP	12TRP	12TRP
62ASN	49TRP	32ASN	14GLU
64TRP	51VAL	34TRP	32ASN
71ARG	62ASN	69GLY	34TRP
105TYR	64TRP	70SER	66LYS
106ASN	105TYR	71TRP	70SER
107THR	107THR	72VAL	71TRP
110TYR	110TYR	75TYR	72VAL
118LYS	112GLU	83LYS	75TYR
120TRP	118LYS	85TRP	83LYS
121GLY	120TRP	86ASN	85TRP
162GLU	122ASN	128GLU	86ASN
164TRP	162GLU	130TRP	87ASN
181GLU	164TRP	147GLU	88ASN
192TYR	181GLU	149MET	124ASN
193PRO	183MET	151TRP	128GLU
194ALA	192TYR	157LEU	130TRP
195GLY	194ALA	158GLN	147GLU
196TYR	224VAL	159PRO	149MET
197ASP	230TRP	160ALA	151TRP
224VAL	232PHE	161GLY	158GLN
226ALA	279GLU	189ILE	159PRO
230TRP	281TYR	191TRP	160ALA
232PHE		193TYR	161GLY
279GLU		236GLU	189ILE
281TYR		240GLU	191TRP
			193TYR
			240GLU

Table S6. Effect of mutations on glycosidase activity of purified TMDG_GH12-2.

Site of substitution	Mutation	Assumed role	Effect on relative activity (%±SD)			Thermostability	
			toward barley β-glucan	toward lichenan	toward barley β-glucan with 5mM $CaCl_2$	half-life time at 90°C (h)	half-life time at 100°C (h)
wild-type TMDG_GH12-2			237.38±6.1	161.87±5.2	130±2.9	19±0.5	0.5±0.03
Glu147	E147A	catalytic (nucleophile)	0	0	4.84±0.82	0	0
Glu240	E240A	catalytic (acid/base)	0.59±0.34	0	1.51±0.38	0.99±0.15	0
Glu233	E233A	influenced the secondary structure	81.4±2.63	98.47±3.4	89.69±2.5	9.16±0.46	0.48±0.04
Glu30	E30A		90.53±1.05	90.08±1.24	107.5±0.0001	14.58±0.73	0.4±0.02
Glu236	E236A		0	10.46±1.46	0	0.47±0.05	0
Glu77	E77A		5.42±2	1.91±1.84	3.41±1.22	1.24±0.08	0
Tyr28	Y28A	participating in substrate binding	85.05±7.07	84.35±9.17	67.5±4.75	0.85±0.043	0.34±0.017
Ser70	S70A		52.3±12.35	91.68±4.05	94.62±10.55	9.34±0.47	0.31±0.016
Trp12	W12A		123.77±4	99.92±3.4	144.2±5.75	8.56±0.43	0.32±0.01
Trp34	W34A		21.58±4.48	28.93±5.62	18±5.54	0.53±0.026	0
Trp130	W130A		37.75±2.79	51.22±2.19	29.67±3.38	9.23±0.46	0.77±0.038
Met149	M149A		0	0	5.02±1.58	0	0
Trp151	W151A		1.45±1.25	4.8±3.45	0	0.63±0.03	0
Asp18	D18A	participating in binding of Ca^{2+} ions	95.47±2.37	83.44±2.81	48.54±3.46	0.88±0.044	0
Asn86	N86A		57.35±2.67	65.73±2.48	39.53±6.08	11.69±0.58	0.68±0.03
Asp92	D92A		78.29±2.39	98.7±2.31	73.85±0.88	7.4±0.37	0.6±0.03
Trp85	W85A	unknown influence	69.16±3.6	65.73±2.48	57.55±7.58	0.88±0.044	0
Lys83	K83A		114.5±4.8	90.61±3.13	62.95±3.29	1.97±0.1	0
Asn32	N32A		96.06±7.04	82.52±11	39.82±10.24	4.23±0.21	0
Asn82	N82A		89.14±4.53	76.11±3.6	54.89±6.75	9.75±0.49	0.47±0.02
Asn88	N88A		144.82±2.27	114.27±6.55	148.18±2.95	9.2±0.46	0.4±0.02
Asn87	N87A		46.98±3.97	51.22±4.14	20.29±6.98	7.62±0.39	0.46±0.02

Table S7. Characteristics of representatives of GH12 family with optimal activity higher than 45 °C.

Protein id	Sequence id	Uniprot id	Domain*	Organism	Type of enzyme	3D structure	T opt	pH opt	Thermostability, half-life	Substrate specificity	Reference
**TMDG_GH12-1**			**A**	***Thermocccoccus* sp. 2319x1**	**recombinant**		**100**	**5**	**45 h at 100 °C, 288 h at 90 °C**	CMC, beta-glucan, lichenan, xylan, arabinoxylan	this work
**TMDG_GH12-2**			**A**	***Thermocccoccus* sp. 2319x1**	**recombinant**		**90**	**9**	**0.6 h at 100 °C, 19 h at 90 °C**	CMC, beta-glucan, lichenan	this work
EglA	NP_578583.1	Q9V2T1	A	*Pyrococcus furiosus*	recombinant	3VGI[A]	100	6	40 h, 95 °C	CMC, beta-glucan, lichenan, xylan	[1]
cel12E	A0A0F7YYA5		A	*Thermococcus* enrich	recombinant		92	5.5	2 h at 92 °C	CMC, beta-glucan, lichenan, PASC, HEC, xyloglucan, arabinoxylan, glucomannan	[2]
CelA2	SSO1949	Q97X08	A	*Sulfolobus solfataricus* P2	recombinant		80	1.8	8 h	CMC, cellooligosaccharides	[3]
CelA1 (CelB)	SSO1354	Q97YG7	A	*Sulfolobus solfataricus* P3	native		95	3.5	53 min	CMC, Beechwood xylan, Oat spelt xylan, Birchwood xylan, Arabinan, Debranched arabinan	[4]
SsGluC	LT221867	A0A1B5G0A2	A	*Saccharolobus shibatae*	recombinant		95-100	3.0-5.0	30 min - 30% at 90 °C, 2h - 80% at 85 °C	CMC, beta-glucan, lichenan, xylan	[5]
TCel6	ABW02444.1	A8M9X3	A	*Caldivirga maquilingensis* IC-167	recombinant		85	5.5	2 h retained 26% at 60 °C	CMC, AMC, filter paper, MCC (cotton linters)	[6]
Cel12A; CelA	AGL50456.1	Q60032	B	*Thermotoga maritima* DSM 3109 (MSB8)	recombinant	3AMH	85	6	30 min, 95 °C	CMC, beta-glucan, AMC, pNPGlu, oat spelt xylan	[13]
CelB	AAD36592.1	G4FFR3	B	*Thermotoga maritima* DSM 3109	recombinant		85	6	30 min, 95 °C	CMC, beta-glucan, AMC, pNPGlu, oat spelt xylan	[13]
Cel12A	AAB65594.1	O33897	B	*Rhodothermus marinus*	recombinant	1H0B[A,B]	100	6.0-7.0	retained 45% at 90 °C after 8 h	CMC, lichenan, glucomannan	[14]
CelS	AFI91032.1	P16630	B	*Pectobacterium carotovorum*	recombinant		45-55	6.8	nd	CMC, HEC, beta-glucan, xylan	[15]
Cel12A; BlXG12	AAU42138.1	Q65FM6	B	*Bacillus licheniformis*	recombinant	2JEM[A,B]	55	5.5	nd	CMC, xyloglucan, beta-glucan, glucomannan	[16]
cel12A	AAF91283.1	Q9KIH1	B	*Streptomyces* sp. 11AG8	recombinant	1OA4[A]	50	8	after 30 min at 70 °C fully inactivated	CMC	[17]
STHERM_c20650	ADN02999.1	E0RQU3	B	*Spirochaeta thermophila* DSM 6578	recombinant		nd	nd	nd	CMC, beta-glucan,	[18]
AcCel12B	ABK52392.1	A0LSI2	B	*Acidothermus cellulolyticus* 11B	recombinant		75	4.5	2 h at 70 °C, 12 min at 75 °C	CMC, AMC, RAC, MCC	[19]
**Protein id**	**Sequence id**	**Uniprot id**	**Domain**	**Organism**	**Type of enzyme**	**3D structure**	**T opt**	**pH opt**	**Thermostability, half-life**	**Substrate specificity**	**Reference**

GuxA	ABK52388.1	A0LSH8	B	*Acidothermus cellulolyticus* 11B	recombinant	7MKR[A]	75	5.5	nd	CMC, AMC, xylan	[20]
CelB	AAC02965.2	O31030	B	*Pectobacterium carotovorum* subsp. *carotovorum* LY34	recombinant		50	6.8	nd	CMC	[21]
CelB	AAB71950.1	Q54331	B	*Streptomyces lividans* 1326	native	1NLR[A]	50	6.5	nd	CMC	[22]
CelStrep	CCI74016.1	I7L8N7	B	*Streptomyces* sp. G12	recombinant		50	6.2	96 h at 50 °C, 24 h at 60 °C	CMC	[23]
CelA	AAC95059.1	O08428	B	*Thermotoga neapolitana*	recombinant		95	6	nd	CMC, AMC, MCC	[24]
CelB	AAC95060.1	P96492	B	*Thermotoga neapolitana*	recombinant		106	6-6.6	130 min at 106 °C, 26 min 110 °C	CMC, AMC, MCC	[24]
LC-CelA	AHL27894.1	W8PWF3	B	uncultured bacterium	recombinant	3WX5	90	5.0-8.0	fully retained at 90 °C after 30 min incubation	CMC	[25]
RSC-EG1	AFD36891.1	H9BVK4	B	uncultured bacterium	recombinant		65	6	40 min at 70 °C	CMC, beta-glucan, lichenan, laminarin	[26]
EglD;AtEglD;AtGH12;ATEG_09894	EAU30085.1	Q0C8U0	E	*Aspergillus terreus* NIH2624	recombinant	+	54	6.5	nd	β-glucan, konjac mannan and xyloglucan	[27]
EglA	CAK46524.1	A2R322	E	*Aspergillus niger* ATCC 10574	recombinant		nd	nd	nd		[28]
EG	AIX97359.1	O74705	E	*Aspergillus niger* HO	recombinant		70	3.5	3 h at 70 °C	CMC, AMC, xylan, xyloglucan	[29]
Egh12	THITE_2117762		E	Thielavia terrestris	recombinant	+	70	4.6	30 min 70 °C	CMC, beta-glucan, xylan, xyloglucan	[30]

Domain*: A – Archaea, B – Bacteria, E – Eukaryota

Table S8. Primers designed for *tmdg_gh12-1* and *tmdg_gh12-2* gene fragments.

**Gene *tmdg_gh12-1*(coordinates: 1357-2361)**	**Gene *tmdg_gh12-2* (coordinates: 2428-3267)**
Forward: 5`- GGTGATGATGATGAC**AAG** ACAACGACTACTACTCCTCCAGGGA-3`	Forward: 5`- GGTGATGATGATGAC**AAG** GTCATTAAGATAAGGTACCCGGACG-3`
Reverse: 5`- GGAGATGGGAAGTCA**TTA** TGTCGTCTGCGATGTTGTGGT-3`	Reverse: 5`- GGAGATGGGAAGTCA**TTA** TGATGGAGCAGACCCTTCC-3`

Specific sequences are underlined; start- and stop codons are bolded.

Table S9. Primer sequences (5' to 3') for alanine scanning and cross mutagenesis.

	**Alanine scanning mutagenesis**
W12A_For	ggggcctccggcgcctgcccatcgtc
W12A_Rev	gacgatgggcaggcgccggaggcccc
D18A_For	ccccgtccccggcgattggggcc
D18A_Rev	ggccccaatcgccggggacgggg
Y28A_For	cacgggtttatttcgatggcgaactctgggttcccgtc
Y28A_Rev	gacgggaacccagagttcgccatcgaaataaacccgtg
E30A_For	gtatattccacgggtttattgcgatgtagaactctgggttc
E30A_Rev	gaacccagagttctacatcgcaataaacccgtggaatatac
N32A_For	gtatattccacggggctatttcgatgtagaactctgggttccc
N32A_Rev	gggaacccagagttctacatcgaaatagccccgtggaatatac
W34A_For	cttcagcgctctgtatattcgccgggtttatttcgatgtaga
W34A_Rev	tctacatcgaaataaacccggcgaatatacagagcgctgaag
S70A_For	ccgtgcacccacgcgccgccgttttta
S70A_Rev	taaaaacggcggcgcgtgggtgcacgg
E77A_For	gttgccgtagaatatcgcgggatatccgtgcac
E77A_Rev	gtgcacggatatcccgcgatattctacggcaac
N82A_For	ttgttgttccagggcttggcgccgtagaatatctcggg
N82A_Rev	cccgagatattctacggcgccaagccctggaacaacaa
K83A_For	gtagttgttgttccagggcgcgttgccgtagaatatctcg
K83A_Rev	cgagatattctacggcaacgcgccctggaacaacaactac
N86A_For	ggtagcgtagttgttggcccagggcttgttgccg
N86A_Rev	cggcaacaagccctgggccaacaactacgctacc
N87A_For	gcaacaagccctggaacgccaactacgctaccgatg
N87A_Rev	catcggtagcgtagttggcgttccagggcttgttgc
N88A_For	cccatcggtagcgtaggcgttgttccagggcttg
N88A_Rev	caagccctggaacaacgcctacgctaccgatggg
D92A_For	gtggaacctccccagcggtagcgtagttg
D92A_Rev	caactacgctaccgctggggaggttccac
W130A_For	ggctccctcgtgagcgccgactcgattgcaaa
W130A_Rev	tttgcaatcgagtcggcgctcacgagggagcc
N138A_For	gctgtttattccgctggccctccagggctccctc
N138A_Rev	gagggagccctggagggccagcggaataaacagc
E147A_For	cagcgacgagcaggcgctcatgatatggc
E147A_Rev	gccatatcatgagcgcctgctcgtcgctg
M149A_For	cgtaatacagccatatcgcgagctcctgctcgtcgc
M149A_Rev	gcgacgagcaggagctcgcgatatggctgtattacg
W151A_For	cgtcgtaatacagcgctatcatgagctcctgctcgtcg
W151A_Rev	cgacgagcaggagctcatgatagcgctgtattacgacg
E233A_For	ccaacctcaacgtctgccaggtacagctcgg
E233A_Rev	ccgagctgtacctggcagacgttgaggttgg
E236A_For	cgtattcggttccaaccgcaacgtcttccaggtac
E236A_Rev	gtacctggaagacgttgcggttggaaccgaatacg
E240A_For	gagggcgttccgtatgcggttccaacctcaa
E240A_Rev	ttgaggttggaaccgcatacggaacgccctc
	**Cross mutagenesis**
TMDG_GH12-1 Q185W_For	cgccttcgatgtagagccataccattatctcgtagtccc
TMDG_GH12-1 Q185W_Rev	gggactacgagataatggtatggctctacatcgaaggcg
TMDG_GH12-2 W151Q_For	cgtcgtaatacagctgtatcatgagctcctgctcgtcg
TMDG_GH12-2 W151Q_Rev	cgacgagcaggagctcatgatacagctgtattacgacg

Table S10. TMDG_GH12 sequences.

ID	Description	Nucleotide sequence	Amino acid sequence
TMDG_GH12-1	A fragment of MDG ALV63957.1 from *Thermococcus* sp. 2319x1, containing GH12-1 domain and elements of expression vector pPLATE51 (in bold and underlined)	**ATGGCGGGTTCTCATCATCATCATCATCAT<br>GGTATGGCTAGCATGACTGGTGGACAGCA<br>AATGGGTCGCTCCGGTGATGATGATGACA<br>AG**ACAACGACTACTACTCCTCCAGGGAGCAT<br>TCCCTTTGAAACCGTGAACATTCTCCCCACG<br>AGCTCCCAGTACGAGGGAACCAGCGTGGAG<br>GTCGTATGTGACGGAACCCAGTGTGCCTCCA<br>GCGTTTGGGGAGCACCGAACCTCTGGGGAGT<br>CGTTAAAATCGGAAACGCCACAATGGATCCC<br>AACGTTTGGGGCTGGGAGGATGTTTACAGGA<br>CTGCACCTCAGGACATTGGAACCGGCAGCAC<br>AAAGATGGAGATAGGGAACGGGGTGCTCAA<br>AGTCACGAGCCTTTGGAACATTAACATGCAT<br>CCCAAATACAACACAATGGCATACCATGAGG<br>TCATATACGGTGCCAAGCCGTGGGGCAACCA<br>GCCAATAAATGCCCAGAACTTCGTGCTCCCG<br>ATACAGGTCTCCCAGCTTCCAAGGATACTCG<br>TTGACACAAAGTACACACTCGAAAAGAGCTT<br>CCCAGGAAACAACTTCGCCTTTGAAGCGTGG<br>CTCTTCAAAGACACCGACAACATGAGGGCCC<br>CCGGCCAGGGGGACTACGAGATAATGGTAC<br>AGCTCTACATCGAAGGCGGCTATCCAGCAGG<br>CTACGACAAGGGACCCGTCCTCACCGTTGAT<br>GTTCCAATAATCGTCGATGGAAAGCTTGTAA<br>ACCAGACTTTTGAGCTATACGACGTCGTAGC<br>GGATGCTGGGTGGAGGTTCTTAACCTTCAAG<br>TCAACCAAGAACTATAACGGTTCAGAGGTCG<br>TATTCGACTACACCAAGTTCATAGAGATAGT<br>TGACGGCTACCTCAGCGGTGGCAACCTCACG<br>AACCACTACCTAATGTCCCTGGAATTCGGTA<br>CCGAGGTATACACCAACGGGTGCAGCTCGTT<br>CCCCTGCACCGTGGACGTAAGGTGGACCCTT<br>GACAAGTACAGGTTCATTCTGGCCCCCAATA<br>CAATGACCACTGAGGAGGCCATGAGCGTTCT<br>CATCGGAGAGGTCCAGCCTCCCGCTTCCACC<br>ACAACATCGCAGACGACA**TAA**	**MAGSHHHHHHGMASMT<br>GGQQMGRSGDDDDK**TTT<br>TTPPGSIPFETVNILPTSSQY<br>EGTSVEVVCDGTQCASSV<br>WGAPNLWGVVKIGNATM<br>DPNVWGWEDVYRTAPQDI<br>GTGSTKMEIGNGVLKVTSL<br>WNINMHPKYNTMAYHEVI<br>YGAKPWGNQPINAQNFVL<br>PIQVSQLPRILVDTKYTLEK<br>SFPGNNFAFEAWLFKDTD<br>NMRAPGQGDYEIMVQLYI<br>EGGYPAGYDKGPVLTVDV<br>PIIVDGKLVNQTFELYDVV<br>ADAGWRFLTFKSTKNYNG<br>SEVVFDYTKFIEIVDGYLSG<br>GNLTNHYLMSLEFGTEVY<br>TNGCSSFPCTVDVRWTLD<br>KYRFILAPNTMTTEEAMSV<br>LIGEVQPPASTTTSQTT
TMDG_GH12-2	A fragment of MDG ALV63957.1 from *Thermococcus* sp. 2319x1, containing GH12-2 domain and elements of expression vector pPLATE51 (in bold and underlined)	**ATGGCGGGTTCTCATCATCATCATCATCAT<br>GGTATGGCTAGCATGACTGGTGGACAGCA<br>AATGGGTCGCTCCGGTGATGATGATGACA<br>AG**GTCATTAAGATAAGGTACCCGGACGATG<br>GGCAGTGGCCGGAGGCCCCAATCGACGGGG<br>ACGGGGACGGGAACCCAGAGTTCTACATCG<br>AAATAAACCCGTGGAATATACAGAGCGCTG<br>AAGGCTACGCCGAGATGACCTACAACCTTAG<br>CACAGGCGTCCTCCACTACGTCCAAGCCTTA<br>GACGATATAACCCTTAAAAACGGCGGCTCGT<br>GGGTGCACGGATATCCCGAGATATTCTACGG<br>CAACAAGCCCTGGAACAACAACTACGCTACC<br>GATGGGGAGGTTCCACTTCCAGGAAAAGTCT<br>CGAACCTGAGCAACTTCTACCTGAGCGTAAG<br>CTACAAGCTGCTGCCAAAGAACGGCCTTCCT<br>ATCAACTTTGCAATCGAGTCGTGGCTCACGA<br>GGGAGCCCTGGAGGAACAGCGGAATAAACA<br>GCGACGAGCAGGAGCTCATGATATGGCTGTA<br>TTACGACGGACTCCAGCCGGCTGGCTCAAAG<br>GTCAAGGAAATCATTGTCCCGATAGTGGTGA<br>ACGGCACCCCAGTGAACGCTACCTTCGAAGT<br>CTGGAAGGCGAACATCGGCTGGGAGTACAT<br>AGCCTTCAGGATAAAGACCCCAATAAAGGA<br>GGGAACCGTCACTATACCGTACGGAGCCTTC	**MAGSHHHHHHGMASMT<br>GGQQMGRSGDDDDK**VIK<br>IRYPDDGQWPEAPIDGDGD<br>GNPEFYIEINPWNIQSAEGY<br>AEMTYNLSTGVLHYVQAL<br>DDITLKNGGSWVHGYPEIF<br>YGNKPWNNNYATDGEVPL<br>PGKVSNLSNFYLSVSYKLL<br>PKNGLPINFAIESWLTREP<br>WRNSGINSDEQELMIWLY<br>YDGLQPAGSKVKEIIVPIVV<br>NGTPVNATFEVWKANIGW<br>EYIAFRIKTPIKEGTVTIPYG<br>AFISAAANVTSLANYTELY<br>LEDVEVGTEYGTPSTTSAH<br>LEWWFYNVSLEYRPGEPL<br>LSQPPAEGSAPS

		ATCAGCGCCGCCGCAAACGTAACGAGCCTAG<br>CTAACTACACCGAGCTGTACCTGGAAGACGT<br>TGAGGTTGGAACCGAATACGGAACGCCCTCA<br>ACCACTAGCGCACACCTCGAGTGGTGGTTCT<br>ACAACGTCTCGCTCGAGTACAGGCCTGGAGA<br>GCCACTGCTCTCACAGCCACCTGCGGAAGGG<br>TCTGCTCCATCA**TAA**	
TMDG_GH12-1_Q185W	A fragment of MDG ALV63957.1 from *Thermococcus* sp. 2319x1, containing GH12-1 domain with substitution Q->W in 185 position and elements of expression vector pPLATE51 (in bold and underlined)	**ATGGCGGGTTCTCATCATCATCATCATCAT**<br>**GGTATGGCTAGCATGACTGGTGGACAGCA**<br>**AATGGGTCGCTCCGGTGATGATGATGACA**<br>**AG**ACAACGACTACTACTCCTCCAGGGAGCAT<br>TCCCTTTGAAACCGTGAACATTCTCCCCACG<br>AGCTCCCAGTACGAGGGAACCAGCGTGGAG<br>GTCGTATGTGACGGAACCCAGTGTGCCTCCA<br>GCGTTTGGGGAGCACCGAACCTCTGGGGAGT<br>CGTTAAAATCGGAAACGCCACAATGGATCCC<br>AACGTTTGGGGCTGGGAGGATGTTTACAGGA<br>CTGCACCTCAGGACATTGGAACCGGCAGCAC<br>AAAGATGGAGATAGGGAACGGGGTGCTCAA<br>AGTCACGAGCCTTTGGAACATTAACATGCAT<br>CCCAAATACAACACAATGGCATACCATGAGG<br>TCATATACGGTGCCAAGCCGTGGGGCAACCA<br>GCCAATAAATGCCCAGAACTTCGTGCTCCCG<br>ATACAGGTCTCCCAGCTTCCAAGGATACTCG<br>TTGACACAAAGTACACACTCGAAAAGAGCTT<br>CCCAGGAAACAACTTCGCCTTTGAAGCGTGG<br>CTCTTCAAAGACACCGACAACATGAGGGCCC<br>CCGGCCAGGGGGACTACGAGATAATGGTAT<br>GGCTCTACATCGAAGGCGGCTATCCAGCAGG<br>CTACGACAAGGGACCCGTCCTCACCGTTGAT<br>GTTCCAATAATCGTCGATGGAAAGCTTGTAA<br>ACCAGACTTTTGAGCTATACGACGTCGTAGC<br>GGATGCTGGGTGGAGGTTCTTAACCTTCAAG<br>TCAACCAAGAACTATAACGGTTCAGAGGTCG<br>TATTCGACTACACCAAGTTCATAGAGATAGT<br>TGACGGCTACCTCAGCGGTGGCAACCTCACG<br>AACCACTACCTAATGTCCCTGGAATTCGGTA<br>CCGAGGTATACACCAACGGGTGCAGCTCGTT<br>CCCCTGCACCGTGGACGTAAGGTGGACCCTT<br>GACAAGTACAGGTTCATTCTGGCCCCCAATA<br>CAATGACCACTGAGGAGGCCATGAGCGTTCT<br>CATCGGAGAGGTCCAGCCTCCCGCTTCCACC<br>ACAACATCGCAGACGACA**TAA**	**MAGSHHHHHHGMASMT**<br>**GGQQMGRSGDDDDK**TTT<br>TTPPGSIPFETVNILPTSSQY<br>EGTSVEVVCDGTQCASSV<br>WGAPNLWGVVKIGNATM<br>DPNVWGWEDVYRTAPQDI<br>GTGSTKMEIGNGVLKVTSL<br>WNINMHPKYNTMAYHEVI<br>YGAKPWGNQPINAQNFVL<br>PIQVSQLPRILVDTKYTLEK<br>SFPGNNFAFEAWLFKDTD<br>NMRAPGQGDYEIMVWLYI<br>EGGYPAGYDKGPVLTVDV<br>PIIVDGKLVNQTFELYDVV<br>ADAGWRFLTFKSTKNYNG<br>SEVVFDYTKFIEIVDGYLSG<br>GNLTNHYLMSLEFGTEVY<br>TNGCSSFPCTVDVRWTLD<br>KYRFILAPNTMTTEEAMSV<br>LIGEVQPPASTTTSQTT
TMDG_GH12-2_W151Q	A fragment of MDG ALV63957.1 from *Thermococcus* sp. 2319x1, containing GH12-2 domain with substitution W->Q in 151 position and elements of expression vector pPLATE51 (in bold and underlined)	**ATGGCGGGTTCTCATCATCATCATCATCAT**<br>**GGTATGGCTAGCATGACTGGTGGACAGCA**<br>**AATGGGTCGCTCCGGTGATGATGATGACA**<br>**AG**GTCATTAAGATAAGGTACCCGGACGATG<br>GGCAGTGGCCGGAGGCCCCAATCGACGGGG<br>ACGGGGACGGGAACCCAGAGTTCTACATCG<br>AAATAAACCCGTGGAATATACAGAGCGCTG<br>AAGGCTACGCCGAGATGACCTACAACCTTAG<br>CACAGGCGTCCTCCACTACGTCCAAGCCTTA<br>GACGATATAACCCTTAAAAACGGCGGCTCGT<br>GGGTGCACGGATATCCCGAGATATTCTACGG<br>CAACAAGCCCTGGAACAACAACTACGCTACC<br>GATGGGGAGGTTCCACTTCCAGGAAAAGTCT<br>CGAACCTGAGCAACTTCTACCTGAGCGTAAG<br>CTACAAGCTGCTGCCAAAGAACGGCCTTCCT<br>ATCAACTTTGCAATCGAGTCGTGGCTCACGA<br>GGGAGCCCTGGAGGAACAGCGGAATAAACA<br>GCGACGAGCAGGAGCTCATGATAcaGCTGTA<br>TTACGACGGACTCCAGCCGGCTGGCTCAAAG	**MAGSHHHHHHGMASMT**<br>**GGQQMGRSGDDDDK**VIK<br>IRYPDDGQWPEAPIDGDGD<br>GNPEFYIEINPWNIQSAEGY<br>AEMTYNLSTGVLHYVQAL<br>DDITLKNGGSWVHGYPEIF<br>YGNKPWNNNYATDGEVPL<br>PGKVSNLSNFYLSVSYKLL<br>PKNGLPINFAIESWLTREP<br>WRNSGINSDEQELMIQLYY<br>DGLQPAGSKVKEIIVPIVVN<br>GTPVNATFEVWKANIGWE<br>YIAFRIKTPIKEGTVTIPYG<br>AFISAAANVTSLANYTELY<br>LEDVEVGTEYGTPSTTSAH<br>LEWWFYNVSLEYRPGEPL<br>LSQPPAEGSAPS

	GTCAAGGAAATCATTGTCCCGATAGTGGTGA<br>ACGGCACCCCAGTGAACGCTACCTTCGAAGT<br>CTGGAAGGCGAACATCGGCTGGGAGTACAT<br>AGCCTTCAGGATAAAGACCCCAATAAAGGA<br>GGGAACCGTCACTATACCGTACGGAGCCTTC<br>ATCAGCGCCGCCGCAAACGTAACGAGCCTAG<br>CTAACTACACCGAGCTGTACCTGGAAGACGT<br>TGAGGTTGGAACCGAATACGGAACGCCCTCA<br>ACCACTAGCGCACACCTCGAGTGGTGGTTCT<br>ACAACGTCTCGCTCGAGTACAGGCCTGGAGA<br>GCCACTGCTCTCACAGCCACCTGCGGAAGGG<br>TCTGCTCCATCA**TAA**	
	**Fragments of MDG ALV63957.1 from *Thermococcus* sp. 2319x1, containing GH12-2 domain with single aminoacid substitutions to alanine, and elements of expression vector pPLATE51 (in bold and underlined)**	
TMDG_GH12-2_W12A	**ATGGCGGGTTCTCATCATCATCATCATCA**<br>**TGGTATGGCTAGCATGACTGGTGGACAG**<br>**CAAATGGGTCGCTCCGGTGATGATGATG**<br>**ACAAG**GTCATTAAGATAAGGTACCCGGACG<br>ATGGGCAGgcGCCGGAGGCCCCAATCGACG<br>GGGACGGGGACGGGAACCCAGAGTTCTACA<br>TCGAAATAAACCCGTGGAATATACAGAGCG<br>CTGAAGGCTACGCCGAGATGACCTACAACC<br>TTAGCACAGGCGTCCTCCACTACGTCCAAG<br>CCTTAGACGATATAACCCTTAAAAACGGCG<br>GCTCGTGGGTGCACGGATATCCCGAGATAT<br>TCTACGGCAACAAGCCCTGGAACAACAACT<br>ACGCTACCGATGGGGAGGTTCCACTTCCAG<br>GAAAAGTCTCGAACCTGAGCAACTTCTACC<br>TGAGCGTAAGCTACAAGCTGCTGCCAAAGA<br>ACGGCCTTCCTATCAACTTTGCAATCGAGTC<br>GTGGCTCACGAGGGAGCCCTGGAGGAACAG<br>CGGAATAAACAGCGACGAGCAGGAGCTCAT<br>GATATGGCTGTATTACGACGGACTCCAGCC<br>GGCTGGCTCAAAGGTCAAGGAAATCATTGT<br>CCCGATAGTGGTGAACGGCACCCCAGTGAA<br>CGCTACCTTCGAAGTCTGGAAGGCGAACAT<br>CGGCTGGGAGTACATAGCCTTCAGGATAAA<br>GACCCCAATAAAGGAGGGAACCGTCACTAT<br>ACCGTACGGAGCCTTCATCAGCGCCGCCGC<br>AAACGTAACGAGCCTAGCTAACTACACCGA<br>GCTGTACCTGGAAGACGTTGAGGTTGGAAC<br>CGAATACGGAACGCCCTCAACCACTAGCGC<br>ACACCTCGAGTGGTGGTTCTACAACGTCTCG<br>CTCGAGTACAGGCCTGGAGAGCCACTGCTC<br>TCACAGCCACCTGCGGAAGGGTCTGCTCCA<br>TCA**TAA**	**MAGSHHHHHHGMASMT**<br>**GGQQMGRSGDDDDK**VIK<br>IRYPDDGQAPEAPIDGDGD<br>GNPEFYIEINPWNIQSAEGY<br>AEMTYNLSTGVLHYVQAL<br>DDITLKNGGSWVHGYPEIF<br>YGNKPWNNNYATDGEVPL<br>PGKVSNLSNFYLSVSYKLL<br>PKNGLPINFAIESWLTREP<br>WRNSGINSDEQELMIWLY<br>YDGLQPAGSKVKEIIVPIVV<br>NGTPVNATFEVWKANIGW<br>EYIAFRIKTPIKEGTVTIPYG<br>AFISAAANVTSLANYTELY<br>LEDVEVGTEYGTPSTTSAH<br>LEWWFYNVSLEYRPGEPL<br>LSQPPAEGSAPs
TMDG_GH12-2_D18A	**ATGGCGGGTTCTCATCATCATCATCATCA**<br>**TGGTATGGCTAGCATGACTGGTGGACAG**<br>**CAAATGGGTCGCTCCGGTGATGATGATG**<br>**ACAAG**GTCATTAAGATAAGGTACCCGGACG<br>ATGGGCAGTGGCCGGAGGCCCCAATCGcCG<br>GGGACGGGGACGGGAACCCAGAGTTCTACA<br>TCGAAATAAACCCGTGGAATATACAGAGCG<br>CTGAAGGCTACGCCGAGATGACCTACAACC<br>TTAGCACAGGCGTCCTCCACTACGTCCAAG<br>CCTTAGACGATATAACCCTTAAAAACGGCG<br>GCTCGTGGGTGCACGGATATCCCGAGATAT<br>TCTACGGCAACAAGCCCTGGAACAACAACT<br>ACGCTACCGATGGGGAGGTTCCACTTCCAG<br>GAAAAGTCTCGAACCTGAGCAACTTCTACC<br>TGAGCGTAAGCTACAAGCTGCTGCCAAAGA	**MAGSHHHHHHGMASMT**<br>**GGQQMGRSGDDDDK**VIK<br>IRYPDDGQWPEAPIAGDGD<br>GNPEFYIEINPWNIQSAEGY<br>AEMTYNLSTGVLHYVQAL<br>DDITLKNGGSWVHGYPEIF<br>YGNKPWNNNYATDGEVPL<br>PGKVSNLSNFYLSVSYKLL<br>PKNGLPINFAIESWLTREP<br>WRNSGINSDEQELMIWLY<br>YDGLQPAGSKVKEIIVPIVV<br>NGTPVNATFEVWKANIGW<br>EYIAFRIKTPIKEGTVTIPYG<br>AFISAAANVTSLANYTELY<br>LEDVEVGTEYGTPSTTSAH

		ACGGCCTTCCTATCAACTTTGCAATCGAGTC<br>GTGGCTCACGAGGGAGCCCTGGAGGAACAG<br>CGGAATAAACAGCGACGAGCAGGAGCTCAT<br>GATATGGCTGTATTACGACGGACTCCAGCC<br>GGCTGGCTCAAAGGTCAAGGAAATCATTGT<br>CCCGATAGTGGTGAACGGCACCCCAGTGAA<br>CGCTACCTTCGAAGTCTGGAAGGCGAACAT<br>CGGCTGGGAGTACATAGCCTTCAGGATAAA<br>GACCCCAATAAAGGAGGGAACCGTCACTAT<br>ACCGTACGGAGCCTTCATCAGCGCCGCCGC<br>AAACGTAACGAGCCTAGCTAACTACACCGA<br>GCTGTACCTGGAAGACGTTGAGGTTGGAAC<br>CGAATACGGAACGCCCTCAACCACTAGCGC<br>ACACCTCGAGTGGTGGTTCTACAACGTCTCG<br>CTCGAGTACAGGCCTGGAGAGCCACTGCTC<br>TCACAGCCACCTGCGGAAGGGTCTGCTCCA<br>TCA**TAA**	LEWWFYNVSLEYRPGEPL<br>LSQPPAEGSAPs
TMDG_GH12-2_Y28A		**ATGGCGGGTTCTCATCATCATCATCATCA**<br>**TGGTATGGCTAGCATGACTGGTGGACAG**<br>**CAAATGGGTCGCTCCGGTGATGATGATG**<br>**ACAAG**GTCATTAAGATAAGGTACCCGGACG<br>ATGGGCAGTGGCCGGAGGCCCCAATCGACG<br>GGGACGGGGACGGGAACCCAGAGTTCgcCA<br>TCGAAATAAACCCGTGGAATATACAGAGCG<br>CTGAAGGCTACGCCGAGATGACCTACAACC<br>TTAGCACAGGCGTCCTCCACTACGTCCAAG<br>CCTTAGACGATATAACCCTTAAAAACGGCG<br>GCTCGTGGGTGCACGGATATCCCGAGATAT<br>TCTACGGCAACAAGCCCTGGAACAACAACT<br>ACGCTACCGATGGGGAGGTTCCACTTCCAG<br>GAAAAGTCTCGAACCTGAGCAACTTCTACC<br>TGAGCGTAAGCTACAAGCTGCTGCCAAAGA<br>ACGGCCTTCCTATCAACTTTGCAATCGAGTC<br>GTGGCTCACGAGGGAGCCCTGGAGGAACAG<br>CGGAATAAACAGCGACGAGCAGGAGCTCAT<br>GATATGGCTGTATTACGACGGACTCCAGCC<br>GGCTGGCTCAAAGGTCAAGGAAATCATTGT<br>CCCGATAGTGGTGAACGGCACCCCAGTGAA<br>CGCTACCTTCGAAGTCTGGAAGGCGAACAT<br>CGGCTGGGAGTACATAGCCTTCAGGATAAA<br>GACCCCAATAAAGGAGGGAACCGTCACTAT<br>ACCGTACGGAGCCTTCATCAGCGCCGCCGC<br>AAACGTAACGAGCCTAGCTAACTACACCGA<br>GCTGTACCTGGAAGACGTTGAGGTTGGAAC<br>CGAATACGGAACGCCCTCAACCACTAGCGC<br>ACACCTCGAGTGGTGGTTCTACAACGTCTCG<br>CTCGAGTACAGGCCTGGAGAGCCACTGCTC<br>TCACAGCCACCTGCGGAAGGGTCTGCTCCA<br>TCA**TAA**	**MAGSHHHHHHGMASMT**<br>**GGQQMGRSGDDDDK**VIK<br>IRYPDDGQWPEAPIDGDGD<br>GNPEFAIEINPWNIQSAEGY<br>AEMTYNLSTGVLHYVQAL<br>DDITLKNGGSWVHGYPEIF<br>YGNKPWNNNYATDGEVPL<br>PGKVSNLSNFYLSVSYKLL<br>PKNGLPINFAIESWLTREP<br>WRNSGINSDEQELMIWLY<br>YDGLQPAGSKVKEIIVPIVV<br>NGTPVNATFEVWKANIGW<br>EYIAFRIKTPIKEGTVTIPYG<br>AFISAAANVTSLANYTELY<br>LEDVEVGTEYGTPSTTSAH<br>LEWWFYNVSLEYRPGEPL<br>LSQPPAEGSAPs
TMDG_GH12-2_E30A		**ATGGCGGGTTCTCATCATCATCATCATCA**<br>**TGGTATGGCTAGCATGACTGGTGGACAG**<br>**CAAATGGGTCGCTCCGGTGATGATGATG**<br>**ACAAG**GTCATTAAGATAAGGTACCCGGACG<br>ATGGGCAGTGGCCGGAGGCCCCAATCGACG<br>GGGACGGGGACGGGAACCCAGAGTTCTACA<br>TCGcAATAAACCCGTGGAATATACAGAGCG<br>CTGAAGGCTACGCCGAGATGACCTACAACC<br>TTAGCACAGGCGTCCTCCACTACGTCCAAG<br>CCTTAGACGATATAACCCTTAAAAACGGCG<br>GCTCGTGGGTGCACGGATATCCCGAGATAT<br>TCTACGGCAACAAGCCCTGGAACAACAACT<br>ACGCTACCGATGGGGAGGTTCCACTTCCAG<br>GAAAAGTCTCGAACCTGAGCAACTTCTACC	**MAGSHHHHHHGMASMT**<br>**GGQQMGRSGDDDDK**VIK<br>IRYPDDGQWPEAPIDGDGD<br>GNPEFYIAINPWNIQSAEG<br>YAEMTYNLSTGVLHYVQA<br>LDDITLKNGGSWVHGYPEI<br>FYGNKPWNNNYATDGEVP<br>LPGKVSNLSNFYLSVSYKL<br>LPKNGLPINFAIESWLTREP<br>WRNSGINSDEQELMIWLY<br>YDGLQPAGSKVKEIIVPIVV<br>NGTPVNATFEVWKANIGW<br>EYIAFRIKTPIKEGTVTIPYG<br>AFISAAANVTSLANYTELY

		TGAGCGTAAGCTACAAGCTGCTGCCAAAGA<br>ACGGCCTTCCTATCAACTTTGCAATCGAGTC<br>GTGGCTCACGAGGGAGCCCTGGAGGAACAG<br>CGGAATAAACAGCGACGAGCAGGAGCTCAT<br>GATATGGCTGTATTACGACGGACTCCAGCC<br>GGCTGGCTCAAAGGTCAAGGAAATCATTGT<br>CCCGATAGTGGTGAACGGCACCCCAGTGAA<br>CGCTACCTTCGAAGTCTGGAAGGCGAACAT<br>CGGCTGGGAGTACATAGCCTTCAGGATAAA<br>GACCCCAATAAAGGAGGGAACCGTCACTAT<br>ACCGTACGGAGCCTTCATCAGCGCCGCCGC<br>AAACGTAACGAGCCTAGCTAACTACACCGA<br>GCTGTACCTGGAAGACGTTGAGGTTGGAAC<br>CGAATACGGAACGCCCTCAACCACTAGCGC<br>ACACCTCGAGTGGTGGTTCTACAACGTCTCG<br>CTCGAGTACAGGCCTGGAGAGCCACTGCTC<br>TCACAGCCACCTGCGGAAGGGTCTGCTCCA<br>TCA**TAA**	LEDVEVGTEYGTPSTTSAH<br>LEWWFYNVSLEYRPGEPL<br>LSQPPAEGSAPs
TMDG_GH12-2_N32A		**ATGGCGGGTTCTCATCATCATCATCATCA**<br>**TGGTATGGCTAGCATGACTGGTGGACAG**<br>**CAAATGGGTCGCTCCGGTGATGATGATG**<br>**ACAAG**GTCATTAAGATAAGGTACCCGGACG<br>ATGGGCAGTGGCCGGAGGCCCCAATCGACG<br>GGGACGGGGACGGGAACCCAGAGTTCTACA<br>TCGAAATAgcCCCGTGGAATATACAGAGCGC<br>TGAAGGCTACGCCGAGATGACCTACAACCT<br>TAGCACAGGCGTCCTCCACTACGTCCAAGC<br>CTTAGACGATATAACCCTTAAAAACGGCGG<br>CTCGTGGGTGCACGGATATCCCGAGATATT<br>CTACGGCAACAAGCCCTGGAACAACAACTA<br>CGCTACCGATGGGGAGGTTCCACTTCCAGG<br>AAAAGTCTCGAACCTGAGCAACTTCTACCT<br>GAGCGTAAGCTACAAGCTGCTGCCAAAGAA<br>CGGCCTTCCTATCAACTTTGCAATCGAGTCG<br>TGGCTCACGAGGGAGCCCTGGAGGAACAGC<br>GGAATAAACAGCGACGAGCAGGAGCTCATG<br>ATATGGCTGTATTACGACGGACTCCAGCCG<br>GCTGGCTCAAAGGTCAAGGAAATCATTGTC<br>CCGATAGTGGTGAACGGCACCCCAGTGAAC<br>GCTACCTTCGAAGTCTGGAAGGCGAACATC<br>GGCTGGGAGTACATAGCCTTCAGGATAAAG<br>ACCCCAATAAAGGAGGGAACCGTCACTATA<br>CCGTACGGAGCCTTCATCAGCGCCGCCGCA<br>AACGTAACGAGCCTAGCTAACTACACCGAG<br>CTGTACCTGGAAGACGTTGAGGTTGGAACC<br>GAATACGGAACGCCCTCAACCACTAGCGCA<br>CACCTCGAGTGGTGGTTCTACAACGTCTCGC<br>TCGAGTACAGGCCTGGAGAGCCACTGCTCT<br>CACAGCCACCTGCGGAAGGGTCTGCTCCAT<br>CA**TAA**	**MAGSHHHHHHGMASMT**<br>**GGQQMGRSGDDDDK**VIK<br>IRYPDDGQWPEAPIDGDGD<br>GNPEFYIEIAPWNIQSAEGY<br>AEMTYNLSTGVLHYVQAL<br>DDITLKNGGSWVHGYPEIF<br>YGNKPWNNNYATDGEVPL<br>PGKVSNLSNFYLSVSYKLL<br>PKNGLPINFAIESWLTREP<br>WRNSGINSDEQELMIWLY<br>YDGLQPAGSKVKEIIVPIVV<br>NGTPVNATFEVWKANIGW<br>EYIAFRIKTPIKEGTVTIPYG<br>AFISAAANVTSLANYTELY<br>LEDVEVGTEYGTPSTTSAH<br>LEWWFYNVSLEYRPGEPL<br>LSQPPAEGSAPs
TMDG_GH12-2_W34A		**ATGGCGGGTTCTCATCATCATCATCATCA**<br>**TGGTATGGCTAGCATGACTGGTGGACAG**<br>**CAAATGGGTCGCTCCGGTGATGATGATG**<br>**ACAAG**GTCATTAAGATAAGGTACCCGGACG<br>ATGGGCAGTGGCCGGAGGCCCCAATCGACG<br>GGGACGGGGACGGGAACCCAGAGTTCTACA<br>TCGAAATAAACCCGgcGAATATACAGAGCG<br>CTGAAGGCTACGCCGAGATGACCTACAACC<br>TTAGCACAGGCGTCCTCCACTACGTCCAAG<br>CCTTAGACGATATAACCCTTAAAAACGGCG<br>GCTCGTGGGTGCACGGATATCCCGAGATAT<br>TCTACGGCAACAAGCCCTGGAACAACAACT<br>ACGCTACCGATGGGGAGGTTCCACTTCCAG	**MAGSHHHHHHGMASMT**<br>**GGQQMGRSGDDDDK**VIK<br>IRYPDDGQWPEAPIDGDGD<br>GNPEFYIEINPANIQSAEGY<br>AEMTYNLSTGVLHYVQAL<br>DDITLKNGGSWVHGYPEIF<br>YGNKPWNNNYATDGEVPL<br>PGKVSNLSNFYLSVSYKLL<br>PKNGLPINFAIESWLTREP<br>WRNSGINSDEQELMIWLY<br>YDGLQPAGSKVKEIIVPIVV<br>NGTPVNATFEVWKANIGW<br>EYIAFRIKTPIKEGTVTIPYG

		GAAAAGTCTCGAACCTGAGCAACTTCTACC<br>TGAGCGTAAGCTACAAGCTGCTGCCAAAGA<br>ACGGCCTTCCTATCAACTTTGCAATCGAGTC<br>GTGGCTCACGAGGGAGCCCTGGAGGAACAG<br>CGGAATAAACAGCGACGAGCAGGAGCTCAT<br>GATATGGCTGTATTACGACGGACTCCAGCC<br>GGCTGGCTCAAAGGTCAAGGAAATCATTGT<br>CCCGATAGTGGTGAACGGCACCCCAGTGAA<br>CGCTACCTTCGAAGTCTGGAAGGCGAACAT<br>CGGCTGGGAGTACATAGCCTTCAGGATAAA<br>GACCCCAATAAAGGAGGGAACCGTCACTAT<br>ACCGTACGGAGCCTTCATCAGCGCCGCCGC<br>AAACGTAACGAGCCTAGCTAACTACACCGA<br>GCTGTACCTGGAAGACGTTGAGGTTGGAAC<br>CGAATACGGAACGCCCTCAACCACTAGCGC<br>ACACCTCGAGTGGTGGTTCTACAACGTCTCG<br>CTCGAGTACAGGCCTGGAGAGCCACTGCTC<br>TCACAGCCACCTGCGGAAGGGTCTGCTCCA<br>TCA**TAA**	AFISAAANVTSLANYTELY<br>LEDVEVGTEYGTPSTTSAH<br>LEWWFYNVSLEYRPGEPL<br>LSQPPAEGSAPs
TMDG_GH12-2_S70A		**ATGGCGGGTTCTCATCATCATCATCATCA<br>TGGTATGGCTAGCATGACTGGTGGACAG<br>CAAATGGGTCGCTCCGGTGATGATGATG<br>ACAAG**GTCATTAAGATAAGGTACCCGGACG<br>ATGGGCAGTGGCCGGAGGCCCCAATCGACG<br>GGGACGGGGACGGGAACCCAGAGTTCTACA<br>TCGAAATAAACCCGTGGAATATACAGAGCG<br>CTGAAGGCTACGCCGAGATGACCTACAACC<br>TTAGCACAGGCGTCCTCCACTACGTCCAAG<br>CCTTAGACGATATAACCCTTAAAAACGGCG<br>GCgCGTGGGTGCACGGATATCCCGAGATATT<br>CTACGGCAACAAGCCCTGGAACAACAACTA<br>CGCTACCGATGGGGAGGTTCCACTTCCAGG<br>AAAAGTCTCGAACCTGAGCAACTTCTACCT<br>GAGCGTAAGCTACAAGCTGCTGCCAAAGAA<br>CGGCCTTCCTATCAACTTTGCAATCGAGTCG<br>TGGCTCACGAGGGAGCCCTGGAGGAACAGC<br>GGAATAAACAGCGACGAGCAGGAGCTCATG<br>ATATGGCTGTATTACGACGGACTCCAGCCG<br>GCTGGCTCAAAGGTCAAGGAAATCATTGTC<br>CCGATAGTGGTGAACGGCACCCCAGTGAAC<br>GCTACCTTCGAAGTCTGGAAGGCGAACATC<br>GGCTGGGAGTACATAGCCTTCAGGATAAAG<br>ACCCCAATAAAGGAGGGAACCGTCACTATA<br>CCGTACGGAGCCTTCATCAGCGCCGCCGCA<br>AACGTAACGAGCCTAGCTAACTACACCGAG<br>CTGTACCTGGAAGACGTTGAGGTTGGAACC<br>GAATACGGAACGCCCTCAACCACTAGCGCA<br>CACCTCGAGTGGTGGTTCTACAACGTCTCGC<br>TCGAGTACAGGCCTGGAGAGCCACTGCTCT<br>CACAGCCACCTGCGGAAGGGTCTGCTCCAT<br>CA**TAA**	**MAGSHHHHHHGMASMT<br>GGQQMGRSGDDDDK**VIK<br>IRYPDDGQWPEAPIDGDGD<br>GNPEFYIEINPWNIQSAEGY<br>AEMTYNLSTGVLHYVQAL<br>DDITLKNGGAWVHGYPEIF<br>YGNKPWNNNYATDGEVPL<br>PGKVSNLSNFYLSVSYKLL<br>PKNGLPINFAIESWLTREP<br>WRNSGINSDEQELMIWLY<br>YDGLQPAGSKVKEIIVPIVV<br>NGTPVNATFEVWKANIGW<br>EYIAFRIKTPIKEGTVTIPYG<br>AFISAAANVTSLANYTELY<br>LEDVEVGTEYGTPSTTSAH<br>LEWWFYNVSLEYRPGEPL<br>LSQPPAEGSAPs

TMDG_GH12-2_E77A		**ATGGCGGGTTCTCATCATCATCATCATCA**<br>**TGGTATGGCTAGCATGACTGGTGGACAG**<br>**CAAATGGGTCGCTCCGGTGATGATGATG**<br>**ACAAG**GTCATTAAGATAAGGTACCCGGACG<br>ATGGGCAGTGGCCGGAGGCCCCAATCGACG<br>GGGACGGGGACGGGAACCCAGAGTTCTACA<br>TCGAAATAAACCCGTGGAATATACAGAGCG<br>CTGAAGGCTACGCCGAGATGACCTACAACC<br>TTAGCACAGGCGTCCTCCACTACGTCCAAG<br>CCTTAGACGATATAACCCTTAAAAACGGCG<br>GCTCGTGGGTGCACGGATATCCCGcGATATT<br>CTACGGCAACAAGCCCTGGAACAACAACTA<br>CGCTACCGATGGGGAGGTTCCACTTCCAGG<br>AAAAGTCTCGAACCTGAGCAACTTCTACCT<br>GAGCGTAAGCTACAAGCTGCTGCCAAAGAA<br>CGGCCTTCCTATCAACTTTGCAATCGAGTCG<br>TGGCTCACGAGGGAGCCCTGGAGGAACAGC<br>GGAATAAACAGCGACGAGCAGGAGCTCATG<br>ATATGGCTGTATTACGACGGACTCCAGCCG<br>GCTGGCTCAAAGGTCAAGGAAATCATTGTC<br>CCGATAGTGGTGAACGGCACCCCAGTGAAC<br>GCTACCTTCGAAGTCTGGAAGGCGAACATC<br>GGCTGGGAGTACATAGCCTTCAGGATAAAG<br>ACCCCAATAAAGGAGGGAACCGTCACTATA<br>CCGTACGGAGCCTTCATCAGCGCCGCCGCA<br>AACGTAACGAGCCTAGCTAACTACACCGAG<br>CTGTACCTGGAAGACGTTGAGGTTGGAACC<br>GAATACGGAACGCCCTCAACCACTAGCGCA<br>CACCTCGAGTGGTGGTTCTACAACGTCTCGC<br>TCGAGTACAGGCCTGGAGAGCCACTGCTCT<br>CACAGCCACCTGCGGAAGGGTCTGCTCCAT<br>CA**TAA**	**MAGSHHHHHHGMASMT**<br>**GGQQMGRSGDDDDK**VIK<br>IRYPDDGQWPEAPIDGDGD<br>GNPEFYIEINPWNIQSAEGY<br>AEMTYNLSTGVLHYVQAL<br>DDITLKNGGSWVHGYPAIF<br>YGNKPWNNNYATDGEVPL<br>PGKVSNLSNFYLSVSYKLL<br>PKNGLPINFAIESWLTREP<br>WRNSGINSDEQELMIWLY<br>YDGLQPAGSKVKEIIVPIVV<br>NGTPVNATFEVWKANIGW<br>EYIAFRIKTPIKEGTVTIPYG<br>AFISAAANVTSLANYTELY<br>LEDVEVGTEYGTPSTTSAH<br>LEWWFYNVSLEYRPGEPL<br>LSQPPAEGSAPs
TMDG_GH12-2_N82A		**ATGGCGGGTTCTCATCATCATCATCATCA**<br>**TGGTATGGCTAGCATGACTGGTGGACAG**<br>**CAAATGGGTCGCTCCGGTGATGATGATG**<br>**ACAAG**GTCATTAAGATAAGGTACCCGGACG<br>ATGGGCAGTGGCCGGAGGCCCCAATCGACG<br>GGGACGGGGACGGGAACCCAGAGTTCTACA<br>TCGAAATAAACCCGTGGAATATACAGAGCG<br>CTGAAGGCTACGCCGAGATGACCTACAACC<br>TTAGCACAGGCGTCCTCCACTACGTCCAAG<br>CCTTAGACGATATAACCCTTAAAAACGGCG<br>GCTCGTGGGTGCACGGATATCCCGAGATAT<br>TCTACGGCgcCAAGCCCTGGAACAACAACTA<br>CGCTACCGATGGGGAGGTTCCACTTCCAGG<br>AAAAGTCTCGAACCTGAGCAACTTCTACCT<br>GAGCGTAAGCTACAAGCTGCTGCCAAAGAA<br>CGGCCTTCCTATCAACTTTGCAATCGAGTCG<br>TGGCTCACGAGGGAGCCCTGGAGGAACAGC<br>GGAATAAACAGCGACGAGCAGGAGCTCATG<br>ATATGGCTGTATTACGACGGACTCCAGCCG<br>GCTGGCTCAAAGGTCAAGGAAATCATTGTC<br>CCGATAGTGGTGAACGGCACCCCAGTGAAC<br>GCTACCTTCGAAGTCTGGAAGGCGAACATC<br>GGCTGGGAGTACATAGCCTTCAGGATAAAG<br>ACCCCAATAAAGGAGGGAACCGTCACTATA<br>CCGTACGGAGCCTTCATCAGCGCCGCCGCA<br>AACGTAACGAGCCTAGCTAACTACACCGAG<br>CTGTACCTGGAAGACGTTGAGGTTGGAACC<br>GAATACGGAACGCCCTCAACCACTAGCGCA<br>CACCTCGAGTGGTGGTTCTACAACGTCTCGC<br>TCGAGTACAGGCCTGGAGAGCCACTGCTCT	**MAGSHHHHHHGMASMT**<br>**GGQQMGRSGDDDDK**VIK<br>IRYPDDGQWPEAPIDGDGD<br>GNPEFYIEINPWNIQSAEGY<br>AEMTYNLSTGVLHYVQAL<br>DDITLKNGGSWVHGYPEIF<br>YGAKPWNNNYATDGEVPL<br>PGKVSNLSNFYLSVSYKLL<br>PKNGLPINFAIESWLTREP<br>WRNSGINSDEQELMIWLY<br>YDGLQPAGSKVKEIIVPIVV<br>NGTPVNATFEVWKANIGW<br>EYIAFRIKTPIKEGTVTIPYG<br>AFISAAANVTSLANYTELY<br>LEDVEVGTEYGTPSTTSAH<br>LEWWFYNVSLEYRPGEPL<br>LSQPPAEGSAPs

		CACAGCCACCTGCGGAAGGGTCTGCTCCAT<br>CA**TAA**	
TMDG_GH12-<br>2_K83A		**ATGGCGGGTTCTCATCATCATCATCATCA**<br>**TGGTATGGCTAGCATGACTGGTGGACAG**<br>**CAAATGGGTCGCTCCGGTGATGATGATG**<br>**ACAAG**GTCATTAAGATAAGGTACCCGGACG<br>ATGGGCAGTGGCCGGAGGCCCCAATCGACG<br>GGGACGGGGACGGGAACCCAGAGTTCTACA<br>TCGAAATAAACCCGTGGAATATACAGAGCG<br>CTGAAGGCTACGCCGAGATGACCTACAACC<br>TTAGCACAGGCGTCCTCCACTACGTCCAAG<br>CCTTAGACGATATAACCCTTAAAAACGGCG<br>GCTCGTGGGTGCACGGATATCCCGAGATAT<br>TCTACGGCAACgcGCCCTGGAACAACAACTA<br>CGCTACCGATGGGGAGGTTCCACTTCCAGG<br>AAAAGTCTCGAACCTGAGCAACTTCTACCT<br>GAGCGTAAGCTACAAGCTGCTGCCAAAGAA<br>CGGCCTTCCTATCAACTTTGCAATCGAGTCG<br>TGGCTCACGAGGGAGCCCTGGAGGAACAGC<br>GGAATAAACAGCGACGAGCAGGAGCTCATG<br>ATATGGCTGTATTACGACGGACTCCAGCCG<br>GCTGGCTCAAAGGTCAAGGAAATCATTGTC<br>CCGATAGTGGTGAACGGCACCCCAGTGAAC<br>GCTACCTTCGAAGTCTGGAAGGCGAACATC<br>GGCTGGGAGTACATAGCCTTCAGGATAAAG<br>ACCCCAATAAAGGAGGGAACCGTCACTATA<br>CCGTACGGAGCCTTCATCAGCGCCGCCGCA<br>AACGTAACGAGCCTAGCTAACTACACCGAG<br>CTGTACCTGGAAGACGTTGAGGTTGGAACC<br>GAATACGGAACGCCCTCAACCACTAGCGCA<br>CACCTCGAGTGGTGGTTCTACAACGTCTCGC<br>TCGAGTACAGGCCTGGAGAGCCACTGCTCT<br>CACAGCCACCTGCGGAAGGGTCTGCTCCAT<br>CA**TAA**	**MAGSHHHHHHGMASMT**<br>**GGQQMGRSGDDDDK**VIK<br>IRYPDDGQWPEAPIDGDGD<br>GNPEFYIEINPWNIQSAEGY<br>AEMTYNLSTGVLHYVQAL<br>DDITLKNGGSWVHGYPEIF<br>YGNAPWNNNYATDGEVPL<br>PGKVSNLSNFYLSVSYKLL<br>PKNGLPINFAIESWLTREP<br>WRNSGINSDEQELMIWLY<br>YDGLQPAGSKVKEIIVPIVV<br>NGTPVNATFEVWKANIGW<br>EYIAFRIKTPIKEGTVTIPYG<br>AFISAAANVTSLANYTELY<br>LEDVEVGTEYGTPSTTSAH<br>LEWWFYNVSLEYRPGEPL<br>LSQPPAEGSAPs
TMDG_GH12-<br>2_N86A		**ATGGCGGGTTCTCATCATCATCATCATCA**<br>**TGGTATGGCTAGCATGACTGGTGGACAG**<br>**CAAATGGGTCGCTCCGGTGATGATGATG**<br>**ACAAG**GTCATTAAGATAAGGTACCCGGACG<br>ATGGGCAGTGGCCGGAGGCCCCAATCGACG<br>GGGACGGGGACGGGAACCCAGAGTTCTACA<br>TCGAAATAAACCCGTGGAATATACAGAGCG<br>CTGAAGGCTACGCCGAGATGACCTACAACC<br>TTAGCACAGGCGTCCTCCACTACGTCCAAG<br>CCTTAGACGATATAACCCTTAAAAACGGCG<br>GCTCGTGGGTGCACGGATATCCCGAGATAT<br>TCTACGGCAACAAGCCCTGGgcCAACAACTA<br>CGCTACCGATGGGGAGGTTCCACTTCCAGG<br>AAAAGTCTCGAACCTGAGCAACTTCTACCT<br>GAGCGTAAGCTACAAGCTGCTGCCAAAGAA	**MAGSHHHHHHGMASMT**<br>**GGQQMGRSGDDDDK**VIK<br>IRYPDDGQWPEAPIDGDGD<br>GNPEFYIEINPWNIQSAEGY<br>AEMTYNLSTGVLHYVQAL<br>DDITLKNGGSWVHGYPEIF<br>YGNKPWANNYATDGEVPL<br>PGKVSNLSNFYLSVSYKLL<br>PKNGLPINFAIESWLTREP<br>WRNSGINSDEQELMIWLY<br>YDGLQPAGSKVKEIIVPIVV<br>NGTPVNATFEVWKANIGW<br>EYIAFRIKTPIKEGTVTIPYG<br>AFISAAANVTSLANYTELY<br>LEDVEVGTEYGTPSTTSAH

		CGGCCTTCCTATCAACTTTGCAATCGAGTCG<br>TGGCTCACGAGGGAGCCCTGGAGGAACAGC<br>GGAATAAACAGCGACGAGCAGGAGCTCATG<br>ATATGGCTGTATTACGACGGACTCCAGCCG<br>GCTGGCTCAAAGGTCAAGGAAATCATTGTC<br>CCGATAGTGGTGAACGGCACCCCAGTGAAC<br>GCTACCTTCGAAGTCTGGAAGGCGAACATC<br>GGCTGGGAGTACATAGCCTTCAGGATAAAG<br>ACCCCAATAAAGGAGGGAACCGTCACTATA<br>CCGTACGGAGCCTTCATCAGCGCCGCCGCA<br>AACGTAACGAGCCTAGCTAACTACACCGAG<br>CTGTACCTGGAAGACGTTGAGGTTGGAACC<br>GAATACGGAACGCCCTCAACCACTAGCGCA<br>CACCTCGAGTGGTGGTTCTACAACGTCTCGC<br>TCGAGTACAGGCCTGGAGAGCCACTGCTCT<br>CACAGCCACCTGCGGAAGGGTCTGCTCCAT<br>CA**TAA**	LEWWFYNVSLEYRPGEPL<br>LSQPPAEGSAPs
TMDG_GH12-2_N87A		**ATGGCGGGTTCTCATCATCATCATCATCA**<br>**TGGTATGGCTAGCATGACTGGTGGACAG**<br>**CAAATGGGTCGCTCCGGTGATGATGATG**<br>**ACAAG**GTCATTAAGATAAGGTACCCGGACG<br>ATGGGCAGTGGCCGGAGGCCCCAATCGACG<br>GGGACGGGGACGGGAACCCAGAGTTCTACA<br>TCGAAATAAACCCGTGGAATATACAGAGCG<br>CTGAAGGCTACGCCGAGATGACCTACAACC<br>TTAGCACAGGCGTCCTCCACTACGTCCAAG<br>CCTTAGACGATATAACCCTTAAAAACGGCG<br>GCTCGTGGGTGCACGGATATCCCGAGATAT<br>TCTACGGCAACAAGCCCTGGAACgcCAACTA<br>CGCTACCGATGGGGAGGTTCCACTTCCAGG<br>AAAAGTCTCGAACCTGAGCAACTTCTACCT<br>GAGCGTAAGCTACAAGCTGCTGCCAAAGAA<br>CGGCCTTCCTATCAACTTTGCAATCGAGTCG<br>TGGCTCACGAGGGAGCCCTGGAGGAACAGC<br>GGAATAAACAGCGACGAGCAGGAGCTCATG<br>ATATGGCTGTATTACGACGGACTCCAGCCG<br>GCTGGCTCAAAGGTCAAGGAAATCATTGTC<br>CCGATAGTGGTGAACGGCACCCCAGTGAAC<br>GCTACCTTCGAAGTCTGGAAGGCGAACATC<br>GGCTGGGAGTACATAGCCTTCAGGATAAAG<br>ACCCCAATAAAGGAGGGAACCGTCACTATA<br>CCGTACGGAGCCTTCATCAGCGCCGCCGCA<br>AACGTAACGAGCCTAGCTAACTACACCGAG<br>CTGTACCTGGAAGACGTTGAGGTTGGAACC<br>GAATACGGAACGCCCTCAACCACTAGCGCA<br>CACCTCGAGTGGTGGTTCTACAACGTCTCGC<br>TCGAGTACAGGCCTGGAGAGCCACTGCTCT<br>CACAGCCACCTGCGGAAGGGTCTGCTCCAT<br>CA**TAA**	**MAGSHHHHHHGMASMT**<br>**GGQQMGRSGDDDDK**VIK<br>IRYPDDGQWPEAPIDGDGD<br>GNPEFYIEINPWNIQSAEGY<br>AEMTYNLSTGVLHYVQAL<br>DDITLKNGGSWVHGYPEIF<br>YGNKPWNANYATDGEVPL<br>PGKVSNLSNFYLSVSYKLL<br>PKNGLPINFAIESWLTREP<br>WRNSGINSDEQELMIWLY<br>YDGLQPAGSKVKEIIVPIVV<br>NGTPVNATFEVWKANIGW<br>EYIAFRIKTPIKEGTVTIPYG<br>AFISAAANVTSLANYTELY<br>LEDVEVGTEYGTPSTTSAH<br>LEWWFYNVSLEYRPGEPL<br>LSQPPAEGSAPs

TMDG_GH12-2_N88A		**ATGGCGGGTTCTCATCATCATCATCATCA<br>TGGTATGGCTAGCATGACTGGTGGACAG<br>CAAATGGGTCGCTCCGGTGATGATGATG<br>ACAAG**GTCATTAAGATAAGGTACCCGGACG<br>ATGGGCAGTGGCCGGAGGCCCCAATCGACG<br>GGGACGGGGACGGGAACCCAGAGTTCTACA<br>TCGAAATAAACCCGTGGAATATACAGAGCG<br>CTGAAGGCTACGCCGAGATGACCTACAACC<br>TTAGCACAGGCGTCCTCCACTACGTCCAAG<br>CCTTAGACGATATAACCCTTAAAAACGGCG<br>GCTCGTGGGTGCACGGATATCCCGAGATAT<br>TCTACGGCAACAAGCCCTGGAACAACgcCTA<br>CGCTACCGATGGGGAGGTTCCACTTCCAGG<br>AAAAGTCTCGAACCTGAGCAACTTCTACCT<br>GAGCGTAAGCTACAAGCTGCTGCCAAAGAA<br>CGGCCTTCCTATCAACTTTGCAATCGAGTCG<br>TGGCTCACGAGGGAGCCCTGGAGGAACAGC<br>GGAATAAACAGCGACGAGCAGGAGCTCATG<br>ATATGGCTGTATTACGACGGACTCCAGCCG<br>GCTGGCTCAAAGGTCAAGGAAATCATTGTC<br>CCGATAGTGGTGAACGGCACCCCAGTGAAC<br>GCTACCTTCGAAGTCTGGAAGGCGAACATC<br>GGCTGGGAGTACATAGCCTTCAGGATAAAG<br>ACCCCAATAAAGGAGGGAACCGTCACTATA<br>CCGTACGGAGCCTTCATCAGCGCCGCCGCA<br>AACGTAACGAGCCTAGCTAACTACACCGAG<br>CTGTACCTGGAAGACGTTGAGGTTGGAACC<br>GAATACGGAACGCCCTCAACCACTAGCGCA<br>CACCTCGAGTGGTGGTTCTACAACGTCTCGC<br>TCGAGTACAGGCCTGGAGAGCCACTGCTCT<br>CACAGCCACCTGCGGAAGGGTCTGCTCCAT<br>CA**TAA**	**MAGSHHHHHHGMASMT<br>GGQQMGRSGDDDDK**VIK<br>IRYPDDGQWPEAPIDGDGD<br>GNPEFYIEINPWNIQSAEGY<br>AEMTYNLSTGVLHYVQAL<br>DDITLKNGGSWVHGYPEIF<br>YGNKPWNNAYATDGEVPL<br>PGKVSNLSNFYLSVSYKLL<br>PKNGLPINFAIESWLTREP<br>WRNSGINSDEQELMIWLY<br>YDGLQPAGSKVKEIIVPIVV<br>NGTPVNATFEVWKANIGW<br>EYIAFRIKTPIKEGTVTIPYG<br>AFISAAANVTSLANYTELY<br>LEDVEVGTEYGTPSTTSAH<br>LEWWFYNVSLEYRPGEPL<br>LSQPPAEGSAPs
TMDG_GH12-2_D92A		**ATGGCGGGTTCTCATCATCATCATCATCA<br>TGGTATGGCTAGCATGACTGGTGGACAG<br>CAAATGGGTCGCTCCGGTGATGATGATG<br>ACAAG**GTCATTAAGATAAGGTACCCGGACG<br>ATGGGCAGTGGCCGGAGGCCCCAATCGACG<br>GGGACGGGGACGGGAACCCAGAGTTCTACA<br>TCGAAATAAACCCGTGGAATATACAGAGCG<br>CTGAAGGCTACGCCGAGATGACCTACAACC<br>TTAGCACAGGCGTCCTCCACTACGTCCAAG<br>CCTTAGACGATATAACCCTTAAAAACGGCG<br>GCTCGTGGGTGCACGGATATCCCGAGATAT<br>TCTACGGCAACAAGCCCTGGAACAACAACT<br>ACGCTACCGcTGGGGAGGTTCCACTTCCAGG<br>AAAAGTCTCGAACCTGAGCAACTTCTACCT<br>GAGCGTAAGCTACAAGCTGCTGCCAAAGAA<br>CGGCCTTCCTATCAACTTTGCAATCGAGTCG<br>TGGCTCACGAGGGAGCCCTGGAGGAACAGC<br>GGAATAAACAGCGACGAGCAGGAGCTCATG<br>ATATGGCTGTATTACGACGGACTCCAGCCG<br>GCTGGCTCAAAGGTCAAGGAAATCATTGTC<br>CCGATAGTGGTGAACGGCACCCCAGTGAAC<br>GCTACCTTCGAAGTCTGGAAGGCGAACATC<br>GGCTGGGAGTACATAGCCTTCAGGATAAAG<br>ACCCCAATAAAGGAGGGAACCGTCACTATA<br>CCGTACGGAGCCTTCATCAGCGCCGCCGCA<br>AACGTAACGAGCCTAGCTAACTACACCGAG<br>CTGTACCTGGAAGACGTTGAGGTTGGAACC<br>GAATACGGAACGCCCTCAACCACTAGCGCA<br>CACCTCGAGTGGTGGTTCTACAACGTCTCGC<br>TCGAGTACAGGCCTGGAGAGCCACTGCTCT	**MAGSHHHHHHGMASMT<br>GGQQMGRSGDDDDK**VIK<br>IRYPDDGQWPEAPIDGDGD<br>GNPEFYIEINPWNIQSAEGY<br>AEMTYNLSTGVLHYVQAL<br>DDITLKNGGSWVHGYPEIF<br>YGNKPWNNNYATAGEVPL<br>PGKVSNLSNFYLSVSYKLL<br>PKNGLPINFAIESWLTREP<br>WRNSGINSDEQELMIWLY<br>YDGLQPAGSKVKEIIVPIVV<br>NGTPVNATFEVWKANIGW<br>EYIAFRIKTPIKEGTVTIPYG<br>AFISAAANVTSLANYTELY<br>LEDVEVGTEYGTPSTTSAH<br>LEWWFYNVSLEYRPGEPL<br>LSQPPAEGSAPS

		CACAGCCACCTGCGGAAGGGTCTGCTCCAT<br>CA**TAA**	
TMDG_GH12-2_W130A		**ATGGCGGGTTCTCATCATCATCATCATCA**<br>**TGGTATGGCTAGCATGACTGGTGGACAG**<br>**CAAATGGGTCGCTCCGGTGATGATGATG**<br>**ACAAG**GTCATTAAGATAAGGTACCCGGACG<br>ATGGGCAGTGGCCGGAGGCCCCAATCGACG<br>GGGACGGGGACGGGAACCCAGAGTTCTACA<br>TCGAAATAAACCCGTGGAATATACAGAGCG<br>CTGAAGGCTACGCCGAGATGACCTACAACC<br>TTAGCACAGGCGTCCTCCACTACGTCCAAG<br>CCTTAGACGATATAACCCTTAAAAACGGCG<br>GCTCGTGGGTGCACGGATATCCCGAGATAT<br>TCTACGGCAACAAGCCCTGGAACAACAACT<br>ACGCTACCGATGGGGAGGTTCCACTTCCAG<br>GAAAAGTCTCGAACCTGAGCAACTTCTACC<br>TGAGCGTAAGCTACAAGCTGCTGCCAAAGA<br>ACGGCCTTCCTATCAACTTTGCAATCGAGTC<br>GgcGCTCACGAGGGAGCCCTGGAGGAACAG<br>CGGAATAAACAGCGACGAGCAGGAGCTCAT<br>GATATGGCTGTATTACGACGGACTCCAGCC<br>GGCTGGCTCAAAGGTCAAGGAAATCATTGT<br>CCCGATAGTGGTGAACGGCACCCCAGTGAA<br>CGCTACCTTCGAAGTCTGGAAGGCGAACAT<br>CGGCTGGGAGTACATAGCCTTCAGGATAAA<br>GACCCCAATAAAGGAGGGAACCGTCACTAT<br>ACCGTACGGAGCCTTCATCAGCGCCGCCGC<br>AAACGTAACGAGCCTAGCTAACTACACCGA<br>GCTGTACCTGGAAGACGTTGAGGTTGGAAC<br>CGAATACGGAACGCCCTCAACCACTAGCGC<br>ACACCTCGAGTGGTGGTTCTACAACGTCTCG<br>CTCGAGTACAGGCCTGGAGAGCCACTGCTC<br>TCACAGCCACCTGCGGAAGGGTCTGCTCCA<br>TCA**TAA**	**MAGSHHHHHHGMASMT**<br>**GGQQMGRSGDDDDK**VIK<br>IRYPDDGQWPEAPIDGDGD<br>GNPEFYIEINPWNIQSAEGY<br>AEMTYNLSTGVLHYVQAL<br>DDITLKNGGSWVHGYPEIF<br>YGNKPWNNNYATDGEVPL<br>PGKVSNLSNFYLSVSYKLL<br>PKNGLPINFAIESALTREPW<br>RNSGINSDEQELMIWLYYD<br>GLQPAGSKVKEIIVPIVVNG<br>TPVNATFEVWKANIGWEY<br>IAFRIKTPIKEGTVTIPYGAF<br>ISAAANVTSLANYTELYLE<br>DVEVGTEYGTPSTTSAHLE<br>WWFYNVSLEYRPGEPLLS<br>QPPAEGSAPs
TMDG_GH12-2_N138A		**ATGGCGGGTTCTCATCATCATCATCATCA**<br>**TGGTATGGCTAGCATGACTGGTGGACAG**<br>**CAAATGGGTCGCTCCGGTGATGATGATG**<br>**ACAAG**GTCATTAAGATAAGGTACCCGGACG<br>ATGGGCAGTGGCCGGAGGCCCCAATCGACG<br>GGGACGGGGACGGGAACCCAGAGTTCTACA<br>TCGAAATAAACCCGTGGAATATACAGAGCG<br>CTGAAGGCTACGCCGAGATGACCTACAACC<br>TTAGCACAGGCGTCCTCCACTACGTCCAAG<br>CCTTAGACGATATAACCCTTAAAAACGGCG<br>GCTCGTGGGTGCACGGATATCCCGAGATAT<br>TCTACGGCAACAAGCCCTGGAACAACAACT<br>ACGCTACCGATGGGGAGGTTCCACTTCCAG<br>GAAAAGTCTCGAACCTGAGCAACTTCTACC<br>TGAGCGTAAGCTACAAGCTGCTGCCAAAGA<br>ACGGCCTTCCTATCAACTTTGCAATCGAGTC<br>GTGGCTCACGAGGGAGCCCTGGAGGgcCAG<br>CGGAATAAACAGCGACGAGCAGGAGCTCAT<br>GATATGGCTGTATTACGACGGACTCCAGCC<br>GGCTGGCTCAAAGGTCAAGGAAATCATTGT<br>CCCGATAGTGGTGAACGGCACCCCAGTGAA	**MAGSHHHHHHGMASMT**<br>**GGQQMGRSGDDDDK**VIK<br>IRYPDDGQWPEAPIDGDGD<br>GNPEFYIEINPWNIQSAEGY<br>AEMTYNLSTGVLHYVQAL<br>DDITLKNGGSWVHGYPEIF<br>YGNKPWNNNYATDGEVPL<br>PGKVSNLSNFYLSVSYKLL<br>PKNGLPINFAIESWLTREP<br>WRASGINSDEQELMIWLY<br>YDGLQPAGSKVKEIIVPIVV<br>NGTPVNATFEVWKANIGW<br>EYIAFRIKTPIKEGTVTIPYG<br>AFISAAANVTSLANYTELY<br>LEDVEVGTEYGTPSTTSAH<br>LEWWFYNVSLEYRPGEPL<br>LSQPPAEGSAPs

		CGCTACCTTCGAAGTCTGGAAGGCGAACAT<br>CGGCTGGGAGTACATAGCCTTCAGGATAAA<br>GACCCCAATAAAGGAGGGAACCGTCACTAT<br>ACCGTACGGAGCCTTCATCAGCGCCGCCGC<br>AAACGTAACGAGCCTAGCTAACTACACCGA<br>GCTGTACCTGGAAGACGTTGAGGTTGGAAC<br>CGAATACGGAACGCCCTCAACCACTAGCGC<br>ACACCTCGAGTGGTGGTTCTACAACGTCTCG<br>CTCGAGTACAGGCCTGGAGAGCCACTGCTC<br>TCACAGCCACCTGCGGAAGGGTCTGCTCCA<br>TCA**TAA**	
TMDG_GH12-2_E147A		**ATGGCGGGTTCTCATCATCATCATCATCA**<br>**TGGTATGGCTAGCATGACTGGTGGACAG**<br>**CAAATGGGTCGCTCCGGTGATGATGATG**<br>**ACAAG**GTCATTAAGATAAGGTACCCGGACG<br>ATGGGCAGTGGCCGGAGGCCCCAATCGACG<br>GGGACGGGGACGGGAACCCAGAGTTCTACA<br>TCGAAATAAACCCGTGGAATATACAGAGCG<br>CTGAAGGCTACGCCGAGATGACCTACAACC<br>TTAGCACAGGCGTCCTCCACTACGTCCAAG<br>CCTTAGACGATATAACCCTTAAAAACGGCG<br>GCTCGTGGGTGCACGGATATCCCGAGATAT<br>TCTACGGCAACAAGCCCTGGAACAACAACT<br>ACGCTACCGATGGGGAGGTTCCACTTCCAG<br>GAAAAGTCTCGAACCTGAGCAACTTCTACC<br>TGAGCGTAAGCTACAAGCTGCTGCCAAAGA<br>ACGGCCTTCCTATCAACTTTGCAATCGAGTC<br>GTGGCTCACGAGGGAGCCCTGGAGGAACAG<br>CGGAATAAACAGCGACGAGCAGGcGCTCAT<br>GATATGGCTGTATTACGACGGACTCCAGCC<br>GGCTGGCTCAAAGGTCAAGGAAATCATTGT<br>CCCGATAGTGGTGAACGGCACCCCAGTGAA<br>CGCTACCTTCGAAGTCTGGAAGGCGAACAT<br>CGGCTGGGAGTACATAGCCTTCAGGATAAA<br>GACCCCAATAAAGGAGGGAACCGTCACTAT<br>ACCGTACGGAGCCTTCATCAGCGCCGCCGC<br>AAACGTAACGAGCCTAGCTAACTACACCGA<br>GCTGTACCTGGAAGACGTTGAGGTTGGAAC<br>CGAATACGGAACGCCCTCAACCACTAGCGC<br>ACACCTCGAGTGGTGGTTCTACAACGTCTCG<br>CTCGAGTACAGGCCTGGAGAGCCACTGCTC<br>TCACAGCCACCTGCGGAAGGGTCTGCTCCA<br>TCA**TAA**	**MAGSHHHHHHGMASMT**<br>**GGQQMGRSGDDDDK**VIK<br>IRYPDDGQWPEAPIDGDGD<br>GNPEFYIEINPWNIQSAEGY<br>AEMTYNLSTGVLHYVQAL<br>DDITLKNGGSWVHGYPEIF<br>YGNKPWNNNYATDGEVPL<br>PGKVSNLSNFYLSVSYKLL<br>PKNGLPINFAIESWLTREP<br>WRNSGINSDEQALMIWLY<br>YDGLQPAGSKVKEIIVPIVV<br>NGTPVNATFEVWKANIGW<br>EYIAFRIKTPIKEGTVTIPYG<br>AFISAAANVTSLANYTELY<br>LEDVEVGTEYGTPSTTSAH<br>LEWWFYNVSLEYRPGEPL<br>LSQPPAEGSAPs
TMDG_GH12-2_M149A		**ATGGCGGGTTCTCATCATCATCATCATCA**<br>**TGGTATGGCTAGCATGACTGGTGGACAG**<br>**CAAATGGGTCGCTCCGGTGATGATGATG**<br>**ACAAG**GTCATTAAGATAAGGTACCCGGACG<br>ATGGGCAGTGGCCGGAGGCCCCAATCGACG<br>GGGACGGGGACGGGAACCCAGAGTTCTACA<br>TCGAAATAAACCCGTGGAATATACAGAGCG<br>CTGAAGGCTACGCCGAGATGACCTACAACC<br>TTAGCACAGGCGTCCTCCACTACGTCCAAG<br>CCTTAGACGATATAACCCTTAAAAACGGCG<br>GCTCGTGGGTGCACGGATATCCCGAGATAT<br>TCTACGGCAACAAGCCCTGGAACAACAACT<br>ACGCTACCGATGGGGAGGTTCCACTTCCAG<br>GAAAAGTCTCGAACCTGAGCAACTTCTACC<br>TGAGCGTAAGCTACAAGCTGCTGCCAAAGA<br>ACGGCCTTCCTATCAACTTTGCAATCGAGTC<br>GTGGCTCACGAGGGAGCCCTGGAGGAACAG<br>CGGAATAAACAGCGACGAGCAGGAGCTCgc<br>GATATGGCTGTATTACGACGGACTCCAGCC<br>GGCTGGCTCAAAGGTCAAGGAAATCATTGT	**MAGSHHHHHHGMASMT**<br>**GGQQMGRSGDDDDK**VIK<br>IRYPDDGQWPEAPIDGDGD<br>GNPEFYIEINPWNIQSAEGY<br>AEMTYNLSTGVLHYVQAL<br>DDITLKNGGSWVHGYPEIF<br>YGNKPWNNNYATDGEVPL<br>PGKVSNLSNFYLSVSYKLL<br>PKNGLPINFAIESWLTREP<br>WRNSGINSDEQELAIWLYY<br>DGLQPAGSKVKEIIVPIVVN<br>GTPVNATFEVWKANIGWE<br>YIAFRIKTPIKEGTVTIPYG<br>AFISAAANVTSLANYTELY<br>LEDVEVGTEYGTPSTTSAH<br>LEWWFYNVSLEYRPGEPL<br>LSQPPAEGSAPs

		CCCGATAGTGGTGAACGGCACCCCAGTGAA<br>CGCTACCTTCGAAGTCTGGAAGGCGAACAT<br>CGGCTGGGAGTACATAGCCTTCAGGATAAA<br>GACCCCAATAAAGGAGGGAACCGTCACTAT<br>ACCGTACGGAGCCTTCATCAGCGCCGCCGC<br>AAACGTAACGAGCCTAGCTAACTACACCGA<br>GCTGTACCTGGAAGACGTTGAGGTTGGAAC<br>CGAATACGGAACGCCCTCAACCACTAGCGC<br>ACACCTCGAGTGGTGGTTCTACAACGTCTCG<br>CTCGAGTACAGGCCTGGAGAGCCACTGCTC<br>TCACAGCCACCTGCGGAAGGGTCTGCTCCA<br>TCA**TAA**	
TMDG_GH12-2_W151A		**ATGGCGGGTTCTCATCATCATCATCATCA**<br>**TGGTATGGCTAGCATGACTGGTGGACAG**<br>**CAAATGGGTCGCTCCGGTGATGATGATG**<br>**ACAAG**GTCATTAAGATAAGGTACCCGGACG<br>ATGGGCAGTGGCCGGAGGCCCCAATCGACG<br>GGGACGGGGACGGGAACCCAGAGTTCTACA<br>TCGAAATAAACCCGTGGAATATACAGAGCG<br>CTGAAGGCTACGCCGAGATGACCTACAACC<br>TTAGCACAGGCGTCCTCCACTACGTCCAAG<br>CCTTAGACGATATAACCCTTAAAAACGGCG<br>GCTCGTGGGTGCACGGATATCCCGAGATAT<br>TCTACGGCAACAAGCCCTGGAACAACAACT<br>ACGCTACCGATGGGGAGGTTCCACTTCCAG<br>GAAAAGTCTCGAACCTGAGCAACTTCTACC<br>TGAGCGTAAGCTACAAGCTGCTGCCAAAGA<br>ACGGCCTTCCTATCAACTTTGCAATCGAGTC<br>GTGGCTCACGAGGGAGCCCTGGAGGAACAG<br>CGGAATAAACAGCGACGAGCAGGAGCTCAT<br>GATAgcGCTGTATTACGACGGACTCCAGCCG<br>GCTGGCTCAAAGGTCAAGGAAATCATTGTC<br>CCGATAGTGGTGAACGGCACCCCAGTGAAC<br>GCTACCTTCGAAGTCTGGAAGGCGAACATC<br>GGCTGGGAGTACATAGCCTTCAGGATAAAG<br>ACCCCAATAAAGGAGGGAACCGTCACTATA<br>CCGTACGGAGCCTTCATCAGCGCCGCCGCA<br>AACGTAACGAGCCTAGCTAACTACACCGAG<br>CTGTACCTGGAAGACGTTGAGGTTGGAACC<br>GAATACGGAACGCCCTCAACCACTAGCGCA<br>CACCTCGAGTGGTGGTTCTACAACGTCTCGC<br>TCGAGTACAGGCCTGGAGAGCCACTGCTCT<br>CACAGCCACCTGCGGAAGGGTCTGCTCCAT<br>CA**TAA**	**MAGSHHHHHHGMASMT**<br>**GGQQMGRSGDDDDK**VIK<br>IRYPDDGQWPEAPIDGDGD<br>GNPEFYIEINPWNIQSAEGY<br>AEMTYNLSTGVLHYVQAL<br>DDITLKNGGSWVHGYPEIF<br>YGNKPWNNNYATDGEVPL<br>PGKVSNLSNFYLSVSYKLL<br>PKNGLPINFAIESWLTREP<br>WRNSGINSDEQELMIALYY<br>DGLQPAGSKVKEIIVPIVVN<br>GTPVNATFEVWKANIGWE<br>YIAFRIKTPIKEGTVTIPYG<br>AFISAAANVTSLANYTELY<br>LEDVEVGTEYGTPSTTSAH<br>LEWWFYNVSLEYRPGEPL<br>LSQPPAEGSAPs
TMDG_GH12-2_E233A		**ATGGCGGGTTCTCATCATCATCATCATCA**<br>**TGGTATGGCTAGCATGACTGGTGGACAG**<br>**CAAATGGGTCGCTCCGGTGATGATGATG**<br>**ACAAG**GTCATTAAGATAAGGTACCCGGACG<br>ATGGGCAGTGGCCGGAGGCCCCAATCGACG<br>GGGACGGGGACGGGAACCCAGAGTTCTACA<br>TCGAAATAAACCCGTGGAATATACAGAGCG<br>CTGAAGGCTACGCCGAGATGACCTACAACC<br>TTAGCACAGGCGTCCTCCACTACGTCCAAG<br>CCTTAGACGATATAACCCTTAAAAACGGCG<br>GCTCGTGGGTGCACGGATATCCCGAGATAT<br>TCTACGGCAACAAGCCCTGGAACAACAACT<br>ACGCTACCGATGGGGAGGTTCCACTTCCAG<br>GAAAAGTCTCGAACCTGAGCAACTTCTACC<br>TGAGCGTAAGCTACAAGCTGCTGCCAAAGA<br>ACGGCCTTCCTATCAACTTTGCAATCGAGTC<br>GTGGCTCACGAGGGAGCCCTGGAGGAACAG<br>CGGAATAAACAGCGACGAGCAGGAGCTCAT<br>GATATGGCTGTATTACGACGGACTCCAGCC	**MAGSHHHHHHGMASMT**<br>**GGQQMGRSGDDDDK**VIK<br>IRYPDDGQWPEAPIDGDGD<br>GNPEFYIEINPWNIQSAEGY<br>AEMTYNLSTGVLHYVQAL<br>DDITLKNGGSWVHGYPEIF<br>YGNKPWNNNYATDGEVPL<br>PGKVSNLSNFYLSVSYKLL<br>PKNGLPINFAIESWLTREP<br>WRNSGINSDEQELMIWLY<br>YDGLQPAGSKVKEIIVPIVV<br>NGTPVNATFEVWKANIGW<br>EYIAFRIKTPIKEGTVTIPYG<br>AFISAAANVTSLANYTELY<br>LADVEVGTEYGTPSTTSAH<br>LEWWFYNVSLEYRPGEPL<br>LSQPPAEGSAPs

		GGCTGGCTCAAAGGTCAAGGAAATCATTGT<br>CCCGATAGTGGTGAACGGCACCCCAGTGAA<br>CGCTACCTTCGAAGTCTGGAAGGCGAACAT<br>CGGCTGGGAGTACATAGCCTTCAGGATAAA<br>GACCCCAATAAAGGAGGGAACCGTCACTAT<br>ACCGTACGGAGCCTTCATCAGCGCCGCCGC<br>AAACGTAACGAGCCTAGCTAACTACACCGA<br>GCTGTACCTGGcAGACGTTGAGGTTGGAACC<br>GAATACGGAACGCCCTCAACCACTAGCGCA<br>CACCTCGAGTGGTGGTTCTACAACGTCTCGC<br>TCGAGTACAGGCCTGGAGAGCCACTGCTCT<br>CACAGCCACCTGCGGAAGGGTCTGCTCCAT<br>CA**TAA**	
TMDG_GH12-2_E236A		**ATGGCGGGTTCTCATCATCATCATCATCA**<br>**TGGTATGGCTAGCATGACTGGTGGACAG**<br>**CAAATGGGTCGCTCCGGTGATGATGATG**<br>**ACAAG**GTCATTAAGATAAGGTACCCGGACG<br>ATGGGCAGTGGCCGGAGGCCCCAATCGACG<br>GGGACGGGGACGGGAACCCAGAGTTCTACA<br>TCGAAATAAACCCGTGGAATATACAGAGCG<br>CTGAAGGCTACGCCGAGATGACCTACAACC<br>TTAGCACAGGCGTCCTCCACTACGTCCAAG<br>CCTTAGACGATATAACCCTTAAAAACGGCG<br>GCTCGTGGGTGCACGGATATCCCGAGATAT<br>TCTACGGCAACAAGCCCTGGAACAACAACT<br>ACGCTACCGATGGGGAGGTTCCACTTCCAG<br>GAAAAGTCTCGAACCTGAGCAACTTCTACC<br>TGAGCGTAAGCTACAAGCTGCTGCCAAAGA<br>ACGGCCTTCCTATCAACTTTGCAATCGAGTC<br>GTGGCTCACGAGGGAGCCCTGGAGGAACAG<br>CGGAATAAACAGCGACGAGCAGGAGCTCAT<br>GATATGGCTGTATTACGACGGACTCCAGCC<br>GGCTGGCTCAAAGGTCAAGGAAATCATTGT<br>CCCGATAGTGGTGAACGGCACCCCAGTGAA<br>CGCTACCTTCGAAGTCTGGAAGGCGAACAT<br>CGGCTGGGAGTACATAGCCTTCAGGATAAA<br>GACCCCAATAAAGGAGGGAACCGTCACTAT<br>ACCGTACGGAGCCTTCATCAGCGCCGCCGC<br>AAACGTAACGAGCCTAGCTAACTACACCGA<br>GCTGTACCTGGAAGACGTTGcGGTTGGAACC<br>GAATACGGAACGCCCTCAACCACTAGCGCA<br>CACCTCGAGTGGTGGTTCTACAACGTCTCGC<br>TCGAGTACAGGCCTGGAGAGCCACTGCTCT<br>CACAGCCACCTGCGGAAGGGTCTGCTCCAT<br>CA**TAA**	**MAGSHHHHHHGMASMT**<br>**GGQQMGRSGDDDDK**VIK<br>IRYPDDGQWPEAPIDGDGD<br>GNPEFYIEINPWNIQSAEGY<br>AEMTYNLSTGVLHYVQAL<br>DDITLKNGGSWVHGYPEIF<br>YGNKPWNNNYATDGEVPL<br>PGKVSNLSNFYLSVSYKLL<br>PKNGLPINFAIESWLTREP<br>WRNSGINSDEQELMIWLY<br>YDGLQPAGSKVKEIIVPIVV<br>NGTPVNATFEVWKANIGW<br>EYIAFRIKTPIKEGTVTIPYG<br>AFISAAANVTSLANYTELY<br>LEDVAVGTEYGTPSTTSAH<br>LEWWFYNVSLEYRPGEPL<br>LSQPPAEGSAPs
TMDG_GH12-2_E240A		**ATGGCGGGTTCTCATCATCATCATCATCA**<br>**TGGTATGGCTAGCATGACTGGTGGACAG**<br>**CAAATGGGTCGCTCCGGTGATGATGATG**<br>**ACAAG**GTCATTAAGATAAGGTACCCGGACG<br>ATGGGCAGTGGCCGGAGGCCCCAATCGACG<br>GGGACGGGGACGGGAACCCAGAGTTCTACA<br>TCGAAATAAACCCGTGGAATATACAGAGCG<br>CTGAAGGCTACGCCGAGATGACCTACAACC<br>TTAGCACAGGCGTCCTCCACTACGTCCAAG<br>CCTTAGACGATATAACCCTTAAAAACGGCG<br>GCTCGTGGGTGCACGGATATCCCGAGATAT<br>TCTACGGCAACAAGCCCTGGAACAACAACT<br>ACGCTACCGATGGGGAGGTTCCACTTCCAG<br>GAAAAGTCTCGAACCTGAGCAACTTCTACC<br>TGAGCGTAAGCTACAAGCTGCTGCCAAAGA<br>ACGGCCTTCCTATCAACTTTGCAATCGAGTC<br>GTGGCTCACGAGGGAGCCCTGGAGGAACAG<br>CGGAATAAACAGCGACGAGCAGGAGCTCAT	**MAGSHHHHHHGMASMT**<br>**GGQQMGRSGDDDDK**VIK<br>IRYPDDGQWPEAPIDGDGD<br>GNPEFYIEINPWNIQSAEGY<br>AEMTYNLSTGVLHYVQAL<br>DDITLKNGGSWVHGYPEIF<br>YGNKPWNNNYATDGEVPL<br>PGKVSNLSNFYLSVSYKLL<br>PKNGLPINFAIESWLTREP<br>WRNSGINSDEQELMIWLY<br>YDGLQPAGSKVKEIIVPIVV<br>NGTPVNATFEVWKANIGW<br>EYIAFRIKTPIKEGTVTIPYG<br>AFISAAANVTSLANYTELY<br>LEDVEVGTAYGTPSTTSAH<br>LEWWFYNVSLEYRPGEPL<br>LSQPPAEGSAPs

	GATATGGCTGTATTACGACGGACTCCAGCC<br>GGCTGGCTCAAAGGTCAAGGAAATCATTGT<br>CCCGATAGTGGTGAACGGCACCCCAGTGAA<br>CGCTACCTTCGAAGTCTGGAAGGCGAACAT<br>CGGCTGGGAGTACATAGCCTTCAGGATAAA<br>GACCCCAATAAAGGAGGGAACCGTCACTAT<br>ACCGTACGGAGCCTTCATCAGCGCCGCCGC<br>AAACGTAACGAGCCTAGCTAACTACACCGA<br>GCTGTACCTGGAAGACGTTGAGGTTGGAAC<br>CGcATACGGAACGCCCTCAACCACTAGCGC<br>ACACCTCGAGTGGTGGTTCTACAACGTCTCG<br>CTCGAGTACAGGCCTGGAGAGCCACTGCTC<br>TCACAGCCACCTGCGGAAGGGTCTGCTCCA<br>TCA**TAA**

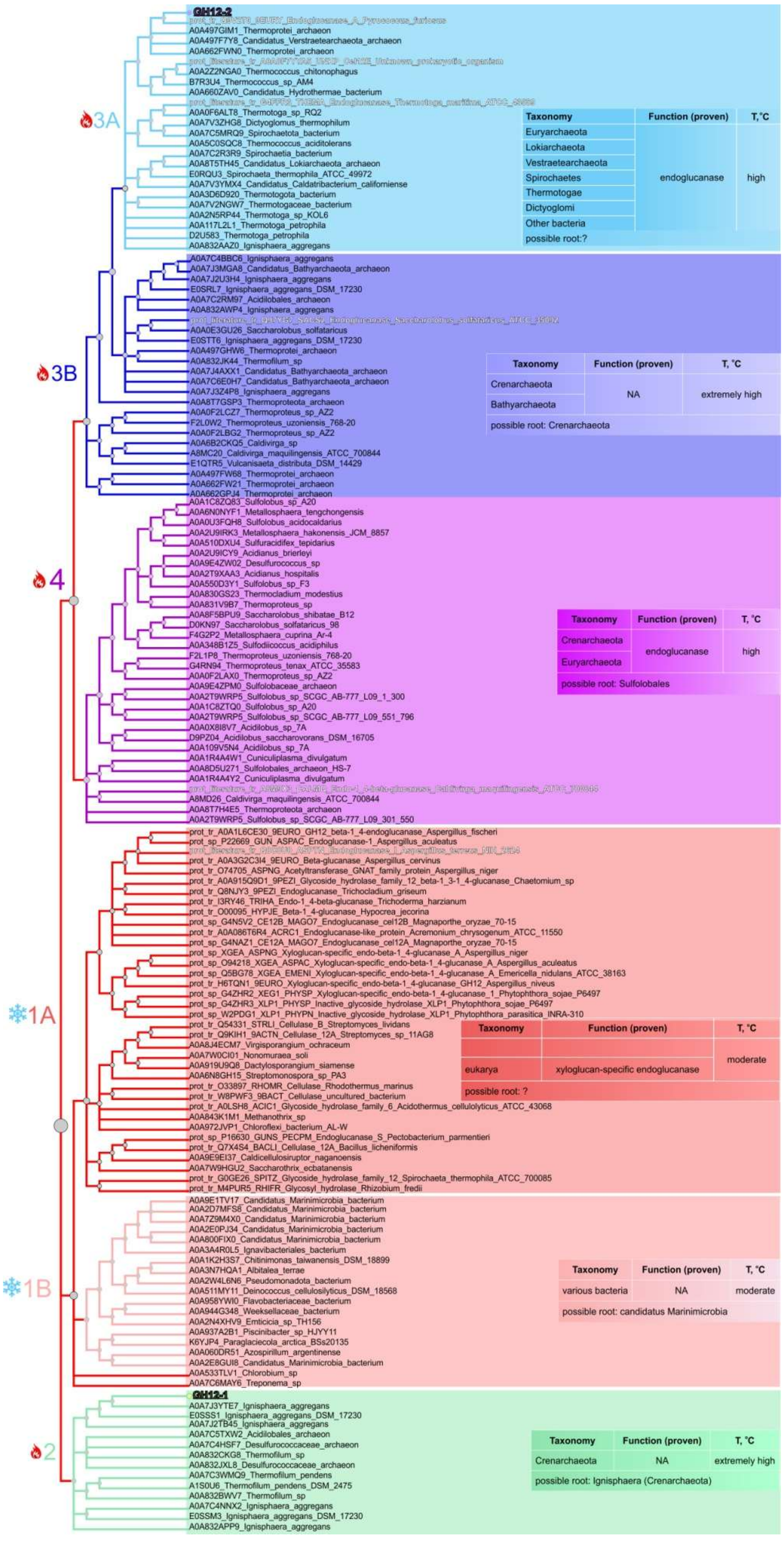

Figure S1. Detailed maximum likelihood phylogenetic tree of GH12 glycosidases. The tree is a consensus tree constructed from two phylogenetic trees inferred using Mafft and CLUSTAL_omega multiple sequence alignemnts. Clusters are named as shown on Fig.2. TMDG_GH12-1 and TMDG_GH12-2 are in green and blue boxes, respectively. Characterized proteins are marked by white color. On the right side of the tree information about GH12 proteins, their origin and functions is presented in colored boxes.

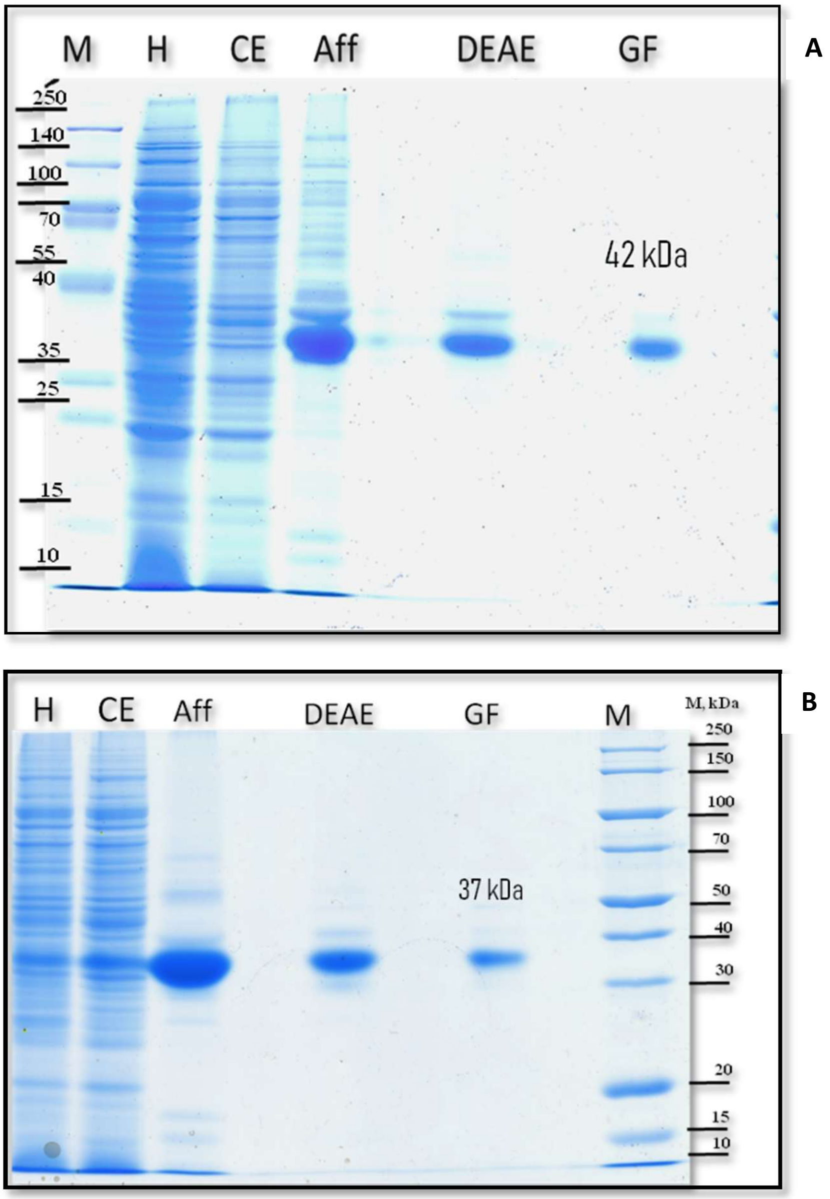

Figure S2. SDS-PAGE of TMDG_GH12 domains. A – TMDG_GH12-1 domain; B – TMDG_GH12-2 domain. M, protein markers; H-homogenate; CE – cell extract; Aff – enzyme preparation obtained after affinity chromatography; DEAE – enzyme preparation obtained after after ion-exchange chromatography; GF – enzyme preparation obtained after size exclusion chromatography.

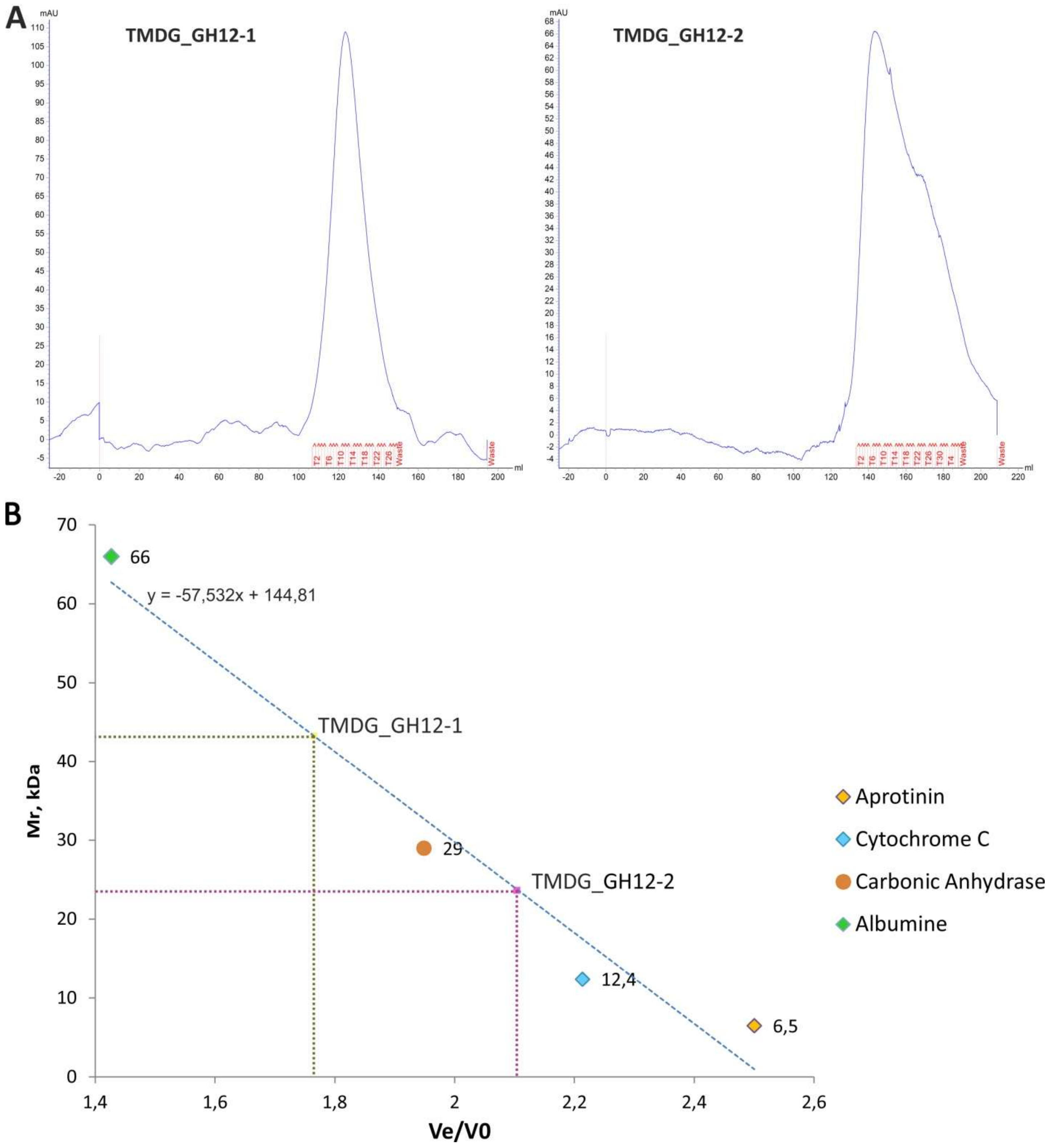

Figure S3. Size exclusion chromatography of GH12 domains.

A – Absorbance at 280 nm in milli-absorbance units (mAU) of the TMDG_GH12 preparations during size-exclusion chromatography. TMDG_GH12-1 fractions T5-T20 (total volume 2.5 ml) and TMDG_GH12-2 fractions T2-T18 (total volume 3 ml) were collected for biochemical characterization of the respective enzymes.

B – Calibration curve inferred using the proteins from the MWGF70 Kit (colored symbols) run on Sephadex G-100 column at the same conditions as purified TMDG_GH12-1 and TMDG_GH12-2. The concentrations of the proteins were: Aprotinin, 3 mg/ml; Cytochrome C 2, mg/ml; Carbonic Anhydrase, 2 mg/ml; Albumin, 5 mg/ml; TMDG_GH12-1, 3.4 mg/ml; TMDG_GH12-2, 2.1 mg/ml. Mr, molecular mass (in kilodaltons); V0, void volume; Ve, elution volume. Green and purple dotted lines indicate the measured Ve/V0 values and calculated using the calibration curve Mr of the TMDG_GH12-1 and TMDG_GH12-2, respectively.

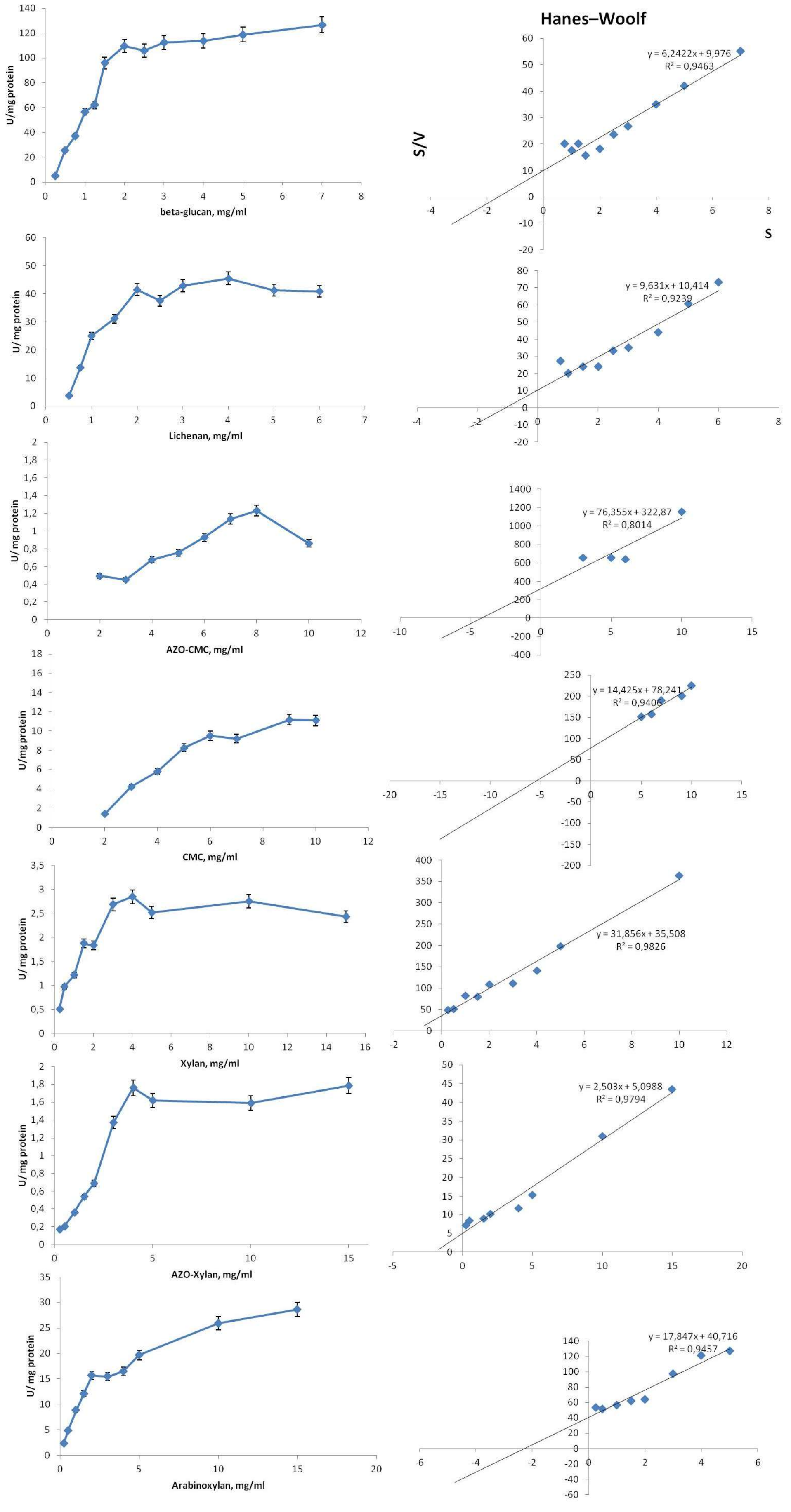

Figure S4. Substrate specificity of TMDG_GH12-1protein. Kinetic parameters were calculated using the Hanes-Woolf plot.

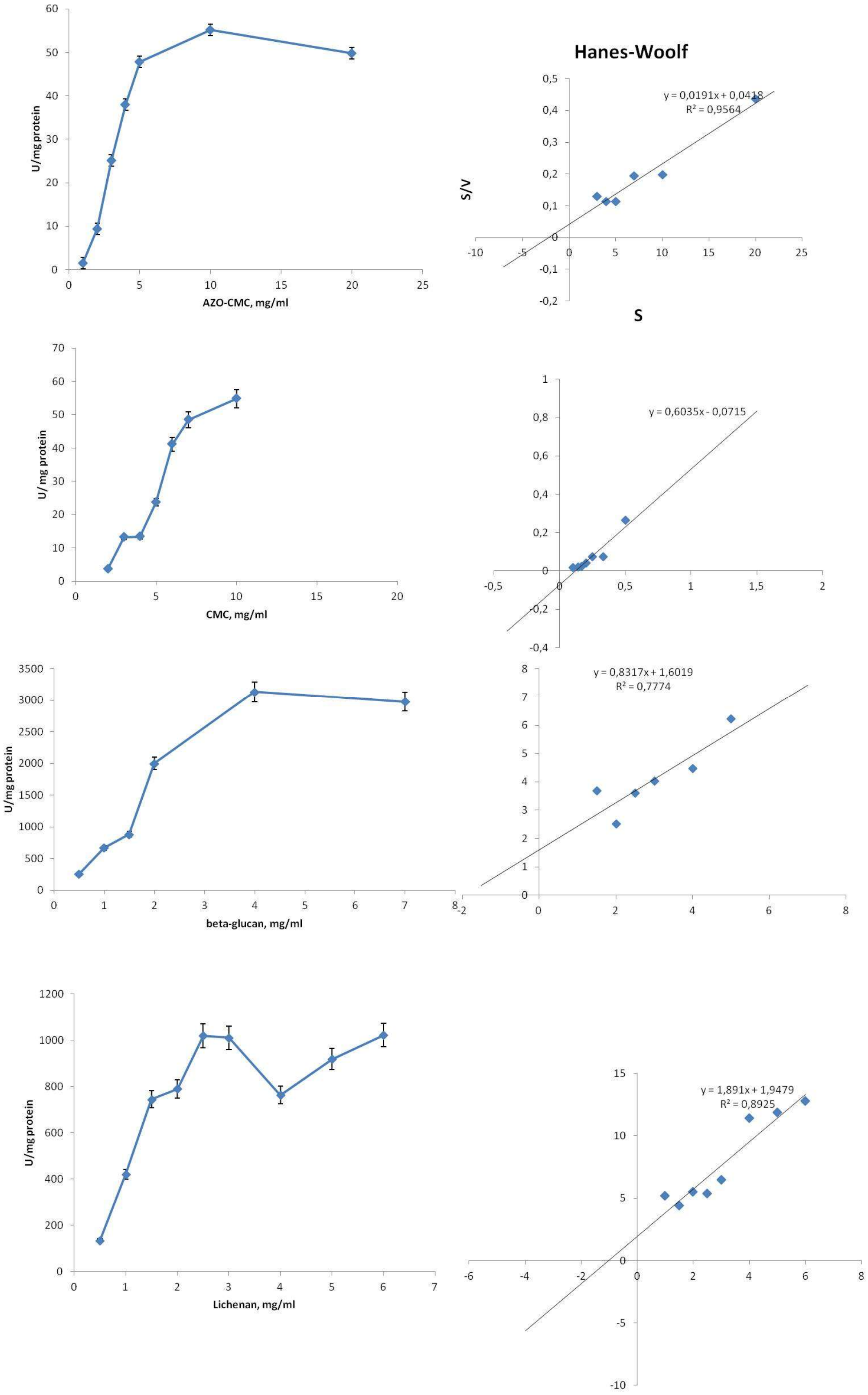

Figure S5. Substrate specificity of TMDG_GH12-2 protein. Kinetic parameters were calculated using the Hanes-Woolf plot.

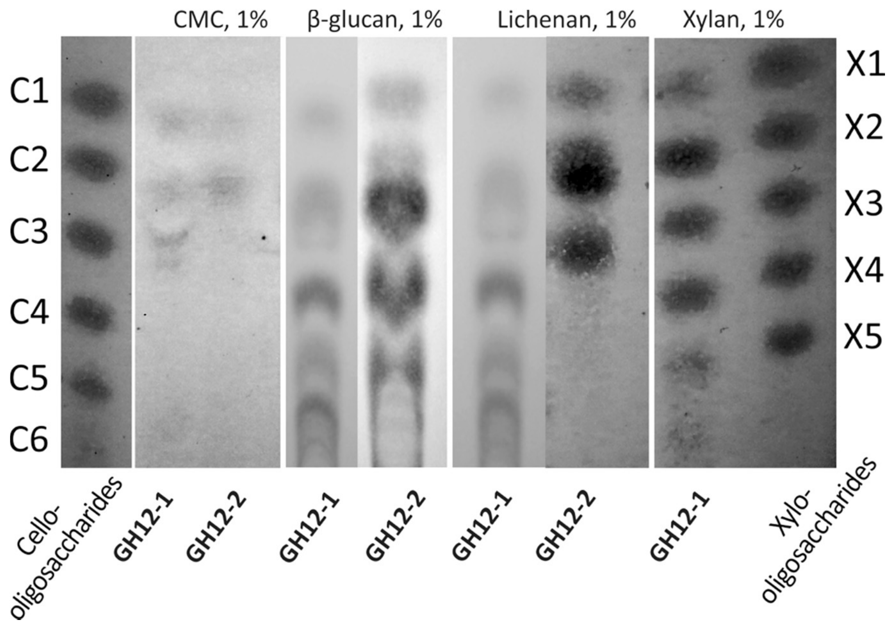

Figure S6. Thin layer chromatography (TLC) of sugars released during hydrolysis of CMC, β-glucan, lichenan and xylan by the TMDG_GH12-2 proteins. The substrates are shown at the top of the figure and the enzymes are shown at the bottom of the figure. Left column – a mixture of celllooligosaccharides (C1-C6), right column - a mixture of xylooligosaccharides (X1-X5).

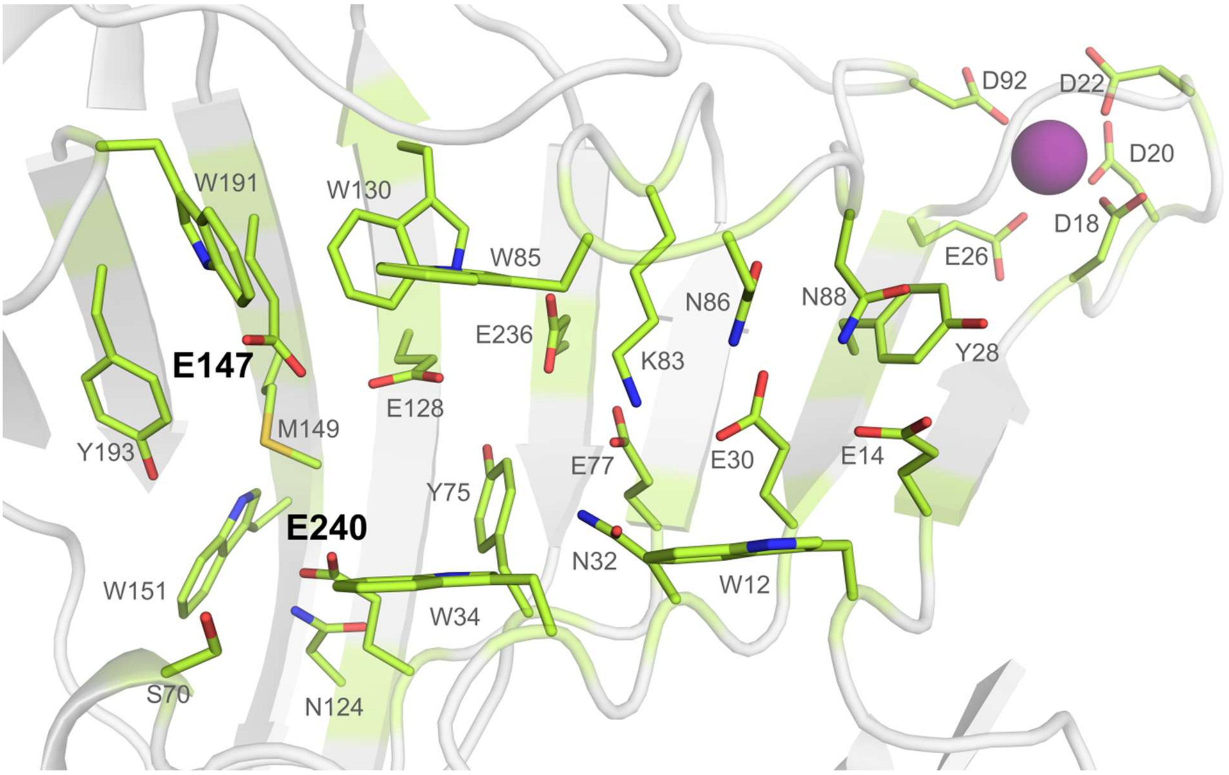

Figure S7. Active site of the TMDG_GH12-2 domain: close-up view. The protein ribbon is colored in gray with amino acid side chains shown as sticks and carbon atoms colored in green. The catalytic residues are indicated by a larger font size: Glu147 (nucleophile) and Glu240 (general acid/base). The Ca^{2+} ion (Ca) is bound in the high-affinity Ca^{2+}-binding site and is shown as a sphere (colored in magenta). This figure was generated using PyMol 2.5.0 (https://pymol.org/).

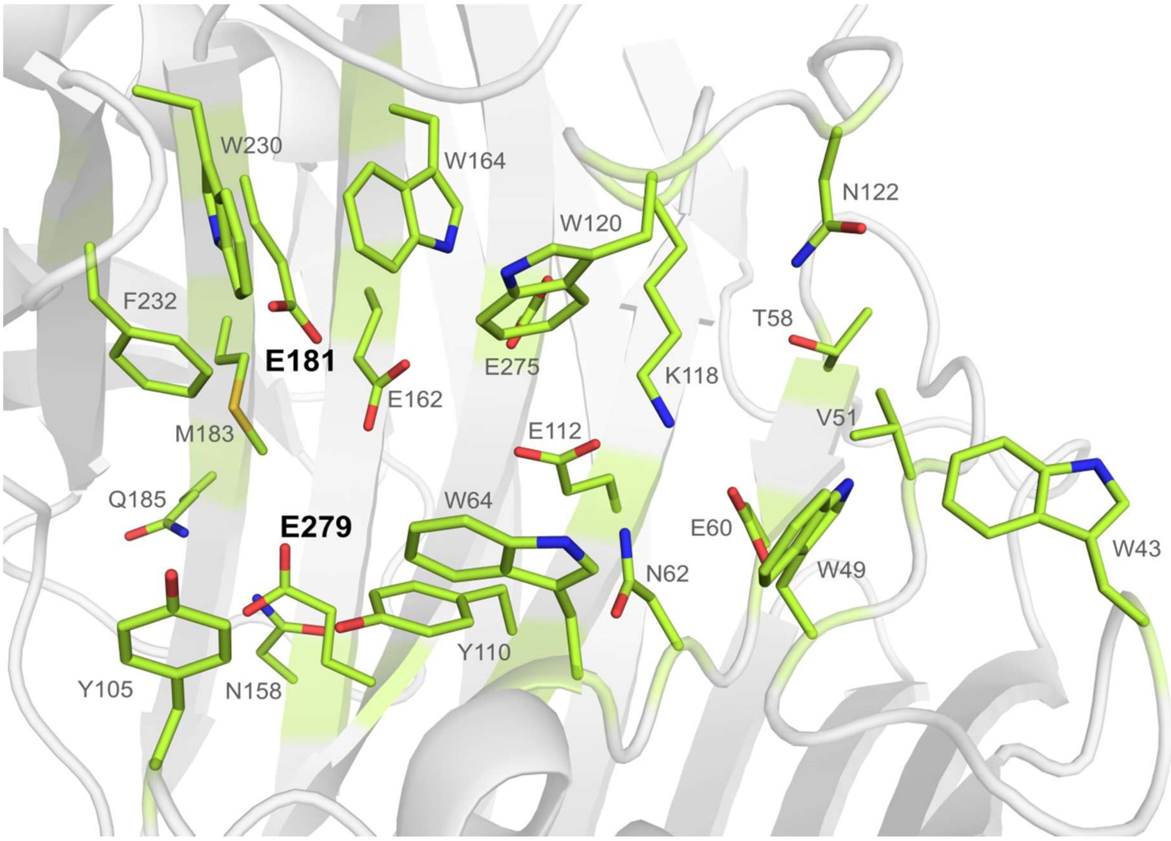

Figure S8. Active site of TMDG_GH12-1 domain: close-up view. The protein ribbon is colored in gray with amino acid side chains shown as sticks and carbon atoms colored in green. The catalytic residues are indicated by a larger font size: Glu181 (nucleophile) and Glu279 (general acid/base). This figure was generated using PyMol 2.5.0 (https://pymol.org/).

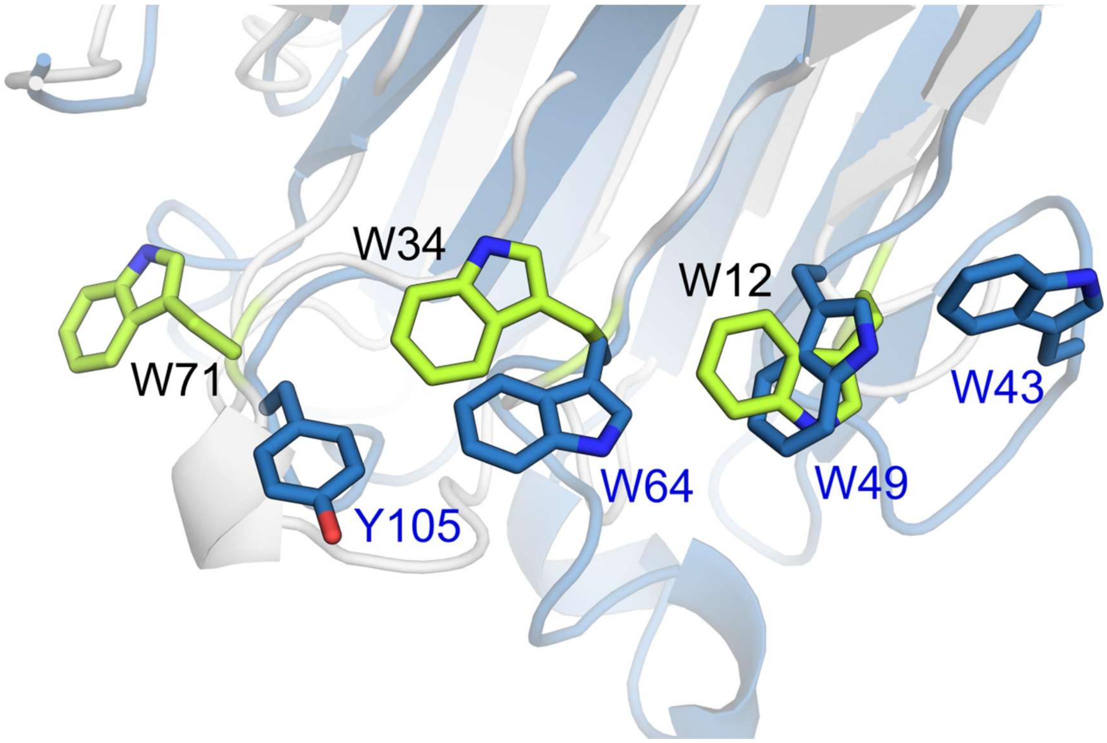

FigureS9. Different location of tryptophan moities in the reaction cavities of TMDG_GH12-1 and TMDG_GH12-2. Green color – TMDG_GH12-2, blue – TMDG_GH12-1. This figure was generated using PyMol 2.5.0 (https://pymol.org/).

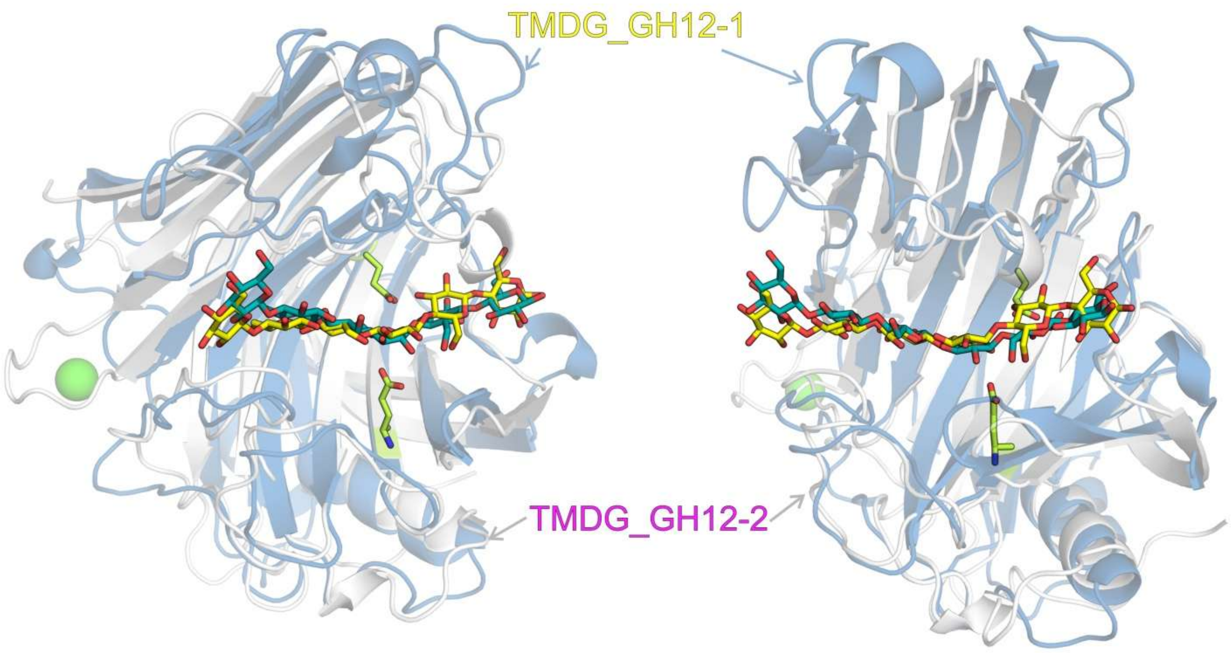

Figure S10. Cellohexaose docking into the active sites of TMDG_GH12-1 and TMDG_GH12-2 domains. The protein ribbon diagrams are shown in blue (TMDG_GH12-1) and gray (TMDG_GH12-2) with the key active site residues shown as sticks with green colored carbons. Cellohexaose molecules positions are shown as sticks with yellow colored carbons and teal for TMDG_GH12-1 and TMDG_GH12-2, respectively, whereas the green sphere represents the bound Ca^{2+} ion. This figure was generated using PyMol 2.5.0 (https://pymol.org/).

Supplemented References:

1. Bauer MW, Driskill LE, Callen W, Snead MA, Mathur EJ, & Kelly RM (1999) An endoglucanase, EglA, from the hyperthermophilic archaeon *Pyrococcus furiosus* hydrolyzes beta-1,4 bonds in mixed-linkage (1-->3),(1-->4)-beta-D-glucans and cellulose. *Journal of bacteriology* 181(1), 284–290.
2. Leis B, Heinze S, Angelov A, Pham VT, Thürmer A, Jebbar M, Golyshin PN, Streit WR, Daniel R & Liebl W (2015) Functional Screening of Hydrolytic Activities Reveals an Extremely Thermostable Cellulase from a Deep-Sea Archaeon. *Frontiers in bioengineering and biotechnology* 3, 95.
3. Huang Y, Krauss G, Cottaz S, Driguez H, & Lipps G (2005). A highly acid-stable and thermostable endo-beta-glucanase from the thermoacidophilic archaeon *Sulfolobus solfataricus*. *The Biochemical journal* 385(Pt 2), 581–588.
4. Maurelli L, Giovane A, Esposito A, Moracci M, Fiume I, Rossi M & Morana A (2008) Evidence that the xylanase activity from *Sulfolobus solfataricus* Oalpha is encoded by the endoglucanase precursor gene (sso1354) and characterization of the associated cellulase activity. *Extremophiles* 12, 689–700.
5. Boyce A & Walsh G (2018) Expression and characterisation of a thermophilic endo-1,4-β-glucanase from *Sulfolobus shibatae* of potential industrial application. *Mol Biol Rep* 45, 2201–2211.
6. Wang H, Squina F, Segato F, Mort A, Lee D, Pappan K, & Prade R (2011). High-temperature enzymatic breakdown of cellulose. *Applied and environmental microbiology* 77(15), 5199–5206.
7. Suleiman M, Schröder C, Klippel B, Schäfers C, Krüger A & Antranikian G (2019) Extremely thermoactive archaeal endoglucanase from a shallow marine hydrothermal vent from Vulcano Island. *Appl Microbiol Biotechnol* 103, 1267–1274.
8. Ando S, Ishida H, Kosugi Y & Ishikawa K (2002) Hyperthermostable endoglucanase from *Pyrococcus horikoshii*. *Appl Environ Microbiol* 68, 430–433.
9. Strazzulli A, Cobucci-Ponzano B, Iacono R, Giglio R, Maurelli L, Curci N, Schiano-di-Cola C, Santangelo A, Contursi P, Lombard V, Henrissat B, Lauro FM, Fontes CMGA, Moracci M. (2020) Discovery of hyperstable carbohydrate-active enzymes through metagenomics of extreme environments. *FEBS J*. 287(6), 1116-1137.
10. Graham JE, Clark ME, Nadler DC, Huffer S, Chokhawala HA, Rowland SE, Blanch HW, Clark DS & Robb F (2011) Identification and characterization of a multidomain hyperthermophilic cellulase from an archaeal enrichment. *Nat Commun* 2, 375–379.
11. Ilari A, Fiorillo A, Angelaccio S, Florio R, Chiaraluce R, van der Oost J, Consalvi V (2009) Crystal structure of a family 16 endoglucanase from the hyperthermophile *Pyrococcus furiosus*--structural basis of substrate recognition. *FEBS J* 276(4), 1048-1058.
12. Gavrilov SN, Stracke C, Jensen K, Menzel P, Kallnik V, Slesarev A, Sokolova T, Zayulina K, Bräsen C, Bonch-Osmolovskaya EA, Peng X, Kublanov IV, Siebers B (2016) Isolation and characterization of the first xylanolytic hyperthermophilic euryarchaeon *Thermococcus* sp. strain 2319x1 and its unusual multidomain glycosidase. *Front Microbiol* 7, 552.
13. Liebl W, Ruile P, Bronnenmeier K, Riedel K, Lottspeich F, & Greif I. (1996) Analysis of a *Thermotoga maritima* DNA fragment encoding two similar thermostable cellulases, CelA and CelB, and characterization of the recombinant enzymes. *Microbiology* 142 (9), 2533–2542.
14. Halldórsdóttir S, Thórólfsdóttir ET, Spilliaert R, Johansson M, Thorbjarnardóttir SH, Palsdottir A, Hreggvidsson GO, Kristjánsson JK, Holst O & Eggertsson G (1998) Cloning, sequencing and overexpression of a *Rhodothermus marinus* gene encoding a thermostable cellulase of glycosyl hydrolase family 12. *Applied microbiology and biotechnology* 49(3), 277–284.
15. Saarilahti HT, Henrissat B & Palva E T (1990) CelS: a novel endoglucanase identified from *Erwinia carotovora* subsp. carotovora. *Gene* 90(1), 9–14.

16. Gloster TM, Ibatullin FM, Macauley K, Eklöf JM, Roberts S, Turkenburg JP, Bjørnvad ME, Jørgensen PL, Danielsen S, Johansen KS, Borchert TV, Wilson KS, Brumer H Davies GJ (2007) Characterization and three-dimensional structures of two distinct bacterial xyloglucanases from families GH5 and GH12. *The Journal of biological chemistry* 282(26), 19177–19189.
17. van Solingen P, Meijer D, van der Kleij WA, Barnett C, Bolle R, Power SD Jones BE (2001) Cloning and expression of an endocellulase gene from a novel streptomycete isolated from an East African soda lake. *Extremophiles : life under extreme conditions* 5(5), 333–341.
18. Angelov A, Loderer C, Pompei S & Liebl W (2011) Novel family of carbohydrate-binding modules revealed by the genome sequence of *Spirochaeta thermophila* DSM 6192. *Applied and environmental microbiology* 77(15), 5483–5489.
19. Wang J, Gao G, Li Y, Yang L, Liang Y, Jin H, Han W, Feng Y & Zhang Z (2015) Cloning, Expression, and Characterization of a Thermophilic Endoglucanase, AcCel12B from *Acidothermus cellulolyticus* 11B. *International journal of molecular sciences* 16(10), 25080–25095.
20. Hengge NN, Mallinson SJB, Pason P, Lunin VV, Alahuhta M, Chung D, Himmel ME, Westpheling J Bomble YJ (2022) Characterization of the Biomass Degrading Enzyme GuxA from *Acidothermus cellulolyticus*. *International journal of molecular sciences* 23(11), 6070.
21. Woo Park Y, Tech Lim S & Dae Yun H (1998) Cloning and characterization of a CMCase gene, celB, of *Erwinia carotovora* subsp. carotovora LY34 and its comparison to celA. *Molecules and cells* 8(3), 280–285.
22. Wittmann S, Shareck F, Kluepfel D & Morosoli R (1994) Purification and characterization of the CelB endoglucanase from *Streptomyces lividans* 66 and DNA sequence of the encoding gene. *Applied and environmental microbiology* 60(5), 1701–1703.
23. Amore A, Pepe O, Ventorino V, Birolo L, Giangrande C & Faraco V (2012) Cloning and recombinant expression of a cellulase from the cellulolytic strain *Streptomyces* sp. G12 isolated from compost. *Microbial cell factories* 11, 164.
24. Bok JD, Yernool DA & Eveleigh DE (1998) Purification, characterization, and molecular analysis of thermostable cellulases CelA and CelB from *Thermotoga neapolitana*. *Applied and environmental microbiology* 64(12), 4774–4781.
25. Okano H, Ozaki M, Kanaya E, Kim JJ, Angkawidjaja C, Koga Y & Kanaya S (2014) Structure and stability of metagenome-derived glycoside hydrolase family 12 cellulase (LC-CelA) a homolog of Cel12A from *Rhodothermus marinus*. *FEBS open bio* 4, 936–946.
26. Yeh YF, Chang SC, Kuo HW, Tong CG, Yu SM & Ho TH (2013) A metagenomic approach for the identification and cloning of an endoglucanase from rice straw compost. *Gene* 519(2), 360–366.
27. Segato F, Berto G, Ares de Araújo E, Muniz JR & Polikarpov I (2014) Expression, purification, crystallization and preliminary X-ray diffraction analysis of *Aspergillus terreus* endo-β-1,4-glucanase from glycoside hydrolase family 12. *Acta crystallographica. Section F, Structural biology communications* 70(2), 267–270.
28. van Peij NN, Gielkens MM, de Vries RP, Visser J & de Graaff LH (1998) The transcriptional activator XlnR regulates both xylanolytic and endoglucanase gene expression in *Aspergillus niger*. *Applied and environmental microbiology* 64(10), 3615–3619.
29. Rawat R, Kumar S, Chadha BS, Kumar D & Oberoi HS (2015) An acidothermophilic functionally active novel GH12 family endoglucanase from *Aspergillus niger* HO: purification, characterization and molecular interaction studies. *Antonie van Leeuwenhoek* 107(1), 103–117.
30. Rykov SV, Selimzyanova AI, Nikolaeva AY, Lazarenko VA, Tsurin NV, Akentyevn , Zverlov VV, Liebl W, Schwarz WH & Berezina OV (2022) Unusual substrate specificity in GH family 12: structure-function analysis of glucanases Bgh12A and Xgh12B from *Aspergillus cervinus*, and Egh12 from *Thielavia terrestris*. *Applied microbiology and biotechnology* 106(4), 1493–1509.
